# EcoTILLING revealed SNPs in *GhSus* genes that are associated with fiber- and seed-related traits in upland cotton

**DOI:** 10.1038/srep29250

**Published:** 2016-07-07

**Authors:** Yan-Da Zeng, Jun-Ling Sun, Su-Hong Bu, Kang-Sheng Deng, Tao Tao, Yuan-Ming Zhang, Tian-Zhen Zhang, Xiong-Ming Du, Bao-Liang Zhou

**Affiliations:** 1 State Key Laboratory of Crop Genetics & Germplasm Enhancement, Nanjing Agricultural University, Nanjing 210095, China; 2 Institute of Cotton Research, Chinese Academy of Agricultural Sciences, Anyang 455000, China; 3 College of Plant Science and Technology, Huazhong Agricultural University, Wuhan 430070, China

## Abstract

Cotton is the most important textile crop in the world due to its cellulose-enriched fibers. Sucrose synthase genes (*Sus*) play pivotal roles in cotton fiber and seed development. To mine and pyramid more favorable alleles for cotton molecular breeding, single nucleotide polymorphisms (SNPs) of *GhSus* family genes were investigated across 277 upland cotton accessions by EcoTILLING. As a result, a total of 24 SNPs in the amplified regions of eight *GhSus* genes were identified. These SNPs were significantly associated with at least one fiber- or seed-related trait measured in Nanjing, Anyang and Kuche in 2007–2009. Four main-effect quantitative trait nucleotides (QTNs) and five epistatic QTNs, with 0.76–3.56% of phenotypic variances explained by each QTN (PVE), were found to be associated with yield-related traits; six epistatic QTNs, with the 0.43–3.48% PVE, were found to be associated with fiber quality-related traits; and one main-effect QTN and one epistatic QTN, with the PVE of 1.96% and 2.53%, were found to be associated with seed oil content and protein content, respectively. Therefore, this study provides new information for molecular breeding in cotton.

Cotton fiber is not only the most important textile material worldwide, but also the ideal experimental system for studying the mechanism of cell development due to its single-celled profile^1^. Cotton fibers originate from the epidermal cells of ovules, and its growth and development is a highly regulated process with four distinct, but overlapping, stages: initiation, elongation, secondary wall synthesis, and maturation^2^. During the development of cotton fiber, the importation and metabolism of sucrose is considered to be a major factor in determining the sink strength of tissues^3^. In higher plants, sucrose is the major product of photosynthesis, the main form of translocated carbon, and the main substrate of sink metabolism^4^. Sucrose metabolism and accumulation in plant cells involves two key enzymes: sucrose synthase (Sus, EC 2.4.1.13) and invertase (INV, EC 3.2.1.26). Sucrose synthase catalyzes a reversible reaction, but preferentially cleaves sucrose to bond uridine diphosphate (UDP) into UDP-glucose and fructose^5^. Sucrose synthase plays important roles in metabolic processes, including starch storing^6^,^7^,^8^,^9^,^10^, cellulose synthesis, sugar import^11^,^12^, environmental stress responses^13^,^14^, and nitrogen fixation, as well as arbuscule maturation and maintenance in mycorrhizal roots of legumes^15^,^16^.

Sucrose synthase (*Sus*) genes have been proposed to be involved in controlling cotton fiber development^17^. *Sus* activity is responsible for cellulose synthesis by supplying the UDP-glucose substrate, which is essential for cell wall thickening and cotton fiber cell development^18^,^19^,^20^. Suppressing *Sus* activity in the ovule epidermis led to a fiberless phenotype and to fewer fiber initials or shrunken or collapsed fibers in those ovules in cotton^21^. Over-expressing a *Sus* gene from potato in cotton gave rise to enhanced leaf development, improved early seed development, and promoted fiber elongation^22^. Thus, a new strategy was proposed to increase cotton fiber yield by improving seed development. As demonstrated in many plants, *Sus* isoforms are encoded by a multiple gene family. For example, maize and pea each contain three distinct *Sus* family members^6^,^23^. Six different *Sus* genes were identified in *Arabidopsis thaliana*, *Oryza sativa*, *Hevea brasiliensis*, and *Citrus unshiu*^13^,^24^,^25^,^26^. *Populus* has seven *Sus* genes^27^,^28^. In two diploid cotton species (*Gossypium arboreum* L. and *G. raimondii* Ulbr.), eight *Sus* genes for each species have been found^29^. The tetraploid cotton, *G. hirsutum* L., has the largest *Sus* family to date, containing fifteen *Sus* genes^29^. Examination of their expression patterns indicated that *Sus* gene family members have different expression in various tissues or organs in cotton species. Additionally, their functional analyses and isolations were confined to a limited number of cotton accessions, their diverse functions and sequence variations have not been clarified in natural populations, implying that some favorable alleles are yet to be identified for cotton.

EcoTILLING is a fast and cost-effective method of detecting rare SNPs, small insertions, and deletions (indels) in target genes in natural populations, and was adapted from Targeting Induced Local Lesions in Genomes (TILLING)^30^. Compared to direct sequencing, EcoTILLING has the following advantages: high-throughput, accuracy, and cost-effectiveness. To date, EcoTILLING has been used for many purposes, including mapping^31^,^32^, candidate gene discovery^33^,^34^, evaluation of nucleotide diversity^35^, and population genetics^36^,^37^.

In this study, EcoTILLING technology was used to reveal sequence diversities and identify SNPs in *GhSus* family genes in a natural population of upland cotton accessions. The SNPs were then used for association analysis of phenotype variations in plants from nine environments over three years and three locations, and for the identification of favorable *GhSus* alleles that confer high yield and high fiber and seed quality traits.

## Results

### Nucleotide polymorphisms

Fifteen genes in the *GhSus* family were analyzed by EcoTILLING. As a result, eight out of 15 genes showed a total of 24 putative natural variation sites in the amplified regions across 277 upland cotton accessions, and no SNPs were detected in the other seven genes (Table 1). The minor allele frequency of polymorphic sites in the 277 accessions ranged from 0.018 to 0.755, with an average 0.214. Samples containing each of the polymorphic sites were randomly sequenced to determine the position and identity of these polymorphic sites in the targeted region of genes. Twenty four putative SNPs were confirmed by sequencing in the targeted region of the eight *GhSus* family members. Of the 24 SNPs, 12 were in the coding regions, including seven synonymous variations and five non-synonymous variations. Of the five non-synonymous variations, two SNPs were predicted by the SIFT program to severely affect protein function (Table 1).

### Haplotype diversity analysis

The haplotype distribution for each of the amplicons is shown in Table 2. The *GhSus1At* amplicon had the largest number of haplotypes in the *GhSus* gene family, with eight haplotypes detected, while the amplicons *GhSus4Dt* and *GhSus7Dt* each had only two haplotypes. The levels of haplotype diversity (HD) varied markedly in different amplicons of the *GhSus* family. The highest level of HD was in amplicon *GhSus1At*, with 0.574, while the lowest was in amplicon *GhSus4Dt*, with only 0.049. One major haplotype was detected in each *GhSus* gene sequence except for amplicon *GhSus1At*, for which no haplotype exhibited a frequency higher than 0.5 (Supplementary Table S1).

### Population structure

Analysis of the population structure composed of 277 upland cotton varieties showed that the log probability of data [In *P*(*D*)] value corresponding to each hypothetical number of subpopulations (*k*) continued to increase with *k* value and did not reach a peak. An ad hoc statistic ∆*k* value was then calculated according to the method described by Evanno *et al.*^38^. The values showed a much higher likelihood at *k* = 2 than at *k* = 3–10, suggesting that the whole panel could be divided into two major subpopulations, SP1 and SP2, respectively. The SP1 group contained 89 accessions; 16 from America, three from Europe, seven from North regions of China, 12 from Northwest regions of China, 32 from the Yellow River region of China, 15 from the Yangtze River regions of China, and four from other Asian regions. The SP2 group consisted of 188 accessions; four from Australia, five from Africa, 34 from America, two from Europe, six from North regions of China, 3 from Northwest regions of China (Supplementary Table S2), 101 from the Yellow River region of China, 33 from the Yangtze River regions of China. The corresponding Q matrix at *k* = 2 was then used for the subsequent association analysis.

Complete linkage disequilibrium of SNPs was detected as follows within five single *GhSus* genes: *GhSus1Dt*-T650C and *GhSus1Dt*-G751A in *GhSus1Dt*-G751A; *GhSus4Dt*-A1886T and *GhSus4Dt*-T2167C in *GhSus4Dt*; *GhSus3At*-T881A, *GhSus3At*-T1320C, and *GhSus3At*-G1327A in *GhSus3At*; *GhSus3At*-T2294C and *GhSus3At*-A2472C in *GhSus3At*; *GhSus6At*-C4655T, *GhSus6At*-G4697A, and *GhSus6At*-G5005A in *GhSus6At*; *GhSus7Dt*-T192C, *GhSus7Dt*-C404T, and *GhSus7Dt*-T407C in *GhSus7Dt*. All SNPs in complete linkage disequilibrium were combined into a single haplotypes and those were used for further analysis (here, for easy description of the results, only one SNP name from each haplotype was used instead of haplotype). Although ten of those SNPs (for example, *GhSus1At*-A918G and *GhSus1At*-G1783C) result in synonymous variations or are located in intron regions, they may be in complete linkage disequilibrium with other non-synonymous mutations, which could have functional relevance to the organism. Therefore, these SNPs were further analyzed. Thus, a total of 15 SNPs were included in the association analysis, namely, three SNPs in *GhSus1At* (*GhSus1At*-A918G, *GhSus1At*-G1783C, and *GhSus1At*-A2940T), each two SNPs in *GhSus1Dt* (*GhSus1Dt*-G751A and *GhSus1Dt*-G2709C), in *GhSus3At* (*GhSus3At*-T881A and *GhSus3At*-T2294C), in *GhSus5Dt* (*GhSus5Dt*-G491T and *GhSus5Dt*-C2648G), in *GhSus6At* (*GhSus6At*-G3143T and *GhSus6At*-C4655T) and in *GhSus8Dt* (*GhSus8Dt*-C577T and *GhSus8Dt*-T1334C), and each one in *GhSus4Dt* (*GhSus4Dt*-A1886T) and in *GhSus7Dt* (*GhSus7Dt*-T192C).

### Association between SNPs and fiber- or seed-related traits

In this study, eleven SNPs were found to be significantly associated with traits (four SNPs were main-effect QTNs and eleven pairs of SNPs were involved in epistatic QTNs) (Table 3). Of them, six QTNs were associated with fiber quality traits. Three epistatic QTNs (*GhSus1At*-A918G × *GhSus5Dt*-C2648G, *GhSus1At*-A918G × *GhSus6At*-C4655T, and *GhSus1Dt*- G751A × *GhSus3At*-T881A) were associated with fiber length (FL), which explained 3.48%, 1.09%, and 1.50% of the phenotypic variation (PVE), respectively. Two epistatic QTNs (*GhSus1Dt*-G751A × *GhSus8Dt*-T1334C and *GhSus1Dt*-G2709C × *GhSus8Dt*-C577T) were associated with fiber micronaire (FM), which explained 1.27% and 0.89% of PVE, respectively. One epistatic QTN (*GhSus3At*-T881A × *GhSus7Dt*-T192C) was associated with fiber strength (FS), which explained 0.43% of PVE. Nine QTNs were associated with yield traits. One main-effect QTN (*GhSus1At*-A2940T) was associated with lint percentage (LP), which explained 3.43% of PVE. Two main-effect QTNs (*GhSus1At*-G1783C and *GhSus3At*-T881A) and two epistatic QTNs (*GhSus1At*-A2940T × *GhSus1Dt*-G2709C and *GhSus6At*-C4655T × *GhSus7Dt*-T192C) were associated with boll weight (BW), which explained 3.56% and 1.76%, and 2.03% and 0.76% of PVE, respectively. One main-effect QTN (*GhSus3At*-T881A) and three epistatic QTNs (*GhSus1At*-A918G × *GhSus1Dt*-G2709C, *GhSus1At*-G1783C × *GhSus1Dt*- G2709C, and *GhSus1Dt* -G751A × *GhSus1Dt*-G2709C) was associated with seed index (SI), which explained 1.32% and 2.10%, 2.27%, and 0.89% of PVE, respectively. One epistatic QTN (*GhSus1At*-A918G × *GhSus1Dt*-G2709C) and one main-effect QTN (*GhSus6At*-C4655T) were associated with protein content (PC) and oil content (OC), which explained 2.53% and 1.96% of PVE, respectively. No significant environmental interactions were found.

Using epistatic association analyses, seven pairs of *GhSus* family genes, namely, *GhSus1At* × *GhSus1Dt* (BW, PC, and three for SI), *GhSus6At* × *GhSus7Dt* (BW), *GhSus1At* × *GhSus5Dt* (FL), *GhSus1At* × *GhSus6At* (FL), *GhSus1Dt* × *GhSus3At* (FL), *GhSus3At* × *GhSus7Dt* (FS), *GhSus1Dt* × *GhSus8Dt* (two for FM) were found to have epistatic interactions (Table 3). Among all the seven epistatic interactions, two-way ANOVA was used to further detect the corresponding interactions of these gene haplotypes. As a result, six pairs of gene haplotypes were found to have epistatic interactions except for *GhSus3At* × *GhSus7Dt* (FS) (Supplementary Table S3).

### Favorable QTN alleles mined in upland cotton accessions

The phenotypic effects of each QTN allele were estimated by the method of Lü *et al.*^39^, and a total of 17 desirable alleles were identified. Phenotypic effects for each favorable allele are shown in Table 3. Among the favorable alleles, *GhSus3At-881A* had the most positive phenotypic effects for BW and SI, and increased BW and SI by 0.23 g and 0.40 g, respectively. One epistatic QTN, between *GhSus1At-918G* and *GhSus5Dt-2648G*, increased FL by 0.42 mm; another epistatic QTN, between *GhSus1Dt-2709G* and *GhSus8Dt-577C*, decreased FM by 0.08; *GhSus1At-2940T* increased LP by 0.95%; the epistatic QTN between *GhSus3At-881A* and *GhSus7Dt-192C* increased FS by 0.19 cN/tex; the epistatic QTN between *GhSus1At-918A* and *GhSus1Dt-2709G* increased PC by 0.49%; and *GhSus6At-4655C* increased OC by 0.54%.

### Expression analysis of *GhSus* gene at different developmental stages

To better understand the potential functions of *GhSus* isoforms, their expression patterns were analyzed in fiber and ovule at different developmental stages using upland cotton TM-1 published RNA-seq data^40^. Most of *GhSus* genes were expressed (fragments per kilobase of transcript per million reads sequence (FPKM) > 1) in both fiber and ovule except for *GhSus4A* and *GhSus4D* which were not expressed in either tissue. The expression levels of *GhSus1A* and *GhSus1D* were both high in fiber at 5 DPA (days post-anthesis), then declined notably at 20 DPA (Fig. S1). The transcripts of *GhSus5D* and *GhSus8D* showed relatively higher in fiber from 5 to 25 DPA, and were very weak in ovule (Fig. S1).

## Discussion

To understand genetic variation and mine more favorable alleles, 277 upland cotton accessions from China and another 5 geographic areas were analyzed for allele diversity in fifteen *GhSus* family genes by EcoTILLING. The results revealed 24 SNPs among the family genes after analysis of 14,957,446 bp sequences. Of the 24 SNPs, half (12/24) were in coding regions, including seven synonymous variations and five nonsynonymous variations. Of the five nonsynonymous variations, two SNPs were predicted by the SIFT program to severely affect protein function. Although SIFT has a 20% false-positive error and some mutations predicted to be deleterious may be functionally neutral, these scores may be useful in prioritizing mutations for further study and for the analysis of possible contributions of the *GhSus* family gene members to stress tolerance.

Sus is a key enzyme in plant sucrose metabolism. In many plants, Sus isoforms are encoded by a small multi-gene family^13^,^24^. Fifteen *Sus* genes have been detected in *G. hirsutum* so far; previous reports showed that some of these genes were related to the initiation, elongation, and secondary cell wall deposition of the single-celled fibers^41^,^42^,^43^,^44^. However, little is known about the underlying genetics and molecular biology of the *Sus* genes and their physiological functions during fiber growth and development.

In the present study, eleven SNPs in the *GhSus* family gene members were significantly associated with at least one agronomic trait. Five main-effect QTNs and twelve epistatic QTNs containing these eleven SNPs were detected for eight traits in upland cotton. Although most of the QTNs were associated with SNPs that are expected to result in synonymous mutations or be located in intron sequences, these SNPs may be in complete linkage disequilibrium with other non-synonymous mutations, which could have functional relevance to the organism, or these SNPs could become the targets of other genes, leading to effects on agronomic traits. All of these are waiting for further experimental confirmation.

Using 2,878 QTLs mapped in a previous study^40^, we found that only five out of eleven SNPs in our study were located in the same regions as previous reports. For example, the locus *GhSus1Dt-G751A* associated with FL on chromosome (Chr.) 19 was located in the region of qFL-D5-1 identified by Hu *et al.*^45^. The loci *GhSus1Dt-G751A* and *GhSus1Dt-G2709C*, both associated with FM on Chr. 19, were located in the region of qFM-C19-1 identified by Sun *et al.*^46^. The locus *GhSus1Dt-G2709C* associated with PC on Chr. 19 was located in the region of qPro2-c19-1 identified by Yu *et al.*^34^. The locus GhSus5Dt-C2648G associated with FL on chromosome 25 was located in the regions of qFL-C25-1 and qFL-C25-2 identified by Sun *et al.*^46^. The locus GhSus7Dt-T192C associated with BW on chromosome 16 was located in the region of qBW-C16-1 identified by Wu *et al.*^47^. The locus GhSus7Dt-T192C was also associated with FS, which was located in the regions of F2:3-qFS-c16-1 and F2:3-qFS-c16-2 identified by Yu *et al.*^48^. This comparison demonstrates that our association analysis further confirmed the previous results of QTL mapping suggesting that these QTLs are stable, repeatable, and reliable.

The quality and productivity of cotton fiber is mainly affected by two biological processes: fiber initiation and fiber elongation^49^. The fiber elongation process (~3–23 days post anthesis, DPA) determines the fiber final length and strength. Fiber strength also depends on formation of the secondary cell wall (~16–40 DPA), which contains >95% of the dry weight of cellulose in the mature cotton fiber. As *GhSus* genes are vital for fiber cell development, suppression experiments result in a disruption of fiber and seed development^43^. Due to the severity of the phenotypic effects, it was difficult to analyze the impact of different *GhSus* family genes on fiber quality from pleiotropic developmental effects on fiber growth. As a reverse genetic research method, EcoTILLING technology provided a new approach for *GhSus* family gene function analysis.

The expression patterns of *GhSus* family genes during fiber cell development were analyzed using RNA-seq data from *G. hirsutum* TM-1^44^. All seven genes associated with the fiber-related traits (Table 3) were highly expressed in fiber cells during the fiber elongation stage or secondary cell wall formation stage. Five *GhSus* genes (*GhSus1At*, *GhSus1Dt*, *GhSus5Dt*, *GhSus6At*, and *GhSus3At*) associated with fiber length were found to be highly expressed during the fiber elongation process (Supplementary Fig. S1). Two genes (*GhSus3At* and *GhSus7At*) associated with fiber strength had relatively high expression in the secondary cell wall formation stage (~16–40 DPA) (Supplementary Fig. S1). Sucrose synthase (Susy) is the major enzyme of Sucrose (Suc) hydrolysis to UDP-glucose that could be used as a substrate for cellulose synthesis in cotton (*G. hirsutum*) fibers^50^. The combination of *GhSus* gene expression patterns and QTL analysis in our study further confirmed that *GhSus* family genes play important roles in fiber development.

Six SNPs (*GhSus1At*-A918G, *GhSus1At*-G1783C, *GhSus1Dt*-G751A, *GhSus1Dt*-G2709C, *GhSus3At*-T881A, and *GhSus6At*-C4655T) were found to be associated with seed-related traits (SI, PC, and OC) in addition to the SNPs associated with fiber-related traits. In a previous study, *Sus* was found to be abundant in transfer cells located at the innermost layer of the seed coat and in developing filial tissues, where *Sus* is involved in transfer cell wall ingrowth^3^ and endosperm cellularization^51^. Suppressing the expression of *Sus* in cotton seed coats leads to a fiberless phenotype^43^, and silencing its expression in the filial tissue results in stunted and unviable seeds and loss of transfer cells^43^,^51^. Xu^22^ overexpressed the potato *Sus* gene in cotton, and revealed that increased *Sus* activity in cotton could improve seed development, leading to enhanced seed weight and number. Overexpression of *Sus* could also enhance sink strength and sucrose supply by expanding leaves^22^. Jiang^42^ overexpressed *GhSusA1* in transgenic cotton and found this gene increased fiber length and strength. These findings provide an indication that it is valuable to increase fiber yield by enhancing fiber length and seed number. The new *GhSus* alleles found in our study may be used to increase fiber yield.

## Materials and Methods

### Plant materials

A set of 277 Upland cotton varieties possessing diverse fiber qualities, kindly provided by the Cotton Research Institute, Chinese Academy of Agricultural Sciences (CRI-CAAS), were subjected to EcoTILLING analysis. Of the 277 upland cotton accessions, four were from Australia, five from Africa, five from Europe, 50 from America, and 213 from Asia. Of the 213 accessions from Asia, two were from Pakistan, two from Vietnam, and 209 from China. The 209 accessions from China were from four geographic regions (the North region, Yellow River region, Yangtze River region, and northwest inland region). Detailed information on all accessions is shown in Supplementary Table S2.

### Phenotypic data

All 277 accessions were sown in three consecutive growing seasons (2007–2009) simultaneously at three main ecological cotton-growing areas of China: Nanjing in Jiangsu Province (31°14′~32°37′N, 118°22′~119°14′E; the average annual frost free period lasts 227–278 days, the average active accumulated temperature was 3500–5500 °C, annual rainfall was about 1000–1600 mm; Yangtze River valley cotton growing region), Anyang in Henan Province (35°12′~36°22′N, 113°37′~114°58′E; the average annual frost free period lasts 180–230 days, the average active accumulated temperature was 3800–4900 °C, annual rainfall was about 500–1000 mm; Yellow River valley cotton growing region) and Kuche in Xinjiang Uygur Autonomous Region (40°46′~42°35′N, 82°35′~84°17′E; the average annual frost free period lasts 170–230 days, the average active accumulated temperature was 3000–5400 °C, annual rainfall was about 15–380 mm; Northwest cotton growing region). A randomized complete block design with three replications was employed in the field trials, and each block was settled within a single-row plot at 5 m long and 0.7 m wide. Conventional field production management techniques that were adjusted to local practice were used.

Fiber samples were collected from bolls at the internal middle parts of plants, ginned by roller and sent to the Cotton Quality Supervision, Inspection and Testing Center of the Ministry of Agriculture, China to test fiber length (FL in mm), fiber strength (FS in cN/tex), fiber micronaire (FM), and fiber uniformity (FU in %) using an Uster HVI 900. Four yield traits, namely, lint percentage (LP in %), Seed index (g/100 seeds), boll weight (BW in g), and boll number per plant (BN), were also calculated. Seed kernel protein content (PC in %) and oil content (OC in %) were measured in 277 accessions. Phenotypic scores from field trials were shown in Supplementary Tables S6 and S7.

### DNA extraction

Equal quantities of fresh young leaves from each cotton variety were collected and immediately brought to the laboratory where total genomic DNA was extracted according to the protocol described by Paterson *et al.*^52^. DNA from all samples was quantified using a spectrophotometer and normalized to a concentration of 20~60 ng/μl.

### Designation and verification of subgenome-specific primers for duplicated gene amplification

To amplify target fragments of *GhSus* gene family sequences, gene-specific primers were designed according to the published DNA sequences of *GhSus* gene family members^29^,^53^ (Supplementary Tables S4 and S5). The expression patterns of *GhSus* family genes were analyzed from the public RNA-seq data^40^.

Since cultivated Upland cotton is allotetraploid, where many single nucleotide diversities exist between subgenomes, subgenome-specific primers were designed for *GhSus* gene family members to avoid errors caused by SNPs derived from duplicated genes in the A- and D-subgenomes. SNAPER software (http://ausubellab.mgh.harvard.edu/) was employed to design the subgenome- specific primers. All primers were evaluated for their subgenome specificity using PCR. Firstly, DNA templates from two diploid species, *G. raimondii* (D genome) and *G. herbaceum* var. *africanum* (A genome), and one tetraploid species, *G. hirsutum* acc. TM-1 (AD genome), were amplified. The PCR products were then subjected to polyacrylamide gel electrophoresis (PAGE) to verify the primer specificity. When the PCR products only existed in two species, *G. herbaceum* var. *africanum* and *G. hirsutum* acc. TM-1, but not in *G. raimondii*, the primers were considered to be At subgenome-specific primers. When the PCR products only existed in *G. raimondii* and *G. hirsutum* acc. TM-1, but not in *G. herbaceum* var. *africanum*, the primers were considered to be Dt-subgenome specific primers. After subgenome-specific primers were acquired, semi-nested PCR primers were designed to further improve the PCR specificity and increase the product concentration.

### PCR amplification and EcoTILLING assays

Celery juice extract (CEL I enzyme) was extracted from celery following the method described by Till *et al.*^54^. CEL I enzymatic activity was evaluated by the digestion of heteroduplexes formed by control substances C and G, which were both supplied in the Surveyor Mutation Discovery kit. The enzyme digestion conditions followed those described in the Surveyor Mutation Discovery kit protocol (Transgenomics).

To identify nucleotide diversities by EcoTILLING, gene- and subgenome- specific primers were designed based on the *GhSus* family gene sequences and used in a semi-nested PCR. To avoid base mismatch during PCR, an ExTaq (TAKARA, China) enzyme with high fidelity was used in three replicates. The first PCR was performed with gene- and subgenome- specific primer pairs. The PCR amplified products were then used as templates for the second semi-nested PCR reaction. Both steps of the PCR reaction used the same forward primer but distinct reverse primers (Supplementary Table S6).

After PCR, an EcoTILLING protocol was employed for SNP screening, as described by Kadaru *et al.*^55^ with some modifications. First, the semi-nested PCRs were performed, and then the PCR products of each cotton variety were mixed with that of TM-1 and incubated at 99 °C for denaturing 10 min, followed by annealing with 28 cycles of 20 sec at 72 °C with a decrement of 0.3 °C per cycle and then cooling to 42 °C with a decrement of 1 °C per cycle, before finally being cooled to 24 °C for 1 min. Finally, if SNPs existed between the cotton variety and TM-1, base-mismatched DNA heteroduplexes would be formed in the mixture of the two PCR products and could be digested specifically at mismatched base position by endonuclease, CEL I. The digestion of heteroduplexes generated several small-sized DNA bands that were visualized using PAGE, implying that SNPs existed between the cotton variety and TM-1 gene sequences. Otherwise, if no SNPs existed between the cotton variety and TM-1, heteroduplexes would not be formed and could not be digested by CEL I. No small-sized DNA fragments generated. Once a polymorphic band was identified, the corresponding DNA samples were amplified using gene- and subgenome- specific primers. The resulting PCR fragments were directly sequenced for each polymorphic site from at least three accessions to confirm that only two alleles segregated at any specific site.

### Nomenclature of SNP

Here, SNP was named as “gene + subgenome + dash + base in TM-1 + base position + base in the accession analyzed. For example, the SNP, *GhSus1Dt-G751A*, demonstrates that G base on the position of Dt subgenome in TM-1 is substituted by A base in the accession analyzed (Supplementary Table S8).

### DNA sequencing and statistical analysis

Once polymorphisms were found for a targeted region of the *GhSus* family genes by EcoTILLING, accessions that showed polymorphisms on the gel were randomly selected for sequencing by a commercial company (Genscript Biotech Co. China) to confirm the polymorphisms. ClustalX software was used to find the SNP sites in the obtained sequences. The SIFT (Sorting Intolerant from Tolerant) method^56^ was used to predict deleterious effects of SNPs to protein function. Haplotype diversity (HD) was analyzed using DnaSP v5.1^57^.

### Calculation of gene expression levels

Genomic (as reference) and RNA-seq data of upland cotton TM-1 was obtained from the NCBI SRA database (accession codes: PRJNA248163)^40^. RNA-seq reads were mapped to the TM-1 genome (Version 1.1) using Tophat (Version 2.0.8)^58^. To measure gene expression level in fiber and ovule, we calculated the expression of each gene using FPKM (Fragments per Kilobase of exon model per Million mapped reads) with Cufflinks (Version 2.1.1)^59^.

### Association analysis between SNPs and agronomic traits

A possible population structure was depicted using 258 simple sequence repeat (SSR) markers by STRUCTURE software version 2^60^. The length of the burn-in period and the number of Markov chain Monte Carlo replications after burn-in were all assigned at 100,000 with an admixture and allele frequencies correlated model. The hypothetical number of subpopulations (*k*) ranged from 1 to 10 was performed at five independent run iterations. The log probability of data from the STRUCTURE output was used to estimate the *k* according to the method described by Evanno *et al.*^38^. Based on the *k*, the population structure matrix (Q) was generated for further marker-trait association analysis.

The tentative association between nucleotide variations in the *Sus* gene family with agronomic traits was performed by an epistatic association mapping (EAM) approach proposed by Lü *et al.*^39^. The genetic model extended was as follows:


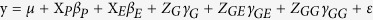


where ***y*** is the phenotypic value of agronomic traits, ***X***_*p*_ is the *Q* matrix for the population structure, *X*_*E*_, *Z*_*G*_, *Z*_*GE*_, and *Z*_*GG*_ are the design matrices of the environment effect, QTL effect, QTL-by-environment interaction effect, and QTL-by-QTL interaction effect, respectively, and *β*_*P*_, *β*_*E*_, *γ*_*G*_, *γ*_*GE*_, and *γ*_*GG*_ are the corresponding effects. Note that *γ*_*G*_ includes additive and dominant effects, *γ*_*G*_ includes additive-by-environment and dominant-by-environment effects, and *γ*_*GG*_ includes additive-by-additive, additive-by-dominant, dominant-by-additive and dominant-by-dominant effects. All the effects in the above model were estimated by empirical Bayes^61^. Results from Monte Carlo simulation studies showed that these estimates were unbiased^61^,^62^,^63^. The significance threshold of the logarithm of odds (LOD) score was set at 3, which is equivalent to the significance level of 0.0002^63^.

## Additional Information

**How to cite this article**: Zeng, Y.-D. *et al.* EcoTILLING revealed SNPs in *GhSus* genes that are associated with fiber- and seed-related traits in upland cotton. *Sci. Rep.* **6**, 29250; doi: 10.1038/srep29250 (2016).

## Supplementary Material

Supplementary Information

## Figures and Tables

**Table 1:** List of nucleotide polymorphisms in candidate genes.

Gene	Nucleotide polymorphism^a^	Ratio^b^	Variation of amino acid^c^	SIFT^d^
*GhSus1At*	A918G	0.612	No	
	G1783C	0.342	No	
	A2940T	0.338	T678=	
*GhSus1Dt*	T650C	0.089	No	
	G751A	0.089	V153I	0
	G2709C	0.107	E601Q	0.12
*GhSus3At*	T881A	0.072	No	
	T1320C	0.072	No	
	G1327A	0.072	No	
	T2294C	0.018	L504S	0
	A2472C	0.018	No	
*GhSus4Dt*	A1886T	0.025	No	
	T2167C	0.025	E388=	
*GhSus5Dt*	G491T	0.029	L108F	1
	C2648G	0.025	P660A	1
*GhSus6At*	G3143T	0.159	No	
	C4655T	0.209	No	
	G4697A	0.209	W755=	
	G5005A	0.209	No	
*GhSus7Dt*	T192C	0.755	T64=	
	C404T	0.755	D109=	
	T407C	0.755	E110=	
*GhSus8Dt*	C577T	0.09	No	
	T1334C	0.069	G298=	

^a^The first letter indicates bp at this site in TM-1 sequence, followed by the position of the SNP in the TM-1 sequence, and then the nucleotide that is the rare variant at this site.

^b^Ratio was calculated by dividing the number of similar nucleotide changes identified on the EcoTILLING gel by 277 upland cotton accessions.

^c^The first letter indicates the common amino acid at this site, followed by the position of the SNP within the predicated protein sequence and the amino acid change induced by the variant nucleotide polymorphism; “=” means no change in amino acid encoded by that codon (synonymous variation); “No” means that SNP was in intron.

^d^A non-synonymous SNP is predicted to be damaging to the encoded protein if the SIFT score is <0.05 (bold).

**Table 2:** Gene length, number of SNPs, number of haplotypes and haplotype diversity (HD) for the *GhSus* family genes.

Gene	Length (bp)	Number of SNP	Number of Haplotype	HD
*GhSus1At*	3650	3	8	0.574
*GhSus1Dt*	3648	3	4	0.290
*GhSus3At*	3491	5	4	0.143
*GhSus4Dt*	4790	2	2	0.049
*GhSus5Dt*	3167	2	4	0.064
*GhSus6At*	5176	4	4	0.386
*GhSus7Dt*	3428	3	2	0.372
*GhSus8Dt*	3762	2	4	0.201

**Table 3:** Association analysis with fiber- or seed- realted traits of SNPs in the *GhSus* family genes.

Trait	QTN	LOD	r^2^(%)	F	*P*	Favorable allele	
						Alleles	Effect
LP	*GhSus1At*-A2940T	16.59	3.43	15.16	1.01E-04^**^	*GhSus1At-*2940T	0.95%
BW	*GhSus1At*-G1783C	14.60	3.56	12.71	3.71E-04^**^	*GhSus1At-*1783C	0.18 g
	*GhSus3At*-T881A	15.30	1.76	77.09	3.02E-18^**^	*GhSus3At-*881A	0.23 g
	*GhSus1At*-A2940T × *GhSus1Dt*-G2709C	10.64	2.03	21.42	6.03E-10^**^	*GhSus1At*-2940T × *GhSus1Dt*-2709C	0.14 g
	*GhSus6At*-C4655T × *GhSus7Dt*-T192C	8.44	0.76	33.00	1.04E-08^**^	*GhSus6At*-4655T × *GhSus7Dt*-192C	0.08 g
SI	*GhSus3At*-T881A	10.82	1.32	65.96	7.25E-16^**^	*GhSus3At-*881A	0.40 g
	*GhSus1At*-A918G × *GhSus1Dt*-G2709C	10.98	2.10	10.83	1.01E-03^*^	*GhSus1At*-918G × *GhSus1Dt*-2709C	0.26 g
	*GhSus1At*-G1783C × *GhSus1Dt*-G2709C	17.73	2.27	28.49	5.91E-13^**^	*GhSus1At*-1783C × *GhSus1Dt*-2709C	0.29 g
	*GhSus1Dt*-G751A × *GhSus1Dt*-G2709C	6.58	0.89	40.02	2.98E-10^**^	*GhSus1Dt*-751A × *GhSus1Dt*-2709C	0.25 g
FL	*GhSus1At*-A918G × *GhSus5Dt*-C2648G	16.53	3.48	14.62	4.90E-07^**^	*GhSus1At*-918G × *GhSus5Dt*-2648G	0.42 cm
	*GhSus1At*-A918G × *GhSus6At*-C4655T	7.98	1.09	31.51	2.21E-08^**^	*GhSus1At*-918A × *GhSus6At*-4655C	0.23 cm
	*GhSus1Dt*-G751A × *GhSus3At*-T881A	11.97	1.50	22.29	2.57E-10^**^	*GhSus1Dt*-751A × *GhSus3At*-881A	0.39 cm
FS	*GhSus3At*-T881A × *GhSus7Dt*-T192C	4.81	0.43	14.22	7.24E-07^**^	*GhSus3At*-881A × *GhSus7Dt*-192C	0.19 cN/tex
FM	*GhSus1Dt*-G751A × *GhSus8Dt*-T1334C	5.15	0.89	14.56	5.17E-07^**^	*GhSus1Dt*-751A × *GhSus8Dt*-1334C	0.07
	*GhSus1Dt*-G2709C × *GhSus8Dt*-C577T	5.11	1.27	6.72	1.23E-03^*^	*GhSus1Dt*-2709G × *GhSus8Dt*-577C	0.08
PC	*GhSus1At*-A918G × *GhSus1Dt*-G2709C	9.57	2.53	11.11	1.62E-05^**^	*GhSus1At*-918A × *GhSus1Dt*-2709G	0.49%
OC	*GhSus6At*-C4655T	3.67	1.96	15.21	1.04E-04^**^	*GhSus6At*-4655C	0.54%

* and **at the 0.05 and 0.01 levels of significance on the genome, respectively. LP, lint percentage (LP); BW, boll weight; SI, seed index; FL, fiber length; FM, fiber micronaire; FU, fiber uniformity; FS, fiber strength; PC, protein content; OC, oil content. r^2^ is the phenotypic variance explained by each QTN.
