# Supplementary material for: EcoTILLING revealed SNPs in GhSus genes that are associated with fiber- and seed-related traits in upland cotton
Source: Sci Rep. 2016 Jul 7;6:29250. doi: 10.1038/srep29250 (PMC4935865; doi:10.1038/srep29250)
Supplement: Supplementary Information [file srep29250-s1.doc]

**EcoTILLING revealed SNPs in *GhSus* genes that are** **associated with fiber- and seed-related traits in upland cotton**

Yan-Da Zeng^1^, Jun-Ling Sun^2^, Su-Hong Bu^1^, Kang-Sheng Deng^1^, Tao Tao^1^, Yuan-Ming Zhang^3^, Tian-Zhen Zhang^1^, Xiong-Ming Du^2^*, and Bao-Liang Zhou^1^*

**
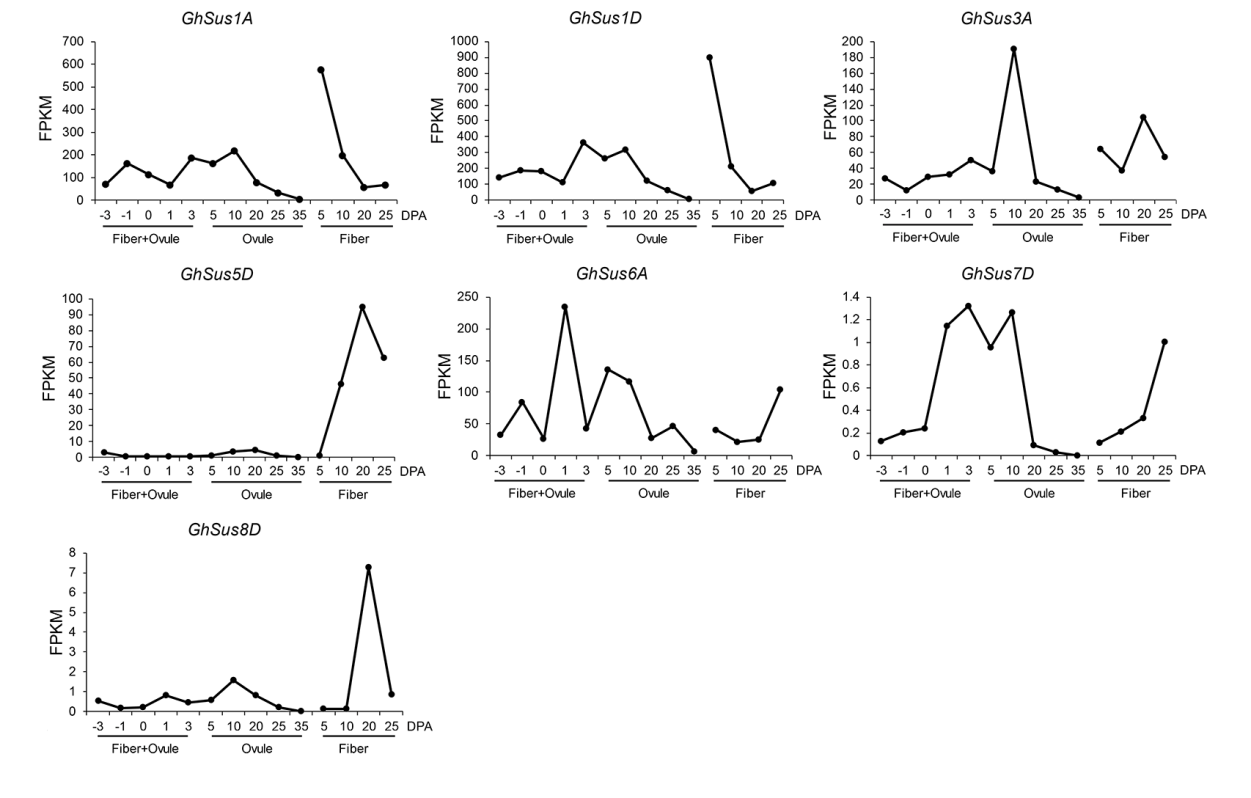
**

**Figure S1**. **Expression pattern of *GhSus* family genes in ovules and fibers of upland cotton (TM-1) during fiber development**. FPKM means Fragments Per Kilobase of exon model per Million mapped reads. DPA means days post-anthesis.

**Table S1 Gene haplotypes were identified.**

| Gene | | | Base in haplotype | | | | | Frequency |
| --- | --- | --- | --- | --- | --- | --- | --- | --- |
| *GhSus1At*  3650bp | SNP position | | 917 | 1783 | 2940 |  | | |
|  | Haplotype | H1 | A | G | A |  | | 0.303 |
|  |  | H2 | G | G | A |  |  | 0.347 |
|  |  | H3 | A | C | A |  |  | 0.004 |
|  |  | H4 | A | G | T |  |  | 0.004 |
|  |  | H5 | G | C | A |  |  | 0.007 |
|  |  | H6 | A | C | T |  |  | 0.079 |
|  |  | H7 | G | G | T |  |  | 0.011 |
|  |  | H8 | G | C | T |  |  | 0.245 |
| *GhSus1Dt*  3648bp | SNP position | | 650 | 751 | 2709 |  | | |
|  | Haplotype | H1 | T | G | G |  | | 0.838 |
|  |  | H2 | C | A | G |  |  | 0.058 |
|  |  | H3 | T | G | C |  |  | 0.072 |
|  |  | H4 | C | A | C |  |  | 0.032 |
| *GhSus3At*  3491bp | SNP position | | 881 | 1320 | 1327 | 2294 | 2472 |  |
|  | Haplotype | H1 | T | T | G | T | A | 0.924 |
|  |  | H2 | A | C | A | T | A | 0.058 |
|  |  | H3 | T | T | G | C | C | 0.004 |
|  |  | H4 | A | C | A | C | C | 0.014 |
| *GhSus4Dt*  4790bp | SNP position | | 1886 | 2167 |  | | | |
|  | Haplotype | H1 | A | T |  | | | 0.975 |
|  |  | H2 | T | C |  |  |  | 0.025 |
| *GhSus5Dt*  3167bp | SNP position | | 491 | 2648 |  |  |  |  |
|  | Haplotype | H1 | G | C |  |  |  | 0.968 |
|  |  | H2 | T | C |  |  |  | 0.007 |
|  |  | H3 | G | G |  |  |  | 0.004 |
|  |  | H4 | T | G |  |  |  | 0.022 |
| *GhSus6At*  5176bp | SNP position | | 3143 | 4655 | 4697 | 5005 |  | |
|  | Haplotype | H1 | G | C | G | G |  | 0.769 |
|  |  | H2 | T | C | G | G |  | 0.022 |
|  |  | H3 | G | T | A | A |  | 0.072 |
|  |  | H4 | T | T | A | A |  | 0.137 |
| *GhSus7Dt*  3428bp | SNP position | | 192 | 404 | 407 |  | | |
|  | Haplotype | H1 | T | C | T |  | | 0.245 |
|  |  | H2 | C | T | C |  |  | 0.755 |
| *GhSus8Dt*  3762bp | SNP position | | 577 | 1334 |  | | | |
|  | Haplotype | H1 | C | T |  | | | 0.892 |
|  |  | H2 | T | T |  |  |  | 0.040 |
|  |  | H3 | C | C |  |  |  | 0.018 |
|  |  | H4 | T | C |  |  |  | 0.051 |

**Table S2 277 upland cotton accessions and their geographical origin**

| Serial No. | Germplasm Name | Geographical Origin | Q value* |
| --- | --- | --- | --- |
| 1 | 70-29-5 | Yangtze River regions of China | 0.855 |
| 2 | BaZhou5628 | Northwest regions of China | 0.986 |
| 3 | Bao6716 | Yellow River region of China | 0.389 |
| 4 | ChangRong67-12 | Yangtze River regions of China | 0.409 |
| 5 | Hu749513 | Yellow River region of China | 0.423 |
| 6 | Jin444 | North regions of China | 0.332 |
| 7 | KuCheT94-1 | Northwest regions of China | 0.915 |
| 8 | Su7036YuanYuan | Yangtze River regions of China | 0.403 |
| 9 | WanJiu828 | Yangtze River regions of China | 0.707 |
| 10 | E408 | Yangtze River regions of China | 0.142 |
| 11 | Han8959 | Yellow River region of China | 0.850 |
| 12 | HuaZhong91-0102 | Yangtze River regions of China | 0.199 |
| 13 | Ji91-31 | Yellow River region of China | 0.269 |
| 14 | KuiTun80-2056W(XinLuZao-3Hao) | Northwest regions of China | 0.748 |
| 15 | MianYang73-39 | Yangtze River regions of China | 0.477 |
| 16 | QingKang1Hao | Yangtze River regions of China | 0.519 |
| 17 | Sha24-3 | Northwest regions of China | 0.421 |
| 18 | YangFen31Hao | Yellow River region of China | 0.088 |
| 19 | ZaoShuChangRong7 | Yellow River region of China | 0.938 |
| 20 | YaHuang9103 | Yellow River region of China | 0.221 |
| 21 | ChuanGaoYiFen58 | Yangtze River regions of China | 0.100 |
| 22 | KuiTunXi96-167 | Northwest regions of China | 0.418 |
| 23 | ShiYuan638 | Yellow River region of China | 0.004 |
| 24 | SuXu138 (Zaoshu) | Yangtze River regions of China | 0.154 |
| 25 | ZhongARR40682 | Yellow River region of China | 0.009 |
| 26 | S-050019 | Northwest regions of China | 0.901 |
| 27 | JiangSuDaTao | Yangtze River regions of China | 0.375 |
| 28 | NongJiu | Yellow River region of China | 0.995 |
| 29 | NangFenDaTao | Yellow River region of China | 0.418 |
| 30 | XiaoXianDaLing | Yangtze River regions of China | 0.099 |
| 31 | Acala (DaLing)B | America | 0.926 |
| 32 | DaLingFuZiMian | Yangtze River regions of China | 0.383 |
| 33 | DaLingMian | Yangtze River regions of China | 0.754 |
| 34 | MeiFuDaLing | Yellow River region of China | 0.471 |
| 35 | QiFengDaLing | Yangtze River regions of China | 0.039 |
| 36 | XinDaLing | Yellow River region of China | 0.587 |
| 37 | XinZhouDaLing | Yangtze River regions of China | 0.145 |
| 38 | ZhongARR40681 | Yellow River region of China | 0.007 |
| 39 | DaTaoMian | Yellow River region of China | 0.636 |
| 40 | GP70 | America | 0.189 |
| 41 | LuMian11Hao (3389) | Yellow River region of China | 0.186 |
| 42 | ShanNong3Hao | Yellow River region of China | 0.212 |
| 43 | Si168 | Yangtze River regions of China | 0.383 |
| 44 | XuZhou244 | Yangtze River regions of China | 0.105 |
| 45 | ZhengKang01-505 | Yellow River region of China | 0.027 |
| 46 | Zhong2108 | Yellow River region of China | 0.291 |
| 47 | ZhongJi926 | Yellow River region of China | 0.269 |
| 48 | CZA(70)33 | Yellow River region of China | 0.482 |
| 49 | DES926 | America | 0.420 |
| 50 | GP93 | America | 0.276 |
| 51 | Miscot7803-52 | America | 0.318 |
| 52 | N73DeltapineNGF | America | 0.059 |
| 53 | Qik | America | 0.469 |
| 54 | MeiG-82 | America | 0.688 |
| 55 | MeiG-84 | America | 0.402 |
| 56 | ZhongMianSuo32 | Yellow River region of China | 0.303 |
| 57 | ZhongAR40772 | Yellow River region of China | 0.210 |
| 58 | JiA-7-8 (33Xi) | Yellow River region of China | 0.986 |
| 59 | JCG59 | Yangtze River regions of China | 0.136 |
| 60 | KangHuangWei164 | Yellow River region of China | 0.314 |
| 61 | Shan2747 | Yellow River region of China | 0.570 |
| 62 | Shan2800 | Yellow River region of China | 0.461 |
| 63 | Shan960329-2Yuan3 | Yellow River region of China | 0.405 |
| 64 | Yun92Kang124 | Yellow River region of China | 0.635 |
| 65 | Zhong870203 | Yellow River region of China | 0.631 |
| 66 | Zhong961716 | Yellow River region of China | 0.994 |
| 67 | ZhongZhiBD13 | Yellow River region of China | 0.310 |
| 68 | ZhongZhiBD27 | Yellow River region of China | 0.115 |
| 69 | ZhongZi640 | Yellow River region of China | 0.052 |
| 70 | Liao4835 | North regions of China | 0.389 |
| 71 | Liao4853 | North regions of China | 0.567 |
| 72 | LiaoMian16 | North regions of China | 0.347 |
| 73 | LiaoMian17 | North regions of China | 0.650 |
| 74 | LiaoMian18 | North regions of China | 0.473 |
| 75 | YongJi1Hao | Yellow River region of China | 0.089 |
| 76 | YongJi2Hao | Yellow River region of China | 0.024 |
| 77 | ZhongYuan911 | Yellow River region of China | 0.019 |
| 78 | ZhongYuan9114 | Yellow River region of China | 0.034 |
| 79 | ZhongYuan9115 | Yellow River region of China | 0.435 |
| 80 | ZhongYuan9116 | Yellow River region of China | 0.425 |
| 81 | ZhongYuanHAS-1 | Yellow River region of China | 0.056 |
| 82 | ChangKangMian | Yangtze River regions of China | 0.038 |
| 83 | Zhong521 | Yellow River region of China | 0.013 |
| 84 | ZhongYuan9112 | Yellow River region of China | 0.170 |
| 85 | 86-1 (72-100) | Yellow River region of China | 0.497 |
| 86 | ZiSeMeiMian | Yellow River region of China | 0.988 |
| 87 | LiaoMian-5Hao | North regions of China | 0.351 |
| 88 | Arcot436 | America | 0.415 |
| 89 | Coker139 | America | 0.473 |
| 90 | GP137 | America | 0.176 |
| 91 | GP138 | America | 0.448 |
| 92 | GP83 | America | 0.263 |
| 93 | Zhong89-1 | Yellow River region of China | 0.019 |
| 94 | Miscot78-27 | America | 0.290 |
| 95 | USSR8911 | America | 0.994 |
| 96 | AC239 | America | 0.875 |
| 97 | AoSiv2 | America | 0.281 |
| 98 | Mei8123 | America | 0.009 |
| 99 | Acala SJ-4 | America | 0.028 |
| 100 | ZhongMianSuo17Hao (Zhong117) | Yellow River region of China | 0.058 |
| 101 | ZhongMianSuo19Hao (Zhong7886) | Yellow River region of China | 0.059 |
| 102 | HG-BR-8 | Europe | 0.262 |
| 103 | LAPAR45 | America | 0.231 |
| 104 | FeizhouE40 | Africa | 0.446 |
| 105 | L142-9 | Africa | 0.405 |
| 106 | Ao152 | Australia | 0.071 |
| 107 | AoC | Australia | 0.277 |
| 108 | JiA-1-7 (33Xi) | Yellow River region of China | 0.635 |
| 109 | LuNong9648 | Yellow River region of China | 0.282 |
| 110 | MeiF-18 | America | 0.789 |
| 111 | MeiF-19 | America | 0.262 |
| 112 | SuQ1 | Yangtze River regions of China | 0.519 |
| 113 | SuTKH-1 | Yangtze River regions of China | 0.059 |
| 114 | SuYuan7235 | Yangtze River regions of China | 0.822 |
| 115 | ShenMian5Hao | Yellow River region of China | 0.349 |
| 116 | Zhong4612YaH | Yellow River region of China | 0.017 |
| 117 | ZhongR03 | Yellow River region of China | 0.218 |
| 118 | UA887(You) | America | 0.302 |
| 119 | DP33B | America | 0.017 |
| 120 | GK20 | Yellow River region of China | 0.379 |
| 121 | ST474 | America | 0.007 |
| 122 | ZhongMianSuo35 | Yellow River region of China | 0.358 |
| 123 | SGKShiXuan321 | Yellow River region of China | 0.009 |
| 124 | SiMian2Hao | Yangtze River regions of China | 0.194 |
| 125 | USSR91Xi | Europe | 0.851 |
| 126 | GP67 | America | 0.279 |
| 127 | ZhongZi9102 | Yellow River region of China | 0.602 |
| 128 | ZhongZi9103 | Yellow River region of China | 0.412 |
| 129 | USSR116Xi | Europe | 0.997 |
| 130 | Ji91-12 | Yellow River region of China | 0.609 |
| 131 | Ji85-3 | Yellow River region of China | 0.213 |
| 132 | KuCheT94-4 | Northwest regions of China | 0.985 |
| 133 | FB20 | America | 0.005 |
| 134 | Liao96-23-30 | North regions of China | 0.610 |
| 135 | Yun93Kang354 | Yellow River region of China | 0.666 |
| 136 | Yun93Kang393 | Yellow River region of China | 0.681 |
| 137 | HanDan109 | Yellow River region of China | 0.025 |
| 138 | Liao823-834-23 | North regions of China | 0.735 |
| 139 | KuChe96518 | Northwest regions of China | 0.872 |
| 140 | Bu3363 | America | 0.993 |
| 141 | Ji91-33 | Yellow River region of China | 0.889 |
| 142 | DP2156 | America | 0.478 |
| 143 | r4136 | another Asia regions | 0.977 |
| 144 | Cook 310-5110 | Australia | 0.440 |
| 145 | JiZi123 (Jimian25) | Yellow River region of China | 0.347 |
| 146 | XuZhou261 | Yangtze River regions of China | 0.387 |
| 147 | ZhongYuanHST1 | Yellow River region of China | 0.841 |
| 148 | JiYuan55 (91Han14) | Yellow River region of China | 0.009 |
| 149 | RTBaiXu | Yellow River region of China | 0.392 |
| 150 | Upland | America | 0.702 |
| 151 | Lu458 | Yellow River region of China | 0.177 |
| 152 | WangJiangChangRongMian | Yangtze River regions of China | 0.713 |
| 153 | EKangMian9Hao | Yangtze River regions of China | 0.178 |
| 154 | Ji668 | Yellow River region of China | 0.212 |
| 155 | HanDan333 | Yellow River region of China | 0.036 |
| 156 | LuMianYan21(Lu1138) | Yellow River region of China | 0.004 |
| 157 | DaZeMian | Yellow River region of China | 0.378 |
| 158 | QinYuan4Hao (Qinyuan91406) | Yellow River region of China | 0.191 |
| 159 | HanDanChangRong | Yellow River region of China | 0.467 |
| 160 | JiHaiLu6 (91Han6) | Yellow River region of China | 0.668 |
| 161 | PD6186 | America | 0.770 |
| 162 | 0102X-10-1 | Northwest regions of China | 0.883 |
| 163 | XuShiDaTao | Yangtze River regions of China | 0.616 |
| 164 | ChengDingDaLing | Yellow River region of China | 0.219 |
| 165 | XinYan96-48 | Yellow River region of China | 0.490 |
| 166 | ZhongMianSuo16 | Yellow River region of China | 0.743 |
| 167 | LineF | America | 0.895 |
| 168 | ZhongMianSuo41 (SGK9708) | Yellow River region of China | 0.009 |
| 169 | ZhongMianSuo12 | Yellow River region of China | 0.618 |
| 170 | LiaoMian19 | North regions of China | 0.324 |
| 171 | AcalaSJ-1-9 | America | 0.677 |
| 172 | ZhongMianSuo49 | Yellow River region of China | 0.181 |
| 173 | ZhongMianSuo50 | Yellow River region of China | 0.352 |
| 174 | Ji91-18 | Yellow River region of China | 0.017 |
| 175 | ZhongG5 | Yellow River region of China | 0.153 |
| 176 | 99633 | Yellow River region of China | 0.304 |
| 177 | SuYuan04-162 | Yangtze River regions of China | 0.540 |
| 178 | Zhong2201 | Yellow River region of China | 0.375 |
| 179 | HongTao | Yellow River region of China | 0.450 |
| 180 | LangHuangF10 (ZongXu) | Yellow River region of China | 0.054 |
| 181 | SuMian9108 | Yangtze River regions of China | 0.431 |
| 182 | JiYuan12-13 | Yellow River region of China | 0.359 |
| 183 | ZhongZi4480 | Yellow River region of China | 0.953 |
| 184 | ZhongZi9196 (Jiguang) | Yellow River region of China | 0.938 |
| 185 | Jing55263 | Yangtze River regions of China | 0.223 |
| 186 | Ejing55173 | Yangtze River regions of China | 0.223 |
| 187 | Jing55168 | Yangtze River regions of China | 0.198 |
| 188 | YuMian2067 | Yellow River region of China | 0.170 |
| 189 | MSCO-12 | Yellow River region of China | 0.703 |
| 190 | M11 | another Asia regions | 0.861 |
| 191 | Arcot438 | America | 0.696 |
| 192 | Ji91-22 | Yellow River region of China | 0.994 |
| 193 | BPA68 | Africa | 0.483 |
| 194 | ZhongARR40683 | Yellow River region of China | 0.145 |
| 195 | NaShangQuDaHua | Yangtze River regions of China | 0.874 |
| 196 | Acala SJ-5 | America | 0.218 |
| 197 | XiangMian2Hao | Yangtze River regions of China | 0.263 |
| 198 | GK99-1 | Yellow River region of China | 0.200 |
| 199 | SuMian2Hao (Xuzhou553) | Yangtze River regions of China | 0.288 |
| 200 | QinLi514 | Yellow River region of China | 0.539 |
| 201 | GP95 | America | 0.331 |
| 202 | Zhong1276 | Yellow River region of China | 0.344 |
| 203 | Shan3184 | Yellow River region of China | 0.198 |
| 204 | zhongＲ40773 | Yellow River region of China | 0.487 |
| 205 | Acala SJ-1 | America | 0.976 |
| 206 | Arcot-1 | America | 0.014 |
| 207 | Tu83-161 | Northwest regions of China | 0.566 |
| 208 | AoL23/757 | Australia | 0.227 |
| 209 | YuMian19 | Yellow River region of China | 0.113 |
| 210 | XingTai79-11 | Yellow River region of China | 0.008 |
| 211 | DP410B | America | 0.028 |
| 212 | M-8124-1159 | America | 0.327 |
| 213 | J02-508 | Yangtze River regions of China | 0.559 |
| 214 | zhengzhouchangrongmian | Yellow River region of China | 0.008 |
| 215 | Acala1517-2 | America | 0.216 |
| 216 | ZhaDe3Hao | Africa | 0.436 |
| 217 | Han8901 | Yellow River region of China | 0.367 |
| 218 | Arcot402bne | America | 0.693 |
| 219 | JinKang157 | Yellow River region of China | 0.775 |
| 220 | SiChang2Xi | Yangtze River regions of China | 0.283 |
| 221 | Acala 927 | America | 0.856 |
| 222 | HuaiMian4Hao | Yangtze River regions of China | 0.845 |
| 223 | Yun3060 | Yellow River region of China | 0.486 |
| 224 | SuYuan04-129 | Yangtze River regions of China | 0.321 |
| 225 | Lu21 | Yellow River region of China | 0.004 |
| 226 | Zhong31-204 | Yellow River region of China | 0.437 |
| 227 | LinQing201 | Yellow River region of China | 0.084 |
| 228 | MM-2 | America | 0.984 |
| 229 | 353DaLingDi3Xi | Yellow River region of China | 0.875 |
| 230 | MSCO-11 | Yellow River region of China | 0.721 |
| 231 | Bao6722 | Yellow River region of China | 0.628 |
| 232 | YuMian９Hao (Yuzao1109) | Yellow River region of China | 0.010 |
| 233 | J02-247 | Yangtze River regions of China | 0.493 |
| 234 | Zhong07 | Yellow River region of China | 0.254 |
| 235 | 601ChangRongMian | Yangtze River regions of China | 0.272 |
| 236 | MeiD#1 | America | 0.704 |
| 237 | r-3149 | another Asia regions | 0.948 |
| 238 | Zhong932906 | Yellow River region of China | 0.425 |
| 239 | GaoYiMian | Yangtze River regions of China | 0.039 |
| 240 | JiMian12Hao (Handan177) | Yellow River region of China | 0.009 |
| 241 | PAR-51 | another Asia regions | 0.789 |
| 242 | Jin90Kang282 | Yellow River region of China | 0.688 |
| 243 | Han109 | Yellow River region of China | 0.015 |
| 244 | Jimian11 (CK) | Yellow River region of China | 0.359 |
| 245 | NeiHuangDaZi | Yellow River region of China | 0.554 |
| 246 | BaZhou7416 | Northwest regions of China | 0.937 |
| 247 | BELLSIRO | America | 0.379 |
| 248 | ZhaoYang70Hao | North regions of China | 0.501 |
| 249 | HanDan568 | Yellow River region of China | 0.265 |
| 250 | N74-250 | America | 0.247 |
| 251 | Liao96-63-70 | North regions of China | 0.678 |
| 252 | Han8944 | Yellow River region of China | 0.426 |
| 253 | Yu284 | Yellow River region of China | 0.154 |
| 254 | Zhong85271 | Yellow River region of China | 0.198 |
| 255 | AnJiYan6Hao | Europe | 0.901 |
| 256 | JiFeng197 | Yellow River region of China | 0.155 |
| 257 | Zhong12 | Yellow River region of China | 0.253 |
| 258 | Mei28114-313 | America | 0.010 |
| 259 | USSR21Xi (91-133) | Europe | 0.358 |
| 260 | KangSanXingDaTao | Yangtze River regions of China | 0.539 |
| 261 | YuMian2Hao (YuWu302) | Yellow River region of China | 0.298 |
| 262 | ShanNong6Hao | Yellow River region of China | 0.008 |
| 263 | S-050031 | Northwest regions of China | 0.784 |
| 264 | ChangRongMuZiMian | Yellow River region of China | 0.166 |
| 265 | XinXiang89S-210 | Yellow River region of China | 0.009 |
| 266 | XuZhouBanBanMian | Yangtze River regions of China | 0.045 |
| 267 | ZhaDeMian | Africa | 0.100 |
| 268 | Zhong5913-2 | Yellow River region of China | 0.254 |
| 269 | KuCheT94-6 | Northwest regions of China | 0.936 |
| 270 | DaLingMian69Hao | Yangtze River regions of China | 0.544 |
| 271 | Liao61107 | North regions of China | 0.918 |
| 272 | JCG94 | Yangtze River regions of China | 0.503 |
| 273 | KuChe93551 | Northwest regions of China | 0.838 |
| 274 | Tu188 | Northwest regions of China | 0.262 |
| 275 | JiHan2HaoXuanXi | Yellow River region of China | 0.368 |
| 276 | YanCheng1115 | Yangtze River regions of China | 0.005 |
| 277 | Arcot-1(yuan) | America | 0.128 |

Upland cotton accessions were assigned to subpopulation 1 (SP1) if the membership value (Q value) was > 0.500, or to subpopulation 2 (SP2) otherwise.

**Table S3 Interaction of haplotypes between genes for traits in ANOVA**

| **Trait** | **Source** | **DF** | **SS** | **Mean Square** | **F** | **P-value** |
| --- | --- | --- | --- | --- | --- | --- |
| **boll weight (BW)** | ENV | 8 | 30.82 | 3.85 | 7.65 | 2.96E-10 |
|  | *GhSus1A* | 7 | 34.76 | 4.97 | 9.87 | 2.61E-12 |
|  | *GhSus1A*×ENV | 56 | 21.93 | 0.39 | 0.78 | 0.89 |
|  | *GhSus1D* | 3 | 18.77 | 6.26 | 12.43 | 4.17E-08 |
|  | *GhSus1D*×ENV | 24 | 8.41 | 0.35 | 0.7 | 0.87 |
|  | ***GhSus1A***×***GhSus1D*** | **8** | **75.10** | **9.39** | **18.66** | **5.66E-28** |
|  | Error | 7260 | 3653.40 | 0.50 |  |  |
| **seed index (SI)** | ENV | 8 | 163.78 | 20.47 | 8.95 | 2.77E-12 |
|  | *GhSus1A* | 7 | 303.23 | 43.32 | 18.93 | 3.27E-25 |
|  | *GhSus1A×*ENV | 56 | 63.26 | 1.13 | 0.49 | 1.00 |
|  | *GhSus1D* | 3 | 121.17 | 40.39 | 17.65 | 2.06E-11 |
|  | *GhSus1D×*ENV | 24 | 32.45 | 1.35 | 0.59 | 0.94 |
|  | ***GhSus1A×GhSus1D*** | **8** | **918.78** | **114.85** | **50.18** | **1.56E-79** |
|  | Error | 7337 | 16791.73 | 2.29 |  |  |
| **protein content (PC)** | ENV | 2 | 84.01 | 42.00 | 5.18 | 0.01 |
|  | *GhSus1A* | 7 | 244.65 | 34.95 | 4.31 | 9.71E-05 |
|  | *GhSus1A×*ENV | 14 | 17.91 | 1.28 | 0.16 | 1.00 |
|  | *GhSus1D* | 3 | 40.28 | 13.43 | 1.66 | 0.17 |
|  | *GhSus1D×*ENV | 6 | 17.84 | 2.97 | 0.37 | 0.90 |
|  | ***GhSus1A×GhSus1D*** | **6** | **465.96** | **58.24** | **7.18** | **2.16E-09** |
|  | Error | 1621 | 13140.82 | 8.11 |  |  |
| **fiber length (FL)** | ENV | 8 | 55.16 | 6.90 | 2.49 | 0.01 |
|  | *GhSus1A* | 7 | 180.77 | 25.82 | 9.34 | 1.44E-11 |
|  | *GhSus1A×*ENV | 56 | 77.72 | 1.39 | 0.50 | 1.00 |
|  | *GhSus5D* | 3 | 79.24 | 26.41 | 9.55 | 2.72E-06 |
|  | *GhSus5D×*ENV | 24 | 27.19 | 1.13 | 0.41 | 1.00 |
|  | ***GhSus1A×GhSus5D*** | **2** | **85.12** | **42.56** | **15.39** | **2.14E-07** |
|  | Error | 7184 | 19866.88 | 2.77 |  |  |
| **fiber length (FL)** | ENV | 8 | 136.83 | 17.10 | 6.49 | 1.85E-8 |
|  | *GhSus1A* | 7 | 452.17 | 64.60 | 24.53 | 2.87E-33 |
|  | *GhSus1A×*ENV | 56 | 79.40 | 1.42 | 0.54 | 1.00 |
|  | *GhSus6A* | 3 | 411.70 | 137.23 | 52.11 | 2.61E-33 |
|  | *GhSus6A×*ENV | 24 | 26.35 | 1.10 | 0.42 | 1.00 |
|  | ***GhSus1A×GhSus6A*** | **8** | **615.47** | **76.93** | **29.21** | **2.87E-45** |
|  | Error | 7178 | 18904.62 | 2.63 |  |  |
| **boll weight (BW)** | ENV | 8 | 108.34 | 13.54 | 26.10 | 3.54E-40 |
|  | *GhSus7D* | 1 | 6.61 | 6.61 | 12.73 | 3.61E-04 |
|  | *GhSus7D×*ENV | 8 | 2.45 | 0.31 | 0.59 | 0.79 |
|  | *GhSus6A* | 3 | 12.40 | 4.13 | 7.96 | 2.68E-05 |
|  | *GhSus6A×*ENV | 24 | 12.76 | 0.53 | 1.02 | 0.43 |
|  | ***GhSus7D×GhSus6A*** | **3** | **99.37** | **33.12** | **63.84** | **9.76E-41** |
|  | Error | 7319 | 3797.76 | 0.52 |  |  |
| **fiber micronaire (FM)** | ENV | 8 | 90.16 | 11.27 | 43.85 | 3.25E-69 |
|  | *GhSus1D* | 3 | 8.01 | 2.67 | 10.39 | 8.06E-07 |
|  | *GhSus1D×*ENV | 24 | 2.22 | 0.09 | 0.36 | 1.00 |
|  | *GhSus8D* | 3 | 3.67 | 1.22 | 4.76 | 2.56E-03 |
|  | *GhSus8D×*ENV | 24 | 6.51 | 0.27 | 1.06 | 0.39 |
|  | ***GhSus1D×GhSus8D*** | **8** | **18.36** | **2.29** | **8.93** | **2.95E-12** |
|  | Error | 7213 | 1853.74 | 0.26 |  |  |
| **fiber length (FL)** | ENV | 8 | 96.88 | 12.11 | 4.45 | 2.15E-05 |
|  | *GhSus1D* | 3 | 122.70 | 40.90 | 15.02 | 9.56E-10 |
|  | *GhSus1D×*ENV | 24 | 59.76 | 2.49 | 0.91 | 0.58 |
|  | *GhSus3A* | 3 | 192.16 | 64.05 | 23.53 | 3.78E-15 |
|  | *GhSus3A×*ENV | 24 | 22.89 | 0.95 | 0.35 | 1.00 |
|  | ***GhSus1D×GhSus3A*** | **2** | **278.45** | **139.23** | **51.14** | **8.82E-23** |
|  | Error | 7220 | 19655.78 | 2.72 |  |  |

Note: ENV means environment, each location and each year were combined and regarded as an individual environment.

**Table S4 the nucleotide sequences of *GhSus* family genes in TM-1**

Gene Chromosome cDNA sequence DNA sequence GenBank ID

***GhSus1At* A05**

ATGGCTGATCGTGTGATCACTCGCGTCCACAGTCTCCGTGAGCGTTTGGATGAGACCCTTCTTGCTCACAGGAACGAGATTTTGGCCTTGCTCTCAAGGATCGAGGGCAAAGGAAAAGGAATTCTGCAACACCATCAAATTATTCTAGAGTTTGAAGCTATCCCTGAAGAGAACAGAAAGAAGCTCGCTAATGGTGCATTTTTTGAAGTATTGAAGGCTAGTCAGGAAGCGATCGTGTTGCCTCCATGGGTTGCACTTGCTGTTCGTCCAAGGCCTGGTGTTTGGGAGTACATTAGAGTGAATGTTCACGCCCTTGTTGTTGAGGAACTCACTGTTGCTGAGTATCTCCACTTCAAGGAAGAGCTTGTTGATGGAAGTTCAAATGGAAACTTTGTTTTGGAATTGGATTTTGAGCCCTTCAACTCATCATTCCCCCGCCCAACTCTTTCAAAATCCATTGGTAATGGTGTGGAGTTCCTAAATCGTCACCTTTCGGCAAAATTGTTCCATGACAAGGAGAGCATGCACCCTTTGCTCGAATTCCTCAGAGTCCATTGTCACAAGGGCAAGAACATGATGTTGAATGACAGAATTCAGAACTTGAATGCTCTTCAACATGTTTTGAGGAAAGCAGAGGAGTATCTTGGTACCCTACCTCCTGAGACACCATGTGCCGAATTCGAACACCGGTTCCAGGAAATCGGTTTGGAAAGAGGTTGGGGTGACACCGCAGAACGCGTGCTCGAGATGATCCAACTCCTTTTGGATCTTCTTGAGGCACCTGATCCTTGCACCCTTGAGAAGTTCCTTGGGAGAATCCCTATGGTGTTCAATGTTGTGATTCTCACTCCCCACGGATACTTCGCTCAAGACAATGTTTTGGGGTATCCCGACACCGGTGGCCAGGTTGTTTACATCTTGGATCAAGTCCGAGCTTTGGAGAATGAGACGCTCCTCCGTATAAAGCAACAAGGACTCAACATCACCCCTCGAATCCTCATTATTACTAGACTTCTTCCTGATGCTGTCGGAACAACATGCGGTCAACGACTTGAGAAAGTATACGGAACAGAGCACTCGGATATTCTTCGAGTACCCTTCAGAACAGAAAAGGGAATTGTTCGTAAATGGATCTCAAGATTTGAAGTCTGGCCATACTTGGAAACCTACACAGAGGATGTTGCTCATGAAATCTCCAAAGAGTTGCAAGGCAAGCCAGATCTGATCATCGGAAACTACAGCGACGGCAATATCGTCGCCTCCTTGCTCGCACATAAATTAGGTGTCACACAGTGCACCATCGCCCATGCTTTGGAGAAGACAAAATATCCAGATTCAGATATCTATTGGAAGAAGCTTGAAGACAAATACCATTTCTCTTGCCAATTTACAGCTGATCTTTTTGCAATGAACCATACAGATTTCATCATCACCAGTACTTTCCAGGAAATTGCAGGAAGCAAGGACACTGTTGGTCAATACGAGAGCCACACTGCTTTCACTCTTCCTGGTCTCTACCGTGTTGTACATGGTATCGATGTGTTTGATCCCAAATTCAACATTGTTTCCCCTGGTGCTGATATGGAGATATACTTCCCTTACACCGAAGAGAAGCGGAGGTTGAAGCATTTCCATCCTGAGATCGAAGACCTTCTTTACAGCAAAGTTGAGAATGAAGAACACTTATGTGTGCTCAATGACCGCAACAAGCCAATTCTGTTCACAATGGCAAGGCTTGATCGTGTCAAGAACTTAACCGGACTCGTCGAGTGGTACGGCAAGAACGCAAAGTTGCGTGAGTTGGCTAACCTCGTAGTTGTAGGTGGTGATAGGCGAAAGGAATCTAAAGATTTGGAAGAGAAGGCTGAAATGAAGAAAATGTTTGAGCTGATCGACAAGTACAACTTGAACGGCCAATTCAGATGGATATCATCTCAAATGAACAGAATCCGAAATGGTGAACTTTACCGATACATTTGCGACACGAAAGGTGCCTTTGTACAGCCTGCATTGTATGAAGCCTTTGGATTGACAGTTGTGGAGGCAATGACTTGCGGTTTGCCAACATTCGCAACCTGTAACGGTGGACCAGCCGAGATTATTGTCCATGGGAAATCTGGTTTCAACATTGATCCTTACCATGGTGATCAAGCTGCTGACATACTCGTCGATTTCTTTGAAAAGTGTAAGAAAGATCCATCTCACTGGGATAAGATCTCCCAAGGAGGCTTGAAACGAATAGAGGAGAAGTATACATGGAAGATTTACTCGGAGAGACTATTGACCCTGACAGGAGTGTATGGATTCTGGAAGCATGTTTCCAACCTTGAACGCCGTGAGAGTCGTCGTTACCTTGAGATGTTTTATGCTCTTAAGTACCGTAAGCTGGCTGAATCAGTTCCATTGGCAGAGGAGTAA ATGGCTGATCGTGTGATCACTCGCGTCCACAGTCTCCGTGAGCGTTTGGATGAGACCCTTCTTGCTCACAGGAACGAGATTTTGGCCTTGCTCTCAAGGTAATTCGAATCTGATCCATCAATGACTGTTGCCCTGCATGGTGTTTTTCTTTTGAGATGTTTACATTTTGTTTATAAAATTGTTGGTTTTTTTTTGTAGGATCGAGGGCAAAGGAAAAGGAATTCTGCAACACCATCAAATTATTCTAGAGTTTGAAGCTATCCCTGAAGAGAACAGAAAGAAGCTCGCTAATGGTGCATTTTTTGAAGTATTGAAGGCTAGTCAGGTATAAGGAAAACCCATTTTATCCATTTCTTTTTTCATTATTTTGGGGGATTACAAGGTGTTGATTTTAGGGTCATTTTTGTGGTTATAGGAAGCGATCGTGTTGCCTCCATGGGTTGCACTTGCTGTTCGTCCAAGGCCTGGTGTTTGGGAGTACATTAGAGTGAATGTTCACGCCCTTGTTGTTGAGGAACTCACTGTTGCTGAGTATCTCCACTTCAAGGAAGAGCTTGTTGATGGAAGGTAACTATATGATCTGTTTGTATATGTATATTTGAGTTCATCTGATCTGTTGGTTGGTTTTTAACAATGTTTCTTTGCATTATTTGTAGTTCAAATGGAAACTTTGTTTTGGAATTGGATTTTGAGCCCTTCAACTCATCATTCCCCCGCCCAACTCTTTCAAAATCCATTGGTAATGGTGTGGAGTTCCTAAATCGTCACCTTTCGGCAAAATTGTTCCATGACAAGGAGAGCATGCACCCTTTGCTCGAATTCCTCAGAGTCCATTGTCACAAGGGCAAGGTATGCCTCATTTGCTTTTATTGCAAAAAAAATCTGTCAGAATTTGTCAGTGAATAGATCAGACAGATTTCTTGCATGCTGATACTAAGATTTACTAGATTCCTAGTACTGTCTGTTTTTTTTTCTGGATTTAAAATGCTGAGTTGTTCTTTTTGCAGAACATGATGTTGAATGACAGAATTCAGAACTTGAATGCTCTTCAACATGTTTTGAGGAAAGCAGAGGAGTATCTTGGTACCCTACCTCCTGAGACACCATGTGCCGAATTCGAACACCGGTTCCAGGAAATCGGTTTGGAAAGAGGTTGGGGTGACACCGCAGAACGCGTGCTCGAGATGATCCAACTCCTTTTGGATCTTCTTGAGGCACCTGATCCTTGCACCCTTGAGAAGTTCCTTGGGAGAATCCCTATGGTGTTCAATGTTGTGATTCTCACTCCCCACGGATACTTCGCTCAAGACAATGTTTTGGGGTATCCCGACACCGGTGGCCAGGTTGTTTACATCTTGGATCAAGTCCGAGCTTTGGAGAATGAGACGCTCCTCCGTATAAAGCAACAAGGACTCAACATCACCCCTCGAATCCTCATTGTGAGACTGCTAGTAGTTTTAATCCTTCCTTTCGGCTTTTTACTATTTTGTATGCTCATATCTCTGTTTCCTCTTTCAGATTACTAGACTTCTTCCTGATGCTGTCGGAACAACATGCGGTCAACGACTTGAGAAAGTATACGGAACAGAGCACTCGGATATTCTTCGAGTACCCTTCAGAACAGAAAAGGGAATTGTTCGTAAATGGATCTCAAGATTTGAAGTCTGGCCATACTTGGAAACCTACACAGAGGTGAAACTCATGAGCCATTGATTCATTTTGTATGATGGTAGATCACATTAGTTTTATTTACGAGTAAATATGTTTGTTTCATAGGATGTTGCTCATGAAATCTCCAAAGAGTTGCAAGGCAAGCCAGATCTGATCATCGGAAACTACAGCGACGGCAATATCGTCGCCTCCTTGCTCGCACATAAATTAGGTGTCACACAGGTTTGTTAGAAACTAATTTGCCGGATCTTGTATCTTCTCGTATATTCGGTCGGGTCGGATTACCTTACAATGACTTGGTTTTTCACCCTTGTGTAGTGCACCATCGCCCATGCTTTGGAGAAGACAAAATATCCAGATTCAGATATCTATTGGAAGAAGCTTGAAGACAAATACCATTTCTCTTGCCAATTTACAGCTGATCTTTTTGCAATGAACCATACAGATTTCATCATCACCAGTACTTTCCAGGAAATTGCAGGAAGGTAATAAATAGATGATTTAATTCTCAGTACTTGAGATATTTTATTAATGCTCGGCGGATAATGAAGATCATTAACATTAATAAAATTCTCAAATGGTTTTCAGCAAGGACACTGTTGGTCAATACGAGAGCCACACTGCTTTCACTCTTCCTGGTCTCTACCGTGTTGTACATGGTATCGATGTGTTTGATCCCAAATTCAACATTGTTTCCCCTGGTGCTGATATGGAGATATACTTCCCTTACACCGAAGAGAAGCGGAGGTTGAAGCATTTCCATCCTGAGATCGAAGACCTTCTTTACAGCAAAGTTGAGAATGAAGAACACTTGTAAGTGCAAGCTTTTTTATATGTTTTAGCAGAATCCGATTGTGGGAACATATCCCGTCTGAATTAATGATTTGTCCCTGATTTTCCTACAGATGTGTGCTCAATGACCGCAACAAGCCAATTCTGTTCACAATGGCAAGGCTTGATCGTGTCAAGAACTTAACCGGACTCGTCGAGTGGTACGGCAAGAACGCAAAGTTGCGTGAGTTGGCTAACCTCGTAGTTGTAGGTGGTGATAGGCGAAAGGAATCTAAAGATTTGGAAGAGAAGGCTGAAATGAAGAAAATGTTTGAGCTGATCGACAAGTACAACTTGAACGGCCAATTCAGATGGATATCATCTCAAATGAACAGAATCCGAAATGGTGAACTTTACCGATACATTTGCGACACGAAAGGTGCCTTTGTACAGCCTGCATTGTATGAAGCCTTTGGATTGACAGTTGTGGAGGCAATGACTTGCGGTTTGCCAACATTCGCAACCTGTAACGGTGGACCAGCCGAGATTATTGTCCATGGGAAATCTGGTTTCAACATTGATCCTTACCATGGTGATCAAGCTGCTGACATACTCGTCGATTTCTTTGAAAAGTGTAAGAAAGATCCATCTCACTGGGATAAGATCTCCCAAGGAGGCTTGAAACGAATAGAGGAGAAGTAAGCTCTCACACACACACAATAAAGAAAAAACCATTTTCCACTTGGGTTTATATTGTTTAGTATTTGTTGTAACGATTATAAATTGTCTGAATCAGGTATACATGGAAGATTTACTCGGAGAGACTATTGACCCTGACAGGAGTGTATGGATTCTGGAAGCATGTTTCCAACCTTGAACGCCGTGAGAGTCGTCGTTACCTTGAGATGTTTTATGCTCTTAAGTACCGTAAGCTGGTAATTCACATGGTCACATCCCCATTCTGTATAAATGTCCTTTATTTGGCTTTAATTACATTGTTGGTGATTTGTAAAATGCAGGCTGAATCAGTTCCATTGGCAGAGGAGTAAATTGAAGCTGTTAAATAACATTGGGCCGGTTTTTCTTGGAGAATAATATTCTGTTTTGTAATTTCAATTGGAGAAGCTCCTTTGTATTTCATCTTGTCTTTTCCTTTTCCTTTTTTCGCCGGCATTGTTTGAACATGGGGTTGTGCGCCCGTCAATTCC HQ143089*

***GhSus1Dt* D05** ATGGCTAATCCTGTGATCACTCGCGTCCACAGTCTCCGTGAGCGTTTAGATGAGACCCTTCTTGCCCACAGGAACGAGATTTTGGCCTTGCTCTCAAGGATCGAGGGCAAAGGAAAAGGAATTCTGCAACACCATCAAATTATTCTAGAGTTTGAAGCTATCCCTGAAGAGAACAGAAAGAAGCTCGCTGATGGTGCATTTTTTGAAGTATTGAAGGCTAGTCAGGAAGCGATCGTGTTGCCTCCATGGGTTGCACTTGCTGTTCGTCCAAGGCCTGGTGTTTGGGAGTACATTAGAGTGAATGTTCACGCCCTTGTTGTTGAGGAACTTACTGTTGCTGAGTATCTCCACTTCAAGGAAGAGCTTGTTGATGGAAGTTCAAATGGAAACTTTGTTTTGGAATTGGATTTTGAGCCCTTCAACTCATCATTCCCCCGCCCAACTCTTTCAAAATCCGTTGGTAATGGTGTGGAGTTCCTAAATCGTCACCTTTCGGCAAAATTGTTCCATGACAAGGAGAGCATGCACCCTTTGCTCGAATTCCTCAGAGTCCATTGCCACAAGGGCAAGAACATGATGTTGAATGACAGAATTCAGAACTTGAATGCTCTTCAACATGTTTTGAGGAAAGCAGAGGAGTATCTTGGTACCCTACCTCCTGAGACACCATGTGCCGGATTCGAACACCGGTTCCAGGAAATCGGTTTGGAAAGAGGTTGGGGTGACACCGCACAACGCGTGCTCGAGATGATCCAACTCCTTTTGGATCTTCTTGAGGCACCTGATCCTTGCACCCTTGAGAAGTTCCTTGGGAGAATCCCCATGGTGTTCAATGTTGTGATTCTCACTCCCCACGGATACTTCGCTCAAGACAATGTTTTGGGGTATCCCGACACCGGTGGCCAGGTTGTTTACATCTTGGATCAAGTCCGAGCTTTGGAGAATGAGATGCTCCTCCGTATAAAGCAACAAGGACTCAACATCACCCCTCGAATCCTCATTATTACTAGACTTCTTCCTGATGCTGTCGGAACAACATGCGGTCAACGACTTGAGAAAGTATACGGAACAGAGTACTCGGATATTCTTCGAGTACCCTTCAGAACAGAAAAGGGAATTGTTCGTAAATGGATCTCAAGATTTGAAGTCTGGCCATACTTGGAAACCTACACAGAGGATGTTGCTCATGAAATCTCCAAAGAGTTGCAAGGCAAGCCAGATCTGATCATCGGAAACTACAGTGATGGCAATATCGTCGCCTCCTTGCTCGCACATAAATTGGGTGTCACACAGTGCACCATCGCCCATGCTTTGGAGAAGACAAAATATCCTGATTCAGATATCTACTGGAAGAAGCTTGAAGACAAATACCATTTCTCTTGCCAATTTACAGCTGATCTTTTTGCAATGAACCATACAGATTTCATCATCACCAGTACTTTCCAGGAAATTGCAGGAAGCAAGGACACTGTTGGTCAATACGAGAGCCACACTGCTTTCACTCTTCCTGGTCTCTACCGTGTTGTACATGGTATCGATGTGTTTGATCCCAAATTCAACATTGTTTCCCCTGGTGCTGATATGGAGATATACTTCCCTTACACCGAAGAGAAGCGGAGGTTGAAGCATTTCCATACTGAGATCGAAGACCTTCTTTACAGCAAAGTTGAGAATGAAGAACACTTATGTGTGCTCAATGACCGCAACAAGCCAATTCTGTTCACAATGGCAAGGCTTGATCGTGTCAAGAACTTAACCGGACTCGTCGAGTGGTACGGCAAGAACGCAAAGTTGCGTGAGTTGGCTAACCTCGTAGTTGTAGGTGGTGATAGGCGAAAGGAATCTAAAGATTTGGAAGAGAAGGCCGAAATGAAGAAAATGTTTGAGCTGATCGAGAAGTACAACTTGAACGGCCAATTCAGATGGATATCATCTCAAATGAACAGAATCCGAAATGGTGAACTTTACCGATACATTTGCGACACGAAAGGTGCCTTTGTACAGCCTGCATTGTATGAAGCCTTTGGATTGACAGTTGTGGAGGCAATGACTTGCGGTTTGCCAACATTCGCAACCTGCAACGGTGGACCAGCCGAGATTATTGTCCATGGGAAATCCGGTTTCAACATTGATCCTTACCATGGTGATCAAGCTGCTGACATACTGGTCGATTTCTTTGAAAAGTGTAAGAAAGATCCATCTCACTGGGATAAGATCTCCCAAGGAGGCTTGAAACGTATCGAGGAGAAGTATACATGGAAGATTTACTCGGAGAGACTATTGACCCTGACCGGAGTGTATGGATTCTGGAAGCATGTTTCCAACCTTGAACGCCGTGAGAGTCGTCGTTACCTTGAGATGTTTTATGCTCTTAAGTACCGCAAGCTGGCTGAATCAGTTCCATTGGCAGAGGAGTAA ATGGCTAATCCTGTGATCACTCGCGTCCACAGTCTCCGTGAGCGTTTAGATGAGACCCTTCTTGCCCACAGGAACGAGATTTTGGCCTTGCTCTCAAGGTAATTTGAATCTGATCCATCAATGACTGTTTCCCTGCATGGTGTTTTTCTTTTGAGATGTTTACATTTTGTTTATAAAATTGTTGGTTTTTTATTGTAGGATCGAGGGCAAAGGAAAAGGAATTCTGCAACACCATCAAATTATTCTAGAGTTTGAAGCTATCCCTGAAGAGAACAGAAAGAAGCTCGCTGATGGTGCATTTTTTGAAGTATTGAAGGCTAGTCAGGTATAAGGAAAACCCATTTTCTCCATTTCTTTTTTCATTATTTTGGGGGATTACAAGGTGTTGATTTTAGGATGATTTTTGGGGTTATAGGAAGCGATCGTGTTGCCTCCATGGGTTGCACTTGCTGTTCGTCCAAGGCCTGGTGTTTGGGAGTACATTAGAGTGAATGTTCACGCCCTTGTTGTTGAGGAACTTACTGTTGCTGAGTATCTCCACTTCAAGGAAGAGCTTGTTGATGGAAGGTAACTATATGATCTGTTTGTATATGTATATTTGAGTTCATCTGATCTGTTTGTTGGTTTTTAACAATGTTTCTTTGCATTGTTTGTAGTTCAAATGGAAACTTTGTTTTGGAATTGGATTTTGAGCCCTTCAACTCATCATTCCCCCGCCCAACTCTTTCAAAATCCGTTGGTAATGGTGTGGAGTTCCTAAATCGTCACCTTTCGGCAAAATTGTTCCATGACAAGGAGAGCATGCACCCTTTGCTCGAATTCCTCAGAGTCCATTGCCACAAGGGCAAGGTATGCCTCATTTGCTTATTTTATTGCAAAAAAAATCTGTCAGAATTTGTCAGTGGATAGACCAGACAGATTTCTTGCATGCTGATACTAAGATTTACTAGATTCATGGTACTGTCTTTTCTGGATTTAAAATGCTGAGTTGTTCTTTTTGCAGAACATGATGTTGAATGACAGAATTCAGAACTTGAATGCTCTTCAACATGTTTTGAGGAAAGCAGAGGAGTATCTTGGTACCCTACCTCCTGAGACACCATGTGCCGGATTCGAACACCGGTTCCAGGAAATCGGTTTGGAAAGAGGTTGGGGTGACACCGCACAACGCGTGCTCGAGATGATCCAACTCCTTTTGGATCTTCTTGAGGCACCTGATCCTTGCACCCTTGAGAAGTTCCTTGGGAGAATCCCCATGGTGTTCAATGTTGTGATTCTCACTCCCCACGGATACTTCGCTCAAGACAATGTTTTGGGGTATCCCGACACCGGTGGCCAGGTTGTTTACATCTTGGATCAAGTCCGAGCTTTGGAGAATGAGATGCTCCTCCGTATAAAGCAACAAGGACTCAACATCACCCCTCGAATCCTCATTGTGAGACTGCTAGTAGTTTTAATCCTTCCTTTCGGCTTTTTACTATTTTGTATGCTTATATCTCTGTTTCCTCTTTCAGATTACTAGACTTCTTCCTGATGCTGTCGGAACAACATGCGGTCAACGACTTGAGAAAGTATACGGAACAGAGTACTCGGATATTCTTCGAGTACCCTTCAGAACAGAAAAGGGAATTGTTCGTAAATGGATCTCAAGATTTGAAGTCTGGCCATACTTGGAAACCTACACAGAGGTGAAACTTATGAGCCATTGATTCATTTTGTATGATGGTAGATCACATTAGTTTTATTTACGAGTAAATATGTTTGTTTCATAGGATGTTGCTCATGAAATCTCCAAAGAGTTGCAAGGCAAGCCAGATCTGATCATCGGAAACTACAGTGATGGCAATATCGTCGCCTCCTTGCTCGCACATAAATTGGGTGTCACACAGGTTTGTTAAAAACTAATTTGCCGGATCTTGTATCTTCTCGTTTATTCGGTCGGGTCGGATTACAGTACAATGACTTGGTTTTTCACCCTTGTGTAGTGCACCATCGCCCATGCTTTGGAGAAGACAAAATATCCTGATTCAGATATCTACTGGAAGAAGCTTGAAGACAAATACCATTTCTCTTGCCAATTTACAGCTGATCTTTTTGCAATGAACCATACAGATTTCATCATCACCAGTACTTTCCAGGAAATTGCAGGAAGGTAATAAATAGATGATTTAATTCTCTCAGTACTTGAGATGTTTTATTAATTCTCGGCGGATAATGAAGATCATTAACATTAATAAAATTCTCAAATGGTTTTCAGCAAGGACACTGTTGGTCAATACGAGAGCCACACTGCTTTCACTCTTCCTGGTCTCTACCGTGTTGTACATGGTATCGATGTGTTTGATCCCAAATTCAACATTGTTTCCCCTGGTGCTGATATGGAGATATACTTCCCTTACACCGAAGAGAAGCGGAGGTTGAAGCATTTCCATACTGAGATCGAAGACCTTCTTTACAGCAAAGTTGAGAATGAAGAACACTTGTAAGTGCAAGCTTTTTTATATGTTTTAGCAAAATCCGATTGTGGGAACATATCCCGTCTGAACTAATGATTTGTCCCTGATTTTCCTACAGATGTGTGCTCAATGACCGCAACAAGCCAATTCTGTTCACAATGGCAAGGCTTGATCGTGTCAAGAACTTAACCGGACTCGTCGAGTGGTACGGCAAGAACGCAAAGTTGCGTGAGTTGGCTAACCTCGTAGTTGTAGGTGGTGATAGGCGAAAGGAATCTAAAGATTTGGAAGAGAAGGCCGAAATGAAGAAAATGTTTGAGCTGATCGAGAAGTACAACTTGAACGGCCAATTCAGATGGATATCATCTCAAATGAACAGAATCCGAAATGGTGAACTTTACCGATACATTTGCGACACGAAAGGTGCCTTTGTACAGCCTGCATTGTATGAAGCCTTTGGATTGACAGTTGTGGAGGCAATGACTTGCGGTTTGCCAACATTCGCAACCTGCAACGGTGGACCAGCCGAGATTATTGTCCATGGGAAATCCGGTTTCAACATTGATCCTTACCATGGTGATCAAGCTGCTGACATACTGGTCGATTTCTTTGAAAAGTGTAAGAAAGATCCATCTCACTGGGATAAGATCTCCCAAGGAGGCTTGAAACGTATCGAGGAGAAGTAAGCTCTCACACACACACAAGAAAGAAAAAACCATTTTCCTCTTGGGTTTATATTGTTTAGTATTTGTTGTAACGAGTATAAATTGTCTGAATCAGGTATACATGGAAGATTTACTCGGAGAGACTATTGACCCTGACCGGAGTGTATGGATTCTGGAAGCATGTTTCCAACCTTGAACGCCGTGAGAGTCGTCGTTACCTTGAGATGTTTTATGCTCTTAAGTACCGCAAGCTGGTAATTCACATAGTCACATCCCCATTCTGTATAAATGTCCTTCATTTGGCTTTAATTACATTGTTGGTTATTTGTAAAATGCAGGCTGAATCAGTTCCATTGGCAGAGGAGTAAATTTAAGCTGTTAAATAACATTGGGCCGGTTTTTCTTGGAGAATAATATTCTGTTTTGTAATTTCAATTGGAGAAGCTCTTTTGTATTTCATCTTGTCTTTTCCTTTTCCTTTTTTCGCCGGCATTGTTTGAACATGGGGTTGTGCGCCCGTCAATTCC HQ143090

***GhSus2At* A13** ATGGCTGAGCGTGCTCTCACTCGCGTCCACAGTCTCCGTGAGCGTTTGGATTCCACGCTTACCGCCCACAGGAACGAGATTTTGGCTTTGCTCTCAAGGATTGAAGGTAAAGGAAAAGGAATCCTTCTTCATCATCAAATCATTCTTGAGTTTGAAGCTATCCCTGAAGAGAACCGAAAGAAATTAGCTGATGGTGCATTTTTTGAAATACTGAAAGCCAGTCAGGAAGCCATCGTGTTGCCGCCGTGGGTGGCGCTGGCCGTCCGACCAAGACCTGGTGTTTGGGAGTACATTAGAGTTAATGTTCATGCTCTTGTTGTTGAAGAACTCACTGTTGCTGAGTATCTTCGTTTCAAGGAAGAGCTTGTTGATGGAAGTTCAAATGCAAACTTTGTTTTGGAACTGGATTTCGAGCCCTTCAACGCGTCGTTCCCTCGCCCGACTCTTTCGAAATCGATCGGTAACGGCGTCGAGTTCCTTAATCGCCACCTTTCGGCCAAACTGTTCCATGACAAGGAGAGCATGCACCCTTTGCTTGAATTCCTGAAAGTCCATTGTCATAAGGGCAAGAACATGATGTTGAATGATAGGATTCAAAACTTGAATTCTCTCCAACATGTTTTGAGGAAGGCAGAGGAGTATCTTGTTGCACTGCCGGCCGAGACTCCTTATGCCGAATTCGAACACAAGTTCCAGGAGATTGGTTTGGAGAGAGGGTGGGGTGATACGGCTGAGCGTGTGCTCGAGATGATCCAACTCCTTTTGGATCTTCTCGAGGCACCCGATCCTTGTACCCTTGAGAAGTTCCTCGGGAGAATTCCCATGGTGTTCAATGTTGTGATTCTTACGCCTCACGGTTACTTTGCTCAGGACAATGTTTTGGGGTATCCCGACACCGGTGGCCAGGTTGTTTACATCTTGGATCAAGTCCGTGCCTTGGAGAACGAAATGCTCAACCGTATCAAGCAACAAGGACTCAACATTACCCCTCGTATTCTCATTATTACTCGACTTCTCCCTGATGCCGTGGGAACAACTTGCGGTCAACGGCTCGAGAAAGTATATGGGACTGAGTACTCGGATATTCTCCGAATACCCTTCAGAACAGAGAAAGGAATCGTACGTAGATGGATCTCAAGATTTGAAGTCTGGCCTTACTTGGAAACTTACACCGAGGATGTTGCTCATGAGATTTCAAAAGAGTTGCAAGGCAAGCCCGATTTAATCATCGGAAACTACAGTGATGGTAATATCGTTGCCTCCTTGCTGGCACATAAGTTGGGAGTTACACAGTGTACGATTGCTCACGCTTTGGAGAAGACAAAGTATCCAGATTCCGACATCTACTGGAAGAAGCTCGAGGATAAATATCATTTCTCCTGCCAATTTGCAGCTGATCTTTTCGCTATGAACCATACGGATTTCATCATCACCAGTACCTTCCAAGAAATTGCTGGAAGCAAGGACACTGTTGGTCAATACGAGAGTCACACTGCTTTCACTCTTCCCGGTCTCTACCGTGTTGTTCACGGAATTGATGTGTTTGATCCTAAATTCAACATTGTGTCCCCTGGTGCCGACATGAGCATATACTACCCTTACACCGAGGAGAAGAAGAGGTTGAAGCATTTCCATTCCGAGATTGAAGAACTTCTTTACAGCAAAGTTGAAAATGAAGAACACTGGTGTGTGCTAAACGACCGCAACAAGCCAATTCTATTTACAATGGCAAGGCTGGATCGTGTTAAGAATTTAACTGGACTTGTTGAATGGTACGGGAAGAATGCTAAGTTGCGTGAATTGGTTAACCTTGTAGTTGTCGGTGGAGATCGGAGAAAAGAGTCCAAGGATTTGGAAGAGAAGGCTGAGATGAAGAAGATGTTCGAGCTCATTGAGAAGTACAAGCTGAACGGTCAGTTCAGATGGATATCATCCCAAATGAACAGAGTTAGGAACGGTGAACTTTACCGTTACATTTGTGACACAAAGGGTGCCTTTGTGCAACCCGCATTATATGAAGCCTTTGGGTTGACTGTCGTTGAGGCCATGACTTGTGGTTTGCCGACATTCGCAACTTGCAATGGTGGACCTGCCGAGATTATCGTTCATGGTAAATCCGGCTTCAACATTGATCCTTACCACGGCGACCAAGCTGCTGAGATCCTTGTCGACTTCTTTGAGAAATGCAAGACAGATCCATCTTACTGGACCAAGATCTCCGAGGGAGGTTTGAAACGTATCGAAGAGAAGTACACATGGAAAATTTACTCTGAGAGACTATTAACCTTGACCGGTGTCTACGGGTTCTGGAAGCATGTGTCCAACCTCGACCGCCTCGAGAGCCGTCGTTACCTTGAGATGTTTTATGCTCTTAAGTACCGTAAGCTGGCCGAATCGGTACCTTTGGCAGTCGAAGAGTAA ATGGCTGAGCGTGCTCTCACTCGCGTCCACAGTCTCCGTGAGCGTTTGGATTCCACGCTTACCGCCCACAGGAACGAGATTTTGGCTTTGCTCTCAAGGTAATTCAGACAAGATCCTTAAAAAAAGATTTTGTGTTTTTTCAATAAAATTTGCATGCAAGTTTTGAAATTGTTGGCTTTTTTTTTTGGCTATAGGATTGAAGGTAAAGGAAAAGGAATCCTTCTTCATCATCAAATCATTCTTGAGTTTGAAGCTATCCCTGAAGAGAACCGAAAGAAATTAGCTGATGGTGCATTTTTTGAAATACTGAAAGCCAGTCAGGTTTAAACAAACCCCCCTTTATTTTTATATTTTTTAAAGAAAATATACTTTGGGTTGTTTTTTGTTTGCAAAATTTTGATTTTTTTGGTGTCTATAGGAAGCCATCGTGTTGCCGCCGTGGGTGGCGCTGGCCGTCCGACCAAGACCTGGTGTTTGGGAGTACATTAGAGTTAATGTTCATGCTCTTGTTGTTGAAGAACTCACTGTTGCTGAGTATCTTCGTTTCAAGGAAGAGCTTGTTGATGGAAGGTATACTAATAGTAATAACATGAATTGATTGAATTTTGATCGAGTTTTGTTTAATCTGTTTATTAAAGGCTTTGTTTTTTGCTCACAGTTCAAATGCAAACTTTGTTTTGGAACTGGATTTCGAGCCCTTCAACGCGTCGTTCCCTCGCCCGACTCTTTCGAAATCGATCGGTAACGGCGTCGAGTTCCTTAATCGCCACCTTTCGGCCAAACTGTTCCATGACAAGGAGAGCATGCACCCTTTGCTTGAATTCCTGAAAGTCCATTGTCATAAGGGCAAGGTAACATATGCTGCTGCCTCGTATTTGTCGATTTATTGTGCTTTCGTAGATCGGACCTGGACTCTTGCATGTTGATGATTCTGAGGGGTTCATTATGTTTTTGCAGAACATGATGTTGAATGATAGGATTCAAAACTTGAATTCTCTCCAACATGTTTTGAGGAAGGCAGAGGAGTATCTTGTTGCACTGCCGGCCGAGACTCCTTATGCCGAATTCGAACACAAGTTCCAGGAGATTGGTTTGGAGAGAGGGTGGGGTGATACGGCTGAGCGTGTGCTCGAGATGATCCAACTCCTTTTGGATCTTCTCGAGGCACCCGATCCTTGTACCCTTGAGAAGTTCCTCGGGAGAATTCCCATGGTGTTCAATGTTGTGATTCTTACGCCTCACGGTTACTTTGCTCAGGACAATGTTTTGGGGTATCCCGACACCGGTGGCCAGGTTAGCAATTACGAGATTTCAATGTTGTAATTCGTGTATAAAACAAATTTGTTAACTCTAAATCTTTCGACTCTCCCATGCAGGTTGTTTACATCTTGGATCAAGTCCGTGCCTTGGAGAACGAAATGCTCAACCGTATCAAGCAACAAGGACTCAACATTACCCCTCGTATTCTCATTGTGAGTGTTCATGATCTTTCGATTCGGTTTTATTTGATTTCGGCTGTTATTCATGCTTACAGTACATCTCTTTTGAACTTTTTCTAGATTACTCGACTTCTCCCTGATGCCGTGGGAACAACTTGCGGTCAACGGCTCGAGAAAGTATATGGGACTGAGTACTCGGATATTCTCCGAATACCCTTCAGAACAGAGAAAGGAATCGTACGTAGATGGATCTCAAGATTTGAAGTCTGGCCTTACTTGGAAACTTACACCGAGGTGAGCCATATGAACCATTGAATTTCTTGCATTAGTTTACCCCCATTTCTTTGATTTCATTTACCGAGTATAATCTCTCTTTGTTTTAAGGATGTTGCTCATGAGATTTCAAAAGAGTTGCAAGGCAAGCCCGATTTAATCATCGGAAACTACAGTGATGGTAATATCGTTGCCTCCTTGCTGGCACATAAGTTGGGAGTTACACAGGTTTGTTAGAAACTAATTTACTGGGATATTTTTACGTGTTTATCTTCAATATTGTTTTGGTGTATCGATTCTATTTTTCATTCTATTGTAGTGTACGATTGCTCACGCTTTGGAGAAGACAAAGTATCCAGATTCCGACATCTACTGGAAGAAGCTCGAGGATAAATATCATTTCTCCTGCCAATTTGCAGCTGATCTTTTCGCTATGAACCATACGGATTTCATCATCACCAGTACCTTCCAAGAAATTGCTGGAAGGTAGATGTTCGGATTTGATATTTTTGTGTGAAATGCTTTTCTCTATAAACCGTTAACATCGATATTCTTATGATTTCAGCAAGGACACTGTTGGTCAATACGAGAGTCACACTGCTTTCACTCTTCCCGGTCTCTACCGTGTTGTTCACGGAATTGATGTGTTTGATCCTAAATTCAACATTGTGTCCCCTGGTGCCGACATGAGCATATACTACCCTTACACCGAGGAGAAGAAGAGGTTGAAGCATTTCCATTCCGAGATTGAAGAACTTCTTTACAGCAAAGTTGAAAATGAAGAACACTGGTGTGTGCTAAACGACCGCAACAAGCCAATTCTATTTACAATGGCAAGGCTGGATCGTGTTAAGAATTTAACTGGACTTGTTGAATGGTACGGGAAGAATGCTAAGTTGCGTGAATTGGTTAACCTTGTAGTTGTCGGTGGAGATCGGAGAAAAGAGTCCAAGGATTTGGAAGAGAAGGCTGAGATGAAGAAGATGTTCGAGCTCATTGAGAAGTACAAGCTGAACGGTCAGTTCAGATGGATATCATCCCAAATGAACAGAGTTAGGAACGGTGAACTTTACCGTTACATTTGTGACACAAAGGGTGCCTTTGTGCAACCCGCATTATATGAAGCCTTTGGGTTGACTGTCGTTGAGGCCATGACTTGTGGTTTGCCGACATTCGCAACTTGCAATGGTGGACCTGCCGAGATTATCGTTCATGGTAAATCCGGCTTCAACATTGATCCTTACCACGGCGACCAAGCTGCTGAGATCCTTGTCGACTTCTTTGAGAAATGCAAGACAGATCCATCTTACTGGACCAAGATCTCCGAGGGAGGTTTGAAACGTATCGAAGAGAAGTAAGCACTCATTTTCTTCATTTCCTCGATTCTTTTTGCCCAAGTCAACTCCTGTCATTGTTCTTACATACCTGCATGGTTCTGATCAGGTACACATGGAAAATTTACTCTGAGAGACTATTAACCTTGACCGGTGTCTACGGGTTCTGGAAGCATGTGTCCAACCTCGACCGCCTCGAGAGCCGTCGTTACCTTGAGATGTTTTATGCTCTTAAGTACCGTAAGCTGGTAATTCTCATGCCCCATTTCTTTTTATCCAGTTTAAATACGAATGACTTGCATTGTTAATGTTTTTATAACTGTTTTTCGCAGGCCGAATCGGTACCTTTGGCAGTCGAAGAGTAA null§

***GhSus3At* A06** ATGGCAGAGCGGGTGATCACCCGAGTACACAGCCTCCGAGAGCGTCTGGATGACACCCTTATTGCCCATAGAAACGAGGTTTTGGCCTTGCTCACAAGGATCGAGGGTAAGGGAAAAGGGATTCTGCAACACCATCAAATTATCCTAGAGTTTGAAGCCATCCCTGAAGAAACCAGAAAGAAGCTCGCTGATGGAGCATTTTCTGAAATATTGAGATCCAGTCAGGAAGCGATCGTGTTGCCACCATGGGTTGCACTTGCAGTTCGTCCAAGGCCTGGTGTTTGGGAGTATATTAAAGTGAATGTCCACGCCCTTGTTGTTGAGGAACTCACTGTTGCAGAGTATCTTCACTTCAAGGAGGAGCTTGTTGATGGAAGTGCAAATGGCAATTTCGTTTTGGAATTGGATTTTGAGCCCTTCAATGCATCTTTCCCTCGCTCAACTCTTTCCAAGTCTATCGGTAATGGTGTTGAGTTCCTCAATCGCCACCTTTCGGCTAAATTGTTCCATGACAAGGAGAGTATGCACCCTTTGCTTGAATTCCTCAAAGTCCATTGCCACAAAGGAAAGAACATGATGTTGAATGACAGAATTCAAAACCTGAATTCCCTTCAATATGTATTGAGGAAGGCAGAAGAATATCTTGGTACACTGCCAGCTGAGACACCGTACACTGAACTGGAACACAAGTTCCAGGAAATTGGTTTGGAGAGAGGTTGGGGTGATACCGCGGGGCGTGTGCTGGAGATGATCCAACTCCTTTTGGATCTTCTCGAGGCCCCTGATCCTTGCACCCTTGAGAAGTTCCTCGGGAGAGTCCCCATGGTGTTCAATGTTGTCATCCTTACTCCTCACGGATACTTTGCTCAAGACAACGTTTTGGGGTACCCCGACACCGGTGGCCAGGTTGTATACATCTTGGATCAAGTCCGTGCCTTGGAGAACGAGATGCTCCTCCGTATCAAGCAGCAAGGACTCAACATTACCCCTCGAATCTTAATTATTACCAGACTTCTCCCTGACGCTGTTGGAACAACTTGTGGTCAACGAGTTGAAAAGGTATACGGAACGGAATACTCCGACATCCTCCGAGTACCCTTTAGAACAGAGAAGGGAATTGTACGTAGATGGATCTCGAGATTCGTAGTCTGGCCCTACTTGGAAACTTACACTGAGGATGTTGCTCACGAAATTTCCAAAGAGTTGCAAGGCAAGCCCGATCTCATCATCGGAAACTATAGTGACGGCAACATTGTTGCCTCGTTGCTGGCTCACAAATTGGGAGTCACACAGTGTACCATTGCCCATGCTTTGGAGAAAACAAAGTACCCAGATTCCGATATTTACTGGAAGAAGCTAGAGGATAAATACCATTTCTCCTGCCAATTTACAGCTGATCTTATTGCAATGAACCATACAGATTTCATCATCACTAGTACTTTCCAAGAAATTGCAGGAAGCAAGGACACTGTTGGTCAATATGAGAGTCACACTGCTTTCACTCTTCCTGGTTTATACCGAGTCGTACACGGTATCGATGTATTTGATCCCAAATTCAACATCGTGTCTCCCGGTGCTGACATGAGCATATACTTCCCTTACACCGAGAAGAAGAGGAGGTTGAAGCATTTCCACCCCGAGATTGAAGACCTCCTTTATAGCAAAGTCGAGAACGAAGAACACTTATGTGTGCTAAATGACCGCAACAAGCCGATCCTATTCACGATGGCAAGGCTAGACCGTGTTAAGAACTTAACCGGACTCGTCGAGTGGTACGGCAAGAATGCAAAGCTGCGCGAGTTGGTTAACCTCGTAGTCGTAGGTGGAGACAGGAGAAAGGAATCCAAAGATTTAGAAGAGAAGGCCGAAATGAAGAAGATGTTTGAGCTGATAAAAACATACAAATTGAACGGTCAATTCAGATGGATATCATCGCAAATGAACCGAGTTAGGAATGGTGAGTTGTACCGCTACATTTGCGACACAAAAGGTGCCTTCGTACAACCAGCATTGTACGAAGCCTTTGGATTAACGGTTGTTGAAGCCATGACTTGCGGATTGCCAACATTTGCTACCTGCAAAGGTGGACCAGCTGAAATCATTGTCCACGGTAAATCTGGGTTCAACATTGATCCTTACCATGGTGATCAAGCTGCAGAAATCCTTGTTGATTTCTTCGACAAATGTAAAAAGGAACCATCTCACTGGAATGACATCTCTGAGGGTGGCTTGAAACGTATCCAGGAGAAATACACATGGCAGATATATTCGGAGAGGCTATTAACATTGACGGGAGTGTATGGATTCTGGAAGCACGTTTCCAACCTTGACCGTCGCGAGAGCCGCCGTTACCTGGAGATGTTTTACGCTCTTAAGTACCGCAAGCTGGCTGAATCGGTTCCTCTGGCAGAGGAGGAGTAA ATGGCAGAGCGGGTGATCACCCGAGTACACAGCCTCCGAGAGCGTCTGGATGACACCCTTATTGCCCATAGAAACGAGGTTTTGGCCTTGCTCACAAGGTAACTTAAATATAACCCCTTCAACGATTGCTTTTCCTTTTGCAAATGCTTAGTTTTGTTGTTTTCTATTGTAGGATCGAGGGTAAGGGAAAAGGGATTCTGCAACACCATCAAATTATCCTAGAGTTTGAAGCCATCCCTGAAGAAACCAGAAAGAAGCTCGCTGATGGAGCATTTTCTGAAATATTGAGATCCAGTCAGGTATAAAACAAACCCTTTTTGTGTTTTCTCTTATGGGTTTTATTGTGACACTAGTGTTGATTTTAGTGACATTTGTGTTGTAGGAAGCGATCGTGTTGCCACCATGGGTTGCACTTGCAGTTCGTCCAAGGCCTGGTGTTTGGGAGTATATTAAAGTGAATGTCCACGCCCTTGTTGTTGAGGAACTCACTGTTGCAGAGTATCTTCACTTCAAGGAGGAGCTTGTTGATGGAAGGTATCTTGAAAATTGTTCATTTTCTGTTTACTTCACTCGTAAATGCTCATTTTAACACTGTTTCTTTGATTTTTCTTGCAGTGCAAATGGCAATTTCGTTTTGGAATTGGATTTTGAGCCCTTCAATGCATCTTTCCCTCGCTCAACTCTTTCCAAGTCTATCGGTAATGGTGTTGAGTTCCTCAATCGCCACCTTTCGGCTAAATTGTTCCATGACAAGGAGAGTATGCACCCTTTGCTTGAATTCCTCAAAGTCCATTGCCACAAAGGAAAGGTATGCAACATAAAGCACCAGTTGCTTGTGTTTTATAGTTGAAATCTGCATATACTAAAACATAGATTTCTTAGCATAAGATTTAAATGATGGATTTAGTGTCCTGAATGTCTTAGTTGGTGTTTTTGCAGAACATGATGTTGAATGACAGAATTCAAAACCTGAATTCCCTTCAATATGTATTGAGGAAGGCAGAAGAATATCTTGGTACACTGCCAGCTGAGACACCGTACACTGAACTGGAACACAAGTTCCAGGAAATTGGTTTGGAGAGAGGTTGGGGTGATACCGCGGGGCGTGTGCTGGAGATGATCCAACTCCTTTTGGATCTTCTCGAGGCCCCTGATCCTTGCACCCTTGAGAAGTTCCTCGGGAGAGTCCCCATGGTGTTCAATGTTGTCATCCTTACTCCTCACGGATACTTTGCTCAAGACAACGTTTTGGGGTACCCCGACACCGGTGGCCAGGTTAGCCTTCTAAGCCTTGGTATAATTTGTGTATAAAACAAAGTTTATAAATGTATAAATCCTTTAACTTATTACTTATAGGTTGTATACATCTTGGATCAAGTCCGTGCCTTGGAGAACGAGATGCTCCTCCGTATCAAGCAGCAAGGACTCAACATTACCCCTCGAATCTTAATTGTAAGAGCTGTTGGTTATTAGTATTTTATGAACTTTTTATTTGTTTATTTTGGTTTCCTTTCAATATTTGATTTTGGTTGTTGTTGCTGATCAGATTACCAGACTTCTCCCTGACGCTGTTGGAACAACTTGTGGTCAACGAGTTGAAAAGGTATACGGAACGGAATACTCCGACATCCTCCGAGTACCCTTTAGAACAGAGAAGGGAATTGTACGTAGATGGATCTCGAGATTCGTAGTCTGGCCCTACTTGGAAACTTACACTGAGGTGAACTGTTGATCTTCTTGTATCTAATTGCTATAGATAATCTGTAATATTTACAAGTAAAAATCTCTTTGGCTTTAGGATGTTGCTCACGAAATTTCCAAAGAGTTGCAAGGCAAGCCCGATCTCATCATCGGAAACTATAGTGACGGCAACATTGTTGCCTCGTTGCTGGCTCACAAATTGGGAGTCACACAGGTTTTTAAGAGGCTAATTCACTTTGTTTTTCCTTTATCATGTTTCAGTATAATGGGTGATTATATTTCATTCTTTTGCAGTGTACCATTGCCCATGCTTTGGAGAAAACAAAGTACCCAGATTCCGATATTTACTGGAAGAAGCTAGAGGATAAATACCATTTCTCCTGCCAATTTACAGCTGATCTTATTGCAATGAACCATACAGATTTCATCATCACTAGTACTTTCCAAGAAATTGCAGGAAGGTATATATATATGTGTGTATATGTATTCTTGATATGCTTTTTTGTACAGTTTTAACACACAATATTATGATGACTTCAGCAAGGACACTGTTGGTCAATATGAGAGTCACACTGCTTTCACTCTTCCTGGTTTATACCGAGTCGTACACGGTATCGATGTATTTGATCCCAAATTCAACATCGTGTCTCCCGGTGCTGACATGAGCATATACTTCCCTTACACCGAGAAGAAGAGGAGGTTGAAGCATTTCCACCCCGAGATTGAAGACCTCCTTTATAGCAAAGTCGAGAACGAAGAACACTTGTAAGTACAACACACCAAAATGTTTTACCTACATGTTATTAGCAAATTCCTATTTGAACTAATGATTTGTTCATCGTTGTCCCAACAGATGTGTGCTAAATGACCGCAACAAGCCGATCCTATTCACGATGGCAAGGCTAGACCGTGTTAAGAACTTAACCGGACTCGTCGAGTGGTACGGCAAGAATGCAAAGCTGCGCGAGTTGGTTAACCTCGTAGTCGTAGGTGGAGACAGGAGAAAGGAATCCAAAGATTTAGAAGAGAAGGCCGAAATGAAGAAGATGTTTGAGCTGATAAAAACATACAAATTGAACGGTCAATTCAGATGGATATCATCGCAAATGAACCGAGTTAGGAATGGTGAGTTGTACCGCTACATTTGCGACACAAAAGGTGCCTTCGTACAACCAGCATTGTACGAAGCCTTTGGATTAACGGTTGTTGAAGCCATGACTTGCGGATTGCCAACATTTGCTACCTGCAAAGGTGGACCAGCTGAAATCATTGTCCACGGTAAATCTGGGTTCAACATTGATCCTTACCATGGTGATCAAGCTGCAGAAATCCTTGTTGATTTCTTCGACAAATGTAAAAAGGAACCATCTCACTGGAATGACATCTCTGAGGGTGGCTTGAAACGTATCCAGGAGAAGTAAGCTCACAAATTTTTGAATTACATACAAGTTAATAACATATAGTTTTTGGTTAATGTTTGTTGTAAGGATGTTGATTGTTTGGATTGTTGGAATCAGATACACATGGCAGATATATTCGGAGAGGCTATTAACATTGACGGGAGTGTATGGATTCTGGAAGCACGTTTCCAACCTTGACCGTCGCGAGAGCCGCCGTTACCTGGAGATGTTTTACGCTCTTAAGTACCGCAAGCTGGTAGATTGCATCATCACCATTTCCTTTGTTTGGCTTATGTTAGTCCAAGTGAAAGTTGCACACTGCTAACTACGTTGTTGCCATGTTTGTGTTAATTACAGGCTGAATCGGTTCCTCTGGCAGAGGAGGAGTAA null

***GhSus3Dt* D06** ATGGCAGAGCGTGTGATCACCCGAGTACACAGCCTCCGGGAGCGTCTGGATGACACCCTTATTGCCCATAGAAACGAGGTTTTGGCCTTGCTCACAAGGATCGAGGGTAAGGGAAAAGGGATTCTGCAACACCATCAAATTATCCTAGAGTTTGAAGCCATCCCTGAAGAAACCAGAAAGAAGCTCGCTGATGGAGCATTTTCTGAAATATTGAGATCCAGTCAGGAAGCGATCGTGTTGCCACCATGGGTTGCACTTGCAGTTCGTCCAAGGCCTGGTGTTTGGGAGTATATTAAAGTGAATGTCCACGCCCTTGTTGTTGAGGAACTCACTGTTGCTGAGTATCTTCACTTCAAGGAAGAGCTTGTTGATGGAAGTGCAAATGGCAATTTCGTTTTGGAATTGGATTTTGAGCCCTTCAATGCATCTTTCCCTCGCTCAACTCTTTCCAAGTCTATCGGTAATGGTGTCGAGTTCCTCAACCGCCACCTTTCGGCTAAATTGTTCCATGACAAGGAGAGCATGCACCCTTTGCTTGAATTCCTCAAAGTCCATTGCCACAAAGGAAAGAACATGATGTTGAATGACAGAATTCAAAACCTGAATTCCCTTCAACATGTATTGAGGAAGGCAGAAGAATATCTTGGTACACTGCCAGCTGAGACACCGTACGCTGAACTCGAACACAAGTTCCAGGAAATCGGTTTAGAGAGAGGTTGGGGTGATACCGCGGGGCGTGTGCTGGAGATGATCCAACTCCTTTTGGATCTTCTCGAGGCCCCTGATCCTTGCACCCTTGAGAAGTTCCTCGGGAGAGTCCCCATGGTGTTCAATGTTGTCATCCTTACTCCTCACGGATACTTTGCTCAAGACAACGTTTTGGGGTACCCCGACACCGGTGGCCAGGTTGTATACATCTTGGATCAAGTCCGTGCCTTGGAGAACGAGATGCTCCTCCGTATCAAGCAGCAAGGACTCAACATTACCCCTCGAATCTTAATTATTACCAGACTTCTCCCTGATGCCGTTGGAACAACTTGTGGTCAACGAGTCGAGAAGGTATACGGAACGGAATACTCAGACATCCTCCGAGTACCCTTTAGAACAGAGAAGGGAATTGTACGTAGATGGATCTCGAGATTCGAAGTCTGGCCCTACTTGGAAACTTACACTGAGGATGTTGCTCACGAAATTTCCAAAGAGTTGCAAGGCAAGCCCGATCTCATCATCGGAAACTATAGTGACGGTAACATTGTTGCCTCGTTGCTGGCTCATAAATTGGGAGTCACACAGTGTACCATTGCCCATGCTTTGGAGAAAACAAAGTACCCAGATTCCGATATTTACTGGAAGAAGCTCGAGGATAAATACCATTTCTCCTGCCAATTTACAGCTGATCTTATTGCAATGAACCATACAGATTTCATCATCACTAGTACTTTCCAAGAAATTGCAGGAAGCAAGGACACTGTTGGTCAATATGAGAGTCACACTGCTTTCACTCTTCCTGGTCTATACCGAGTCGTACACGGTATCGACGTATTTGATCCCAAATTCAACATCGTGTCTCCCGGTGCTGACATGAGCATATACTTCCCTTACACCGAGGAGAAGAGGAGGTTGAAGCATTTCCACCCCGAGATTGAAGACCTCCTTTACAGCAAAGTCGAGAACGAAGAACACTTATGTGTGCTAAACGACCGCAACAAGCCGATCCTATTCACAATGGCAAGGCTGGACCGTGTTAAGAACTTAACCGGACTCGTCGAGTGGTACGGAAAGAATGCAAAGCTTCGCGAGTTGGTTAACCTTGTAGTCGTAGGTGGAGACAGGAGAAAGGAATCCGAAGATTTAGAAGAGAAGGCCGAAATGAAGAAGATGTTTGAGCTGATAGAAACATACAAATTGAACGGCCAATTCAGATGGATATCATCGCAAATGAACCGAGTTAGGAACGGTGAGCTGTACCGCTACATTTGCGACACAAGGGGTGCCTTCGTACAACCAGCATTGTACGAAGCCTTTGGATTAACGGTTGTTGAAGCCATGACTTGCGGGTTGCCCACATTTGCTACCTGCAATGGTGGACCAGCTGAAATCATTGTCCACGGTAAATCTGGGTTCAACATTGATCCTTACCATGGTGATCAAGCTGCAGAGATCCTTGCCGACTTCTTCGACAAATGTAAAAAAGACCCATCTCACTGGAACGACATCTCTGAGGGTGGCTTGAAACGTATCCAGGAGAAATACACATGGCAGATATACTCGGAGAGGCTATTAACACTGACGGGAGTGTATGGGTTCTGGAAGCACGTTTCCAACCTTGACCGTCGCGAGAGCCGCCGTTACCTGGAGATGTTTTATGCTCTTAAGTACCGCAAGCTGGCTGAATCGGTTCCTCTGGCAGAGGAGTAA ATGGCAGAGCGTGTGATCACCCGAGTACACAGCCTCCGGGAGCGTCTGGATGACACCCTTATTGCCCATAGAAACGAGGTTTTGGCCTTGCTCACAAGGTAATTTAAATATAACCCCTTCAACGATTGCTTTTCCTTTTGGAAATGCTTAGTTTTGTTGTTTTCTATTGTAGGATCGAGGGTAAGGGAAAAGGGATTCTGCAACACCATCAAATTATCCTAGAGTTTGAAGCCATCCCTGAAGAAACCAGAAAGAAGCTCGCTGATGGAGCATTTTCTGAAATATTGAGATCCAGTCAGGTATAAAACAAACCCTTTTTGTGTTTTCTCTTATGGGTTTTATTGTGACACTAGTGTTGATTTTAGTGACATTTGTGTTGTAGGAAGCGATCGTGTTGCCACCATGGGTTGCACTTGCAGTTCGTCCAAGGCCTGGTGTTTGGGAGTATATTAAAGTGAATGTCCACGCCCTTGTTGTTGAGGAACTCACTGTTGCTGAGTATCTTCACTTCAAGGAAGAGCTTGTTGATGGAAGGTATCTTGAAAAATGTTCATTTTTTGTTTACTTCACTCGTAAATGCTCATTTTAACACTGTTTCTTTGATTTTTCTTGCAGTGCAAATGGCAATTTCGTTTTGGAATTGGATTTTGAGCCCTTCAATGCATCTTTCCCTCGCTCAACTCTTTCCAAGTCTATCGGTAATGGTGTCGAGTTCCTCAACCGCCACCTTTCGGCTAAATTGTTCCATGACAAGGAGAGCATGCACCCTTTGCTTGAATTCCTCAAAGTCCATTGCCACAAAGGAAAGGTATGCAACATAAAGCACCAGTTACTTGTGTTTTATAGTTGACATCTGCATATACTAAAACATAGATTTCTTAGCATAAGATTTAAATGATGGATTTAGTGTCCTGAATGTCTTAGTTGGTGTTTTTGCAGAACATGATGTTGAATGACAGAATTCAAAACCTGAATTCCCTTCAACATGTATTGAGGAAGGCAGAAGAATATCTTGGTACACTGCCAGCTGAGACACCGTACGCTGAACTCGAACACAAGTTCCAGGAAATCGGTTTAGAGAGAGGTTGGGGTGATACCGCGGGGCGTGTGCTGGAGATGATCCAACTCCTTTTGGATCTTCTCGAGGCCCCTGATCCTTGCACCCTTGAGAAGTTCCTCGGGAGAGTCCCCATGGTGTTCAATGTTGTCATCCTTACTCCTCACGGATACTTTGCTCAAGACAACGTTTTGGGGTACCCCGACACCGGTGGCCAGGTTAGCCTTCCAAGCCTTCGTATAATTTGTGTATAAAACTAAGTTTGTAAATGTATAAATCCTTCAACTTATTACTTATAGGTTGTATACATCTTGGATCAAGTCCGTGCCTTGGAGAACGAGATGCTCCTCCGTATCAAGCAGCAAGGACTCAACATTACCCCTCGAATCTTAATTGTAAGAGCTGTTGGTTATTAGTATTTTATGAACTTTTTATTGATTTATTTTGGTTTCCTTCCAATATCTGATTTTGGTTGTTGTTGCTGATCAGATTACCAGACTTCTCCCTGATGCCGTTGGAACAACTTGTGGTCAACGAGTCGAGAAGGTATACGGAACGGAATACTCAGACATCCTCCGAGTACCCTTTAGAACAGAGAAGGGAATTGTACGTAGATGGATCTCGAGATTCGAAGTCTGGCCCTACTTGGAAACTTACACTGAGGTGAACCATTGATCTTCTTGTATCCAATTCCTATAGATAATCTGTAATATTTACAAGTAAAAATCTCTTTGGCTTTAGGATGTTGCTCACGAAATTTCCAAAGAGTTGCAAGGCAAGCCCGATCTCATCATCGGAAACTATAGTGACGGTAACATTGTTGCCTCGTTGCTGGCTCATAAATTGGGAGTCACACAGGTTTTTTAGAGACTAATTCACTTTGTTTTCCCTTTATCATATTTCAGTATAATGGCTGATTTTATTTCATTCTTTTGCAGTGTACCATTGCCCATGCTTTGGAGAAAACAAAGTACCCAGATTCCGATATTTACTGGAAGAAGCTCGAGGATAAATACCATTTCTCCTGCCAATTTACAGCTGATCTTATTGCAATGAACCATACAGATTTCATCATCACTAGTACTTTCCAAGAAATTGCAGGAAGGTATATATATGTGTGTATATGTATTCTTGATATGCGTTTTTGTACAGTTTTAACACACAATATTGTGATGATTTCAGCAAGGACACTGTTGGTCAATATGAGAGTCACACTGCTTTCACTCTTCCTGGTCTATACCGAGTCGTACACGGTATCGACGTATTTGATCCCAAATTCAACATCGTGTCTCCCGGTGCTGACATGAGCATATACTTCCCTTACACCGAGGAGAAGAGGAGGTTGAAGCATTTCCACCCCGAGATTGAAGACCTCCTTTACAGCAAAGTCGAGAACGAAGAACACTTGTAAGTACAACACACCAAAATGTTTTACCTACATGTTATTAGCAAATTCCTATTTGAACTAATGATTTGTTCATCGTTGTCCCAACAGATGTGTGCTAAACGACCGCAACAAGCCGATCCTATTCACAATGGCAAGGCTGGACCGTGTTAAGAACTTAACCGGACTCGTCGAGTGGTACGGAAAGAATGCAAAGCTTCGCGAGTTGGTTAACCTTGTAGTCGTAGGTGGAGACAGGAGAAAGGAATCCGAAGATTTAGAAGAGAAGGCCGAAATGAAGAAGATGTTTGAGCTGATAGAAACATACAAATTGAACGGCCAATTCAGATGGATATCATCGCAAATGAACCGAGTTAGGAACGGTGAGCTGTACCGCTACATTTGCGACACAAGGGGTGCCTTCGTACAACCAGCATTGTACGAAGCCTTTGGATTAACGGTTGTTGAAGCCATGACTTGCGGGTTGCCCACATTTGCTACCTGCAATGGTGGACCAGCTGAAATCATTGTCCACGGTAAATCTGGGTTCAACATTGATCCTTACCATGGTGATCAAGCTGCAGAGATCCTTGCCGACTTCTTCGACAAATGTAAAAAAGACCCATCTCACTGGAACGACATCTCTGAGGGTGGCTTGAAACGTATCCAGGAGAAGTAAGCTCACAAATTTTTGAATTACATACAAGTCAATAACATATAGTTTTTGGTTAATGTTTGTTGTAAGGATGTTGATTGTTTGGATTGTTGGAATCAGATACACATGGCAGATATACTCGGAGAGGCTATTAACACTGACGGGAGTGTATGGGTTCTGGAAGCACGTTTCCAACCTTGACCGTCGCGAGAGCCGCCGTTACCTGGAGATGTTTTATGCTCTTAAGTACCGCAAGCTGGTAGATTGCATCATCTCCATTTCCTTTGTTTGGTTCATGTCAGTCCAAGTGAAAGTTGCAAACTGCTAAGTACATTGTTGCCATGTTTGTGTTAATTGCAGGCTGAATCGGTTCCTCTGGCAGAGGAGTAA null

***GhSus4At* A07** ATGGCTGAGCGTTTCGATGAGACCCTCACTTCCCACAGGAACGAGATTTTGCCCTTTCTTTTAAAGGAAGCAACAGTGTCACCTCCATGGGTTGCATTAGCTATTAGGCCAAGGCCTGGGGTTTGGCAATACATTAAAGTGAATGTCCACACTCTTGTTGTTGAGGACCTCACTGTTTCTAAATATCTTCATTTCAAAGAACAGCTTGTTGATGGAAGTGCAAATGGTAACTTTGTTTTGGAATTGGATTTTGAGCCCTTCAATGCCTCTTTCCCTCGCCCGACTCTTTCCAGCTCAATCGGTAACGGCGCTGAGTTCCTCAACCGTCACCTTTCAGCAACATTGTTCCACGATGACAACGAGAACATGCACCCTTTGCTCGAATTCCTCAAACTCCATTGCCTACCGAGACTCCGAATGCCGGATCTGAACATGATGTTGTTGAATGATAAAATCCAAAACTTGAATGCACTCCGACATGTTTTGAGAAAGGCCGAGGAGTATCTTGACACGTTGCCTTCCGAGATACTGTATGCCGAATTCAAACATGAGTTTCGGGAAATTGGTTTGGAGCCAGGTTGGGGTGATACAGCCGAGCATGTGCTTGAGATGATCCGAATCCTTTCGGACCTTCTCGAGGCACCTAACCCTTACAACCTAGAGAAGTTCCTTGGGAGAGTGCCTATGGTATTCAATGTTGTCATACTTTCCCCACATGGATACTTTGCTCAAGACAATGTTTTGGGGTACCCCGACACCGGTGGCCAAGTCGTTTACATCTTGGATCAAGTTCGAGCCTTGGAGAACGAGATGATCCACCGTATCAAACAACAAGGACTCGACATTACACCTCGTATCCTTATCATCACTAGGCTTCTCCCCGATGCTGTCGGAACAACGTGCAGTGAACGAGTTGAGAAAGTACATGGAACAGAGTATTCGGATATTCTTCGAGTACCCTTTAGAACAGAAACCGGAATCGTACGTCAATGGATCTCTAGATTCGAAGTTTGGCCCTACTTAGAAACTTACACTGAGGATGTTGCTAATGAAATCACGAAAGAGTTGCGAGGCAAACCTGATTTAATCATCGGAAACTATAGTGATGGTAACATCGTTGCCTCATTGCTGGCGCATAAGTTGGGAGTTACACAGTGTACGATCGCCCACGCTTTGGAGAAGACGAAGTATCCGAATTCGGATTTATACTGGAAGGAGCTTGAGGATAAATACCATTTCTCTTGCCAATTTACAGCTGATCTCATTGCAATGAACCATACTGATTTCATCATCACAAGTACTTTCCAAGAAATTGCAGGAAGCAAGGACAGTGTTGGACAATATGAGAGTCACGGGGCTTTCACTCTTCCTGGTCTTTACCGAGTCGTACACGGCATCGATGTGTTTGATCCCAAATTCAACATCGTCTCCCCTGGCGCTGACATGAGCACATTCTTCCCTTACACCAACGAGAAGCAGCGGTTGAAACATTTCCATCCTGAAATTGAAGACCTTCTTTACGGTAAAGTCGAGAATGAAGAATACATATGTGTGCTAAACGATCGTAATAAGCCAATCCTGTTCACAATGGCAAGGCTAGACCGTGTCAAGAACTTAACTGGCCTCGTTGAGTGGTATGGGAAGAACCCGAAGTTACGCAAGCTGGTTAACCTTGTCGTCGTAGCCGGCGACCGGAGAAAAGAATCAAAGGATTTGGAAGAGAAAGCTGAAATGAAGAAGATGTTTGAACTCATAGAAAAATACAAATTAAAAGGTCAATTTCGATGGATATCGTCCCAAATGAACCGTATTCGGAACGGTGAACTTTACCGTTACGTTTGCGACACAAAAGGCGCCTTCGTACAACCAGCATTGTACGAAGCCTTTGGATTGACAGTCGTTGAGGCAATGACTTGTGGTTTGCCTACATTCGCCACATGCAATGGAGGACCTGCCGAGATTATCGTCCATGGGAAATCCGGGTTCAACATTGATCCATACCAGGGCGACAAAGCCGCTGAGATCATCGTCGGTTTCTTCGAGAAATGTAAGAAAGACCCATCTCATTGGAATGAAATCTCCAATGGTGGATTGAAACGTATCCAAGAGAAATACACATGGAAGATTTACTCCGAGAGATTATTGACCCTGACCGGAGTTTATAGCTTTTGGAAGCACGTTTCCAAGCTTGACCGCCGCAAGAGCCGTCGTTATCTGGAGATGTTTTACGCACTAAATTACCGGAAGCTGGTTGAATCAGTTCCTCTAACAGGTGAGGAGTGA ATGGCTGAGCGTTTCGATGAGACCCTCACTTCCCACAGGAACGAGATTTTGCCCTTTCTTTTAAGGTAATTTGAATTACATCTTTCAAACCCAAAAGATTGCTCTTAAAATTCGCATTAAAAGTTGTAATNCTAAGTACAATTTTACCATTGTGATAGCTTATAACTTTAGAATTAGAAGGACTAGATCATAATTTATCATTTTGGGAGAGCAAAAATGTAATTTTATCATGTATTAACTTATAAATTTACAAATTTTAAAATGACTAAAGCACAATTTTTTTCATTTTTAAGGGGCAGAATCAGTGCCTCCCCTTGGTAAAATTAAAGCATAAGGACTAAATCCTAAATTTTAGTATGGTAAAGGGACCAAAACAATAATTTTCATTTTGTTGATTTTGGTCATATTTGTGTTATAGGAAGCAACAGTGTCACCTCCATGGGTTGCATTAGCTATTAGGCCAAGGCCTGGGGTTTGGCAATACATTAAAGTGAATGTCCACACTCTTGTTGTTGAGGACCTCACTGTTTCTAAATATCTTCATTTCAAAGAACAGCTTGTTGATGGAAGGTAACTTGTTTTATTTTAATCTTGCTTTCTTACCATTAACCTTTGTCTTAATTTCTTTTGTTTTGCAGTGCAAATGGTAACTTTGTTTTGGAATTGGATTTTGAGCCCTTCAATGCCTCTTTCCCTCGCCCGACTCTTTCCAGCTCAATCGGTAACGGCGCTGAGTTCCTCAACCGTCACCTTTCAGCAACATTGTTCCACGATGACAACGAGAACATGCACCCTTTGCTCGAATTCCTCAAACTCCATTGCCTACCGAGACTCCGAATGCCGGATCTGAACATGATGTTGTTGAATGATAAAATCCAAAACTTGAATGCACTCCGACATGTTTTGAGAAAGGCCGAGGAGTATCTTGACACGTTGCCTTCCGAGATACTGTATGCCGAATTCAAACATGAGTTTCGGGAAATTGGTTTGGAGCCAGGTTGGGGTGATACAGCCGAGCATGTGCTTGAGATGATCCGAATCCTTTCGGACCTTCTCGAGGCACCTAACCCTTACAACCTAGAGAAGTTCCTTGGGAGAGTGCCTATGGTATTCAATGTTGTCATACTTTCCCCACATGGATACTTTGCTCAAGACAATGTTTTGGGGTACCCCGACACCGGTGGCCAAGTCGTTTACATCTTGGATCAAGTTCGAGCCTTGGAGAACGAGATGATCCACCGTATCAAACAACAAGGACTCGACATTACACCTCGTATCCTTATCGTACGTGTTATTGATTACTAGTGTTGTTTATGAACTTTTTTAACCATGCTTTGTTTTATCAGATCACTAGGCTTCTCCCCGATGCTGTCGGAACAACGTGCAGTGAACGAGTTGAGAAAGTACATGGAACAGAGTATTCGGATATTCTTCGAGTACCCTTTAGAACAGAAACCGGAATCGTACGTCAATGGATCTCTAGATTCGAAGTTTGGCCCTACTTAGAAACTTACACTGAGGTGAACCATATACATACCGTTGATTTCACTTTGTCTCCTTCGGATATTTTTCTTACATGTCATTACATTATGTTTGCTTTAAGGATGTTGCTAATGAAATCACGAAAGAGTTGCGAGGCAAACCTGATTTAATCATCGGAAACTATAGTGATGGTAACATCGTTGCCTCATTGCTGGCGCATAAGTTGGGAGTTACACAGGTTCGTGATAAACGATCTTTTGTCGGTTCACCTTTGGTCGTATTTCAGATTGATTGACTCATTTTTTTAATCTCCTATATAGTGTACGATCGCCCACGCTTTGGAGAAGACGAAGTATCCGAATTCGGATTTATACTGGAAGGAGCTTGAGGATAAATACCATTTCTCTTGCCAATTTACAGCTGATCTCATTGCAATGAACCATACTGATTTCATCATCACAAGTACTTTCCAAGAAATTGCAGGAAGGTATACATACATACTTGTATATATATATGATGTATTTATGCACGTATATATATACGATGTGCTTCGTAAGTACTCGATTTGATATGTTTTTCAATATATGTGTATGATTTCAGCAAGGACAGTGTTGGACAATATGAGAGTCACGGGGCTTTCACTCTTCCTGGTCTTTACCGAGTCGTACACGGCATCGATGTGTTTGATCCCAAATTCAACATCGTCTCCCCTGGCGCTGACATGAGCACATTCTTCCCTTACACCAACGAGAAGCAGCGGTTGAAACATTTCCATCCTGAAATTGAAGACCTTCTTTACGGTAAAGTCGAGAATGAAGAATACATGTAAGTGTTCGTCATATCTTAGCAAAAACCCTAATTTGAAAACGATTTTACTTACACATCAAGCTATCATTTTTCCAACAGATGTGTGCTAAACGATCGTAATAAGCCAATCCTGTTCACAATGGCAAGGCTAGACCGTGTCAAGAACTTAACTGGCCTCGTTGAGTGGTATGGGAAGAACCCGAAGTTACGCAAGCTGGTTAACCTTGTCGTCGTAGCCGGCGACCGGAGAAAAGAATCAAAGGATTTGGAAGAGAAAGCTGAAATGAAGAAGATGTTTGAACTCATAGAAAAATACAAATTAAAAGGTCAATTTCGATGGATATCGTCCCAAATGAACCGTATTCGGAACGGTGAACTTTACCGTTACGTTTGCGACACAAAAGGCGCCTTCGTACAACCAGCATTGTACGAAGCCTTTGGATTGACAGTCGTTGAGGCAATGACTTGTGGTTTGCCTACATTCGCCACATGCAATGGAGGACCTGCCGAGATTATCGTCCATGGGAAATCCGGGTTCAACATTGATCCATACCAGGGCGACAAAGCCGCTGAGATCATCGTCGGTTTCTTCGAGAAATGTAAGAAAGACCCATCTCATTGGAATGAAATCTCCAATGGTGGATTGAAACGTATCCAAGAGAAGTAAGCTCTTTTTTGTTGTTACTTGTTACCTACGTTAACTATATAGTCTTTGCGGTAATTCTTTTTCCTTTTTTTTATTTACATTTTTGAAAATTTAGATACACATGGAAGATTTACTCCGAGAGATTATTGACCCTGACCGGAGTTTATAGCTTTTGGAAGCACGTTTCCAAGCTTGACCGCCGCAAGAGCCGTCGTTATCTGGAGATGTTTTACGCACTAAATTACCGGAAGCTGGTATTTCTCACGTCCATTTCTCCCTTTTTTTTTCAAGAGTCAAATTCTAGTTTTAGTCCTTGTACTATACTTAATTTTAAAATTTAATCATTATACTTTTAATTTCACATAATTTAATATTATTATTTAGTCTTAAATCACTAACACTTCGAAAAGATTCTGTTAGTAAGCTGATGTGGCTTTTTCTAAAAGAGAAAATTTAAATTTGAAAATAGATTTTTAATGCCATTTAAAAGGCTAAAATTATGGTTATAAAAAATACTTATTTTGCGCTAATTTGTCATTAAAAAAAAATTAGCCCGACTGAAGGAATTCTTTTAATAGTTTTAGTGATTTCAACTAAGATATAATATTAATAGGGTAAATTACGTGGACATTTTCCTTTTTCAGTCATTCAATCATGAAAAGTTATAAAATGGTCACTAAATTATTTAATTTTATCTTTTTTAGTCACCCAACTGGCTTGGATTTTGGGGTGTTTCCATCTTTACATTAGCCTGCTAGTGGTAAAAATAAAGACAAAATTGAATAGTTGAATGACCGTTTTATAACTTTTTATAGTTGGATGATCATTTTATAATTTTTCATAGTTGAGTGACCGAAAAAGGAAAAAAAATCCTAATTATGTGACCTTTATTGTAGTTTACCCAGATTAATAATAGGAGAAGAATCAAATTATGGCAAATAAAACTGACGTGGAATTAAAAAAATTCAAAATCTGAATTTTTTTATATAATTTAAGGGTTGAAATTATGGTTATTAAAATTCAAAATCACATTAGCTGACTAACAAAATTTCTTCATGGTGTTAGTCATTTGGACTAAATCATAACAACAATAATAATGGTAGGATCAAATTGTGCTAATTTAAAGTATATAAAAACTAAATCTCCAAATTAAATATAGTATAAGGACGAAAATTATAATTTAACTTATCATTTTATATGGTTCCTATATGAAAAGCTATTGTTAATGTTGTTGTTTTTTTTTTTTAATTTATTTCAGGTTGAATCAGTTCCTCTAACAGGTGAGGAGTGA null

***GhSus4Dt* D07** ATGGCTGAGCGTTTCGATGAGACCCTCACTCCCCACAGGAACGAGATTTTGCCCTTTCTTTTAAGGATTGAGGGCAAAGGGAAAGGAATACTGCAACACCATCAAATTGCCCTCCTCATTGAAGATTATAGAAAGAAGCTTGCTGATGGGGCATTTTATGAAATTTTGAGGGCTATTCAGGAAGCAATAGTGTTACCTCCATGGGTGGCATTAGCTATTAGGCCAAGGCCTGGGGTTTGGCAATACATTAAAGTGAATGTCCACACTTTTGTTGTTGAGGACCTCACTGTTTCTAAATATCTTCATTTCAAAGAACAGCTTGTTGATGGAAGTGCAAATGGTAACTTTGTTTTGGAATTGGATTTTGAGCCCTTCAATGCCTCTTTCCCTCGCCCGACTCTTTCCAACTCAATCGGTAACGGCGCCGAGTTCCTCAACCGTCACTTTTCAGCAACATTGTTCCATGACGACAACGAGAACATGCACCCTTTGCTTGAATTCCTCAAACTCCATTGCCTACCGAGACAACGAATGCCGAATCTGAACATGATGATGTTGAATGATAAAATCCAAAACTTGAATGCACTCCGACATGCTTTGAGAAAGGCCGAGGAGTATCTTGACACGTTGCCTTCCGAGACACTGTATGCCGAATTCGAACATGAGTTTCGGGAAATCGGTTTGGAGCCAGGTTGGGGTGATACAGCCGAGCATGTGCTTGAGATGATCCGAATCCTTTCGGACCTTCTCGAGGCACCTAACGCTTACAACCTAGAGAAGTTCCTTGGGAGAGTGCCTATGGTATTCAATGTTGTCATACTTTCCCCACATGGATACTTTGCTCAAGACAATGTTTTGGGGTACCCCGACACAGGTGGCCAAGTCGTTTACATCTTGGATCAAGTTCGAGCCTTGGAGAACGAGATGATCCAGCGTATCAAACAACAAGGACTCGACATTACACCTCGTATCCTAATCATCACTAGGCTTCTCCCCGATGCTGTCGGAACAACGTGCAGTGAACGAGTTGAGAAAGTACACGGAACAGAGTATTCGGATATTCTTCGAGTACCCTTTAGAACAGAAAACGGAATCGTACGTCAATGGATCTCTAGATTCGAAGTTTGGCCCTACTTAGAAACCTACACTGAGGATGTTGCTAATGAAATCACGAAAGAGATGCGATGCAAACCTGATTTAATCATCGGAAACTATAGTGACGGCAACATCGTTGCCTCATTGCTGGCGCATAAGTTGGGAGTTACACAGTGTACGATCGCCCACGCTTTGGAGAAGACGAAGTATCCGAATTCGGATTTATACTGGAAGGAGCTTGAGGATAAATACCATTTCTCTTGCCAATTTACAGCTGATCTCATTGCAATGAACCATACTGATTTCATCATCACAAGTACTTTCCAAGAAATTGCAGGAAGCAAGGACAGTGTTGGACAATATGAGAGTCACGCGGCTTTCACTCTTCCTGGTCTTTACCGTGTCGTACACGGCATCAATGTGTTCGATCCCAAATTCAATATCGTCTCCCCCGGGGCTGACATGAGCACATTCTTCCCTTACACCGACGAGAAACGGCGGTTGAAACATTTCCATCCTGAAATCGAAGACCTTCTTTATGGCAAAGTCGAGAATGAAGAATACATATGTGTGCTAAACGATCGTAATAAACCGGTCCTGTTCACGATGGCAAGGCTGGACCGTGTCAAGAACTTAACTGGCCTTGTTGAGTGGTATGGGAAGAACCCGAAGTTACGCGAGCTGGTTAACCTTGTCGTTGTAGCCGGCGACCGGAGAAAAGAATCAAAGGATTTGGAAGAGAAAGCTGAAATGAAGAAGATGTTTGAACTCATAGAAAAATACAAATTAAACGGTCAATTTCGATGGATATCGTCCCAAATGAACCGTATTCGGAATGGTGAACTTTACCGTTACATTTGCGACACAAAAGGAGCCTTCGTACAACCAGCATTATACGAAGCCTTTGGATTGACAGTCGTTGAGGCAATGACTTGTGGTTTGCCTACATTCGCCACCTGCAACGGCGGACCTGCCGAGATTATCGTCCATGGGAAATCCGGGTTCAACATTGATCCATACCAGGGCGACAAAGCCGCCGAGATCATCGTCAGTTTCTTCGAGAAATGTAAGAAAGACCCGTCTCATTGGAATGAAATCTCCGATGGTGGATTGAAACGTATCCAAGAGAAATACACATGGAAGATTTACTCCGAGAGATTATTGACCCTGACCGGAGTTTATAGCTTTTGGAAGCACGTTTCCAAGCTTGACCGCCACAAGAGCCGTCGTTATCTGGAGATGTTTTACGCACTAGAGTACCGGAAGCTGGTTGAATCAGTTCCTCTAACAGGTGAGGAGTGA ATGGCTGAGCGTTTCGATGAGACCCTCACTCCCCACAGGAACGAGATTTTGCCCTTTCTTTTAAGGTAATTTGAATTACATCTTTCAAACCCAAAAGATTGCTCTTAAAATTCGCATTAAAAGTTGTAATGCCTTTGGTTTTTATTGTAGGATTGAGGGCAAAGGGAAAGGAATACTGCAACACCATCAAATTGCCCTCCTCATTGAAGATTATAGAAAGAAGCTTGCTGATGGGGCATTTTATGAAATTTTGAGGGCTATTCAGGTAATAATAATAATAATAAGGTCAAATTCTGCTTTTGGTCTCTCAAGTTTCAAATATGCTCAAATTTGGTATTTACCCTCTAACTGTATAAAATTATTAGTTAGTAATAGTAATAGTAAAATTGTACTTTATTCCCGAAAAATGATAAGTTTTTTATTTACTCCCTCAAAAATTGTAAAGATATTAGCTAATACATTGATAAAATTGCATTTTAAATCATATTAAAAAATATAACTTAAATCCACCCTAAAAAATTTTACTCAATATTAAAGGCAACGTAAGAATTTTTTTTTTTTGGAGATGCCGAAATTGAATTAGAAATTCTTGTAAGAGTCTAAGTACAATTTTACCTTGTATTAGCTTATTATAACTTTAAATTTTTTAAAAGAACTAAATCATAATTTTATCATTTTAGTGAGCCAAACTACAATTTTACCACTAGCTTATAACTTTACAATTAGAAGGACTAGATCATAATTTATCATTTTGGGAGAGCAAAAATGCAATTTTATCATGTATTAACTTCTAAATTTACAAATTTTAAAAGGAAAAAAGCACAATTTTTTTTTCATTTTTAAGGGGCAGAGTCTGTGCGTCCCCCTTGGCAAAATTAAAGCATAAGGACTAAATCCTAAATTTTAGTATGGTAAAGGGACCAAAACCGTAATTTTCATTTTGTTGATTTTGGTCATATTTGTGTTATAGGAAGCAATAGTGTTACCTCCATGGGTGGCATTAGCTATTAGGCCAAGGCCTGGGGTTTGGCAATACATTAAAGTGAATGTCCACACTTTTGTTGTTGAGGACCTCACTGTTTCTAAATATCTTCATTTCAAAGAACAGCTTGTTGATGGAAGGTAACTTGTTTTATTTTAATCTTGCTTTCTTACCGTAAATCTTTGTCTTAATTTCTTTTGCTTTTGCAGTGCAAATGGTAACTTTGTTTTGGAATTGGATTTTGAGCCCTTCAATGCCTCTTTCCCTCGCCCGACTCTTTCCAACTCAATCGGTAACGGCGCCGAGTTCCTCAACCGTCACTTTTCAGCAACATTGTTCCATGACGACAACGAGAACATGCACCCTTTGCTTGAATTCCTCAAACTCCATTGCCTACCGAGACAACGAATGCCGAATCTGAACATGATGATGTTGAATGATAAAATCCAAAACTTGAATGCACTCCGACATGCTTTGAGAAAGGCCGAGGAGTATCTTGACACGTTGCCTTCCGAGACACTGTATGCCGAATTCGAACATGAGTTTCGGGAAATCGGTTTGGAGCCAGGTTGGGGTGATACAGCCGAGCATGTGCTTGAGATGATCCGAATCCTTTCGGACCTTCTCGAGGCACCTAACGCTTACAACCTAGAGAAGTTCCTTGGGAGAGTGCCTATGGTATTCAATGTTGTCATACTTTCCCCACATGGATACTTTGCTCAAGACAATGTTTTGGGGTACCCCGACACAGGTGGCCAAGTCGTTTACATCTTGGATCAAGTTCGAGCCTTGGAGAACGAGATGATCCAGCGTATCAAACAACAAGGACTCGACATTACACCTCGTATCCTAATCGTACGTGTTATTGATTACTAGTGTTGTTTATGAACTTTTTTAACCATGCATTGTTTTATCAGATCACTAGGCTTCTCCCCGATGCTGTCGGAACAACGTGCAGTGAACGAGTTGAGAAAGTACACGGAACAGAGTATTCGGATATTCTTCGAGTACCCTTTAGAACAGAAAACGGAATCGTACGTCAATGGATCTCTAGATTCGAAGTTTGGCCCTACTTAGAAACCTACACTGAGGTGAACCATATACGTAACGTTGATTTCACTTTGTCTCCTTCGGATATTTTTCTTACATGTCATTACATTATGTTTGTTTTAAGGATGTTGCTAATGAAATCACGAAAGAGATGCGATGCAAACCTGATTTAATCATCGGAAACTATAGTGACGGCAACATCGTTGCCTCATTGCTGGCGCATAAGTTGGGAGTTACACAGGTTCGTGATAAACGATCTTTTGTCAATTCATCTTCGGTCGTATTTCAGATTGATTGACTCATTTTTCTAATCTCCTATATAGTGTACGATCGCCCACGCTTTGGAGAAGACGAAGTATCCGAATTCGGATTTATACTGGAAGGAGCTTGAGGATAAATACCATTTCTCTTGCCAATTTACAGCTGATCTCATTGCAATGAACCATACTGATTTCATCATCACAAGTACTTTCCAAGAAATTGCAGGAAGGTATACATACATACATACATACATACATACATACATACATACATGTATATATATGATGTATTTATGTACGTATATATATACGATGTGCTTCGTAAGTACACGATTTGATATGTTTTTCAATATATGTGTATGATTTCAGCAAGGACAGTGTTGGACAATATGAGAGTCACGCGGCTTTCACTCTTCCTGGTCTTTACCGTGTCGTACACGGCATCAATGTGTTCGATCCCAAATTCAATATCGTCTCCCCCGGGGCTGACATGAGCACATTCTTCCCTTACACCGACGAGAAACGGCGGTTGAAACATTTCCATCCTGAAATCGAAGACCTTCTTTATGGCAAAGTCGAGAATGAAGAATACATGTAAGTGTTCGTCATATCTTAGCAAAAACCCTAATTCAAAAATGACTTTACTTACATATCTAGCTATCATTTTTCCAACAGATGTGTGCTAAACGATCGTAATAAACCGGTCCTGTTCACGATGGCAAGGCTGGACCGTGTCAAGAACTTAACTGGCCTTGTTGAGTGGTATGGGAAGAACCCGAAGTTACGCGAGCTGGTTAACCTTGTCGTTGTAGCCGGCGACCGGAGAAAAGAATCAAAGGATTTGGAAGAGAAAGCTGAAATGAAGAAGATGTTTGAACTCATAGAAAAATACAAATTAAACGGTCAATTTCGATGGATATCGTCCCAAATGAACCGTATTCGGAATGGTGAACTTTACCGTTACATTTGCGACACAAAAGGAGCCTTCGTACAACCAGCATTATACGAAGCCTTTGGATTGACAGTCGTTGAGGCAATGACTTGTGGTTTGCCTACATTCGCCACCTGCAACGGCGGACCTGCCGAGATTATCGTCCATGGGAAATCCGGGTTCAACATTGATCCATACCAGGGCGACAAAGCCGCCGAGATCATCGTCAGTTTCTTCGAGAAATGTAAGAAAGACCCGTCTCATTGGAATGAAATCTCCGATGGTGGATTGAAACGTATCCAAGAGAAGTAAGCTCTTTTGTGTTGTTACTTGTTACCTACGTTAACTATATAATCTTTGTGGTAATTCTTTTTCCTTCTTTTTTTTTTTTACATTTTTTAAAATTTAGATACACATGGAAGATTTACTCCGAGAGATTATTGACCCTGACCGGAGTTTATAGCTTTTGGAAGCACGTTTCCAAGCTTGACCGCCACAAGAGCCGTCGTTATCTGGAGATGTTTTACGCACTAGAGTACCGGAAGCTGGTATTTCTCACGTCCATTTCCTTTTTTTTTTTTCAAGAGTCAAATTCTAGTTTAAGTCCTTCTACTATACTCAATTTCAAAATTTAATCCTTATACTTTTAATTTCACATAATTTAATATTATTATTTAGTCTTAAATCACTAACACTTGGAAAAGATTTTGTTAGTAAGCTGATGTGGCTTTTTCTAAAAGAGAAAATTTAAATTTGAAAATAGATTTTTTATGCCATTTAAAAGGCTAAAATTATGGTTATAAAAAATACTTATTTATTAAAAAAAATTTTAGCCGACTGAAGGAATTCTTTTAATAGTTTTAGTGATTTCAACAAGATATAATATTAATAGGGTAAACTACGTCAATAGTAGTGGGCATTTTTCTTTTTCAGTCACTCAATTATGAAAAGTTATAAAATAGTCACTAAATTATTTAATTTTATCTTTTTAAGTCACCCAACTACCTTGGATTTTGGGGTGTTTCCAGCTTTACATTAGCCTGAGAGTGGTAAAAAGACAAAATTGAATAGTTGGATGATCATTTTATAACTTTTTATGGTTAAATGACCAAAAAAGAAAAAATCCTAATTGTGTAACCTTTATTGTAGTTTACCCAGATTAATAATAGGAGAAGAATCAAATTATGTCAAATAAAATTGACTTGAAATTAAAAAAAATTCAAAATCTGAATTTTTTTATATAATTTAAGAGTTGAAATTATGGTTATTAAAATACTTATTGTCTTATTAATTTGTCATTAGAGAAATCACATTAGCTGACTAACAAAATTTCTTCTTGGTGTTAGTGATTTGGACTAAATCATAACAACAATAATAATGGTAGGATCAAATTGTGCTAATTTAAAGTATATAAAAACTAAATCTCCAAATTAAATATAGTATAAGGACGAAAATTATAATTTTTCATTTTATATGGTTTCTATATGAAAAGCTATTGTTAATGGGATTTTTTCTTTTTTGTAATTTATTTCAGGTTGAATCAGTTCCTCTAACAGGTGAGGAGTGA null

***GhSus5Dt* D06** ATGGCTTCAATCAGTGTTTGTGAGCGTTTGGGTGAATCTCTAGCTACTCATCCACAGCAGGCAAAGTCTATCTTGTCAAGGATTGAAAGCCTCGGAAAGGGTATTCATAAGTCTCAAAAGCTGCTCTCGGTTCTCGATAAAGAGGCCGGAAATCAAGCACTTGATGGGATGGTGGTGGAGGTCCTCAGGTCCACTCAGGAAGCTGTAGTGTCGTCTCCATTGGTTGCCCTTGCCATTCGTTCAGCTCCTGGAGTTTGGGAGTACATTGCTGTGGAGGTCCAAAAGCTTTTTGTGGAGGAAATGCCCGTTGCTGAGTATCTACGGTTGAAGGAAGAACTTGTTGATGGAAGCTCCAATGGCGAGTTTATGTTGGAATTGGACTTTGGTGCATTCAATAATTCTGTTCCTCGTCCATCTCTTTCAAAGTCCATTGGTAATGGCATGGACTTCCTCAACCGCCACCTTTCTGCCAAGCTATTTCAAGACAAGGAGAACTTGAACTTGTTGCTTGAATTTCTCCAAATTCACTGCCAGAAGGGAAAGGGTATGCTGTTGAATGACAGAATCCAAGATGTGAATTCCCTCCAACATGCATTAAGGAAGGCCGAGGAGTATCTGACTCCTCTATCCTCGGATACCCCGTACTCAGTTTTCGAGAAAAGGTTTCTGGGGATTGGTTTGGAGAAGGGATGGGGTGATAATGCTGAGCATGTTCTTGAGATGATCCATCTTCTATTGGATCTCCTTCAGGCACCTGATCCTGTCGCACTTGAAAGCTTCCTTGGAAGAATCCCATTGGTCGCCAATGTTGTAATCATGACTCCCCATGGATACTTTGCCCAAGACAATGTTTTGGGATACCCTGACACAGGTGGTCAGGTTGTTTATATCTTAGATCAAGTTCGTGCCTTGGAGGAAGAGTTGCTTCATCGTTTCAAGCTGCAAGGACTCGACATTACCCCACGTATCCTAGTCATTACTCGGCTCCTCCCTGATGCTGTAGGAACAACTTGCGGTCAGCGTCTTGAGAAAGTGTATGGAACCAAGTATTCTGATATTCTTCGGGTACCCTTCAGAACTGAGAAGGGAATTGTACGTCCATGGATCTCACGATTCAAAGTCTGGCCTTACCTGGAGACTTACACTAAGGATGTTGCAGCTGAGATCACCAAAGAGTTCCAGGGCAAGCCTGATCTGATTGTTGGGAACTACAGTGATGGAAACATTGTTGCTTCTTTATTGGCACATAAGTTTGATGTTACACAGTGCACTATTGCTCATGCACTCGAGAAGACGAAATACCCGGATTCAGACATTAACTGGAAACAGCTTGAGGATAAGTATCACTTCTCCTGTCAGTTTACTGCTGATCTTATTGCTATGAACCATACTGATTTTATCATCACCAGCACCTTCCAAGAGATTGCTGGAAGCAAGGACACTCTCGGCCAATACGAGAGTCACATTGCTTTCACTCTTCCAGGGCTCTACCGCGTTGTTGATGGGATCGATGTTTTTGATCCCAAATTCAATATTGTCTCCCCTGGTGCTGATATGAGCATATACTTCCCTTACACGGAAGAGAAGCGGAGGTTGAAGAAGTTCCACCCGGAGATTGAAGAGCTTCTTTACAGCCCTGTTGAGAATACAGAGCACTTATGTGTACTAAAAGACCGCAACAAGCCGATTCTGTTTACCATGGCAAGGTTGGACCGAGTGAAGAACTTAACTGGGCTCGTAGAGTTCTATGCCAAGAACAGCCGGCTGAGGGAACTGGTTAACTTGGTTGTAGTAGGTGGAGATAGGAGAAAGGAATCCAAGGACTTAGAAGAAAAGGCTGAAATGAAGAAGATGTATGAACTTATCGAAAAATACAAGTTGAATGGACAATTCAGATGGATATCGTCCCAGATGAACCGAGTGAGAAATGGTGAACTCTATCGTTATATTTGCGACACCAAGGGAGCATTCGTTCAGCCTCCTATATACGAGGCTTTTGGCTTGACTGTTGTTGAGGCAATGACCTGTGGACTACCAACATTTGCAACATGTTACGGGGGCCCTGCTGAGATTATAGTTCACGGAAAATCGGGGTTCAACATCGATCCTTATAACGGTGATTTGGCTGCCGAGACCCTTGCCAATTTCTTCGAGAAGTGCAAAGCGGATCCATCTTATTGGGATGAGATCTCCCAGGGAGGGTTGAAACGCATACAGGAGAAGTATACATGGCAGATTTACTCCGAGAAGCTATTGACTCTCACCGGAGTTTATGGCTTTTCGAAACATGTAGCTTACCAGGAGCAACGTGGGCGCAAGCGTTACATTGAAATGTTGCATGCATGGATGTATAACAATCGGGTCAAGACTGTTCCACTAGCTGTTGAGTAA ATGGCTTCAATCAGTGTTTGTGAGCGTTTGGGTGAATCTCTAGCTACTCATCCACAGCAGGCAAAGTCTATCTTGTCAAGGTATAATGTAAATAAGTGCTGCCTTCAAATTTCTGGTCTTTCTTCGTTATTTTTTATTACATTGTTTATATATGTCATATTGCAGGATTGAAAGCCTCGGAAAGGGTATTCATAAGTCTCAAAAGCTGCTCTCGGTTCTCGATAAAGAGGCCGGAAATCAAGCACTTGATGGGATGGTGGTGGAGGTCCTCAGGTCCACTCAGGTCACAATAAAACAAACCCTTTCTTTGGTTATGTTTCATGATGAGAATATTGGACTGATATGTTACCGTTATCTAATAGGAAGCTGTAGTGTCGTCTCCATTGGTTGCCCTTGCCATTCGTTCAGCTCCTGGAGTTTGGGAGTACATTGCTGTGGAGGTCCAAAAGCTTTTTGTGGAGGAAATGCCCGTTGCTGAGTATCTACGGTTGAAGGAAGAACTTGTTGATGGAAGGTAGGTAGTTTTCTCTTACAGCTCCTGGTTTATATTATGTTTGAGTTTATGTTGAACAATGCTCAATGATGTGGTTCAGCTCCAATGGCGAGTTTATGTTGGAATTGGACTTTGGTGCATTCAATAATTCTGTTCCTCGTCCATCTCTTTCAAAGTCCATTGGTAATGGCATGGACTTCCTCAACCGCCACCTTTCTGCCAAGCTATTTCAAGACAAGGAGAACTTGAACTTGTTGCTTGAATTTCTCCAAATTCACTGCCAGAAGGGAAAGGTAAAAGTTTCACTTTCACTATTTGTTTTTCCTTTCTGTATACATCAAAACTTAAGAATCGTTCAATGTTATGCCACAGGGTATGCTGTTGAATGACAGAATCCAAGATGTGAATTCCCTCCAACATGCATTAAGGAAGGCCGAGGAGTATCTGACTCCTCTATCCTCGGATACCCCGTACTCAGTTTTCGAGAAAAGGTTTCTGGGGATTGGTTTGGAGAAGGGATGGGGTGATAATGCTGAGCATGTTCTTGAGATGATCCATCTTCTATTGGATCTCCTTCAGGCACCTGATCCTGTCGCACTTGAAAGCTTCCTTGGAAGAATCCCATTGGTCGCCAATGTTGTAATCATGACTCCCCATGGATACTTTGCCCAAGACAATGTTTTGGGATACCCTGACACAGGTGGTCAGGTTGTTTATATCTTAGATCAAGTTCGTGCCTTGGAGGAAGAGTTGCTTCATCGTTTCAAGCTGCAAGGACTCGACATTACCCCACGTATCCTAGTCGTGAGTATCTGAATGTTAATTCAGTTTCGATCATTATGTTTAATGCTTAATTTGGCTGTTTGTGTACCAGATTACTCGGCTCCTCCCTGATGCTGTAGGAACAACTTGCGGTCAGCGTCTTGAGAAAGTGTATGGAACCAAGTATTCTGATATTCTTCGGGTACCCTTCAGAACTGAGAAGGGAATTGTACGTCCATGGATCTCACGATTCAAAGTCTGGCCTTACCTGGAGACTTACACTAAGGATGTTGCAGCTGAGATCACCAAAGAGTTCCAGGGCAAGCCTGATCTGATTGTTGGGAACTACAGTGATGGAAACATTGTTGCTTCTTTATTGGCACATAAGTTTGATGTTACACAGGTTTGTTAAATACCAATTAATTGAACAAGTTCCTTGCTGTATTTTGATGTTCCCCGTGCAATCTAAAAACTTGGTTTTCAATGTTTTATGCAGTGCACTATTGCTCATGCACTCGAGAAGACGAAATACCCGGATTCAGACATTAACTGGAAACAGCTTGAGGATAAGTATCACTTCTCCTGTCAGTTTACTGCTGATCTTATTGCTATGAACCATACTGATTTTATCATCACCAGCACCTTCCAAGAGATTGCTGGAAGGTAATCTATCTCCCACTTTACTACATTTGGTTCCCTTGTACGTTGTAAATGTTGCCACCTATTTGAGTCCCTAACAATGTTTTCATTCCATGATTTTCAGCAAGGACACTCTCGGCCAATACGAGAGTCACATTGCTTTCACTCTTCCAGGGCTCTACCGCGTTGTTGATGGGATCGATGTTTTTGATCCCAAATTCAATATTGTCTCCCCTGGTGCTGATATGAGCATATACTTCCCTTACACGGAAGAGAAGCGGAGGTTGAAGAAGTTCCACCCGGAGATTGAAGAGCTTCTTTACAGCCCTGTTGAGAATACAGAGCACTTGTGAGATTTTGGTCCTATTTTTACACCCTTTTCACTAGATTTTGTGTTCAGTGTTAACAATGTCTTAAATTGTTGTCTCACAGATGTGTACTAAAAGACCGCAACAAGCCGATTCTGTTTACCATGGCAAGGTTGGACCGAGTGAAGAACTTAACTGGGCTCGTAGAGTTCTATGCCAAGAACAGCCGGCTGAGGGAACTGGTTAACTTGGTTGTAGTAGGTGGAGATAGGAGAAAGGAATCCAAGGACTTAGAAGAAAAGGCTGAAATGAAGAAGATGTATGAACTTATCGAAAAATACAAGTTGAATGGACAATTCAGATGGATATCGTCCCAGATGAACCGAGTGAGAAATGGTGAACTCTATCGTTATATTTGCGACACCAAGGGAGCATTCGTTCAGCCTCCTATATACGAGGCTTTTGGCTTGACTGTTGTTGAGGCAATGACCTGTGGACTACCAACATTTGCAACATGTTACGGGGGCCCTGCTGAGATTATAGTTCACGGAAAATCGGGGTTCAACATCGATCCTTATAACGGTGATTTGGCTGCCGAGACCCTTGCCAATTTCTTCGAGAAGTGCAAAGCGGATCCATCTTATTGGGATGAGATCTCCCAGGGAGGGTTGAAACGCATACAGGAGAAGTATACATGGCAGATTTACTCCGAGAAGCTATTGACTCTCACCGGAGTTTATGGCTTTTCGAAACATGTAGCTTACCAGGAGCAACGTGGGCGCAAGCGTTACATTGAAATGTTGCATGCATGGATGTATAACAATCGGGTAAAACAATCATGTCTATTATTATTATTACATCAAACATCATTTTACTCTTTCATTGAACTGAAATTTTCATGTTTCTGAATGTTTGTTTAATTGTGTGCTTTACAGGTCAAGACTGTTCCACTAGCTGTTGAGTAA null

***GhSus6At* A08** ATGGCGAATCCTAACCTCGGTCGAAGTCCTAGCATGAGGGACCGCGTGGAGGACACGCTTTCTGCTCATCGTAACGAGCTCGTTGCTCTTCTCTCCAGGTACGTGGCGCAGGGAAAAGGAATACTGCAACCGCATACTTTGATCGATGAACTGGAAAATGTTGTAGGCGACGACAAAGCTAGAGAGAAGCTAAGTGATGGTCCCTTTAGCGAAGTCCTTAAATCTGCACAGGAGGCCATAATTCTGCCTCCATACGTGGCTATAGCAGTTCGCCCAAGACCTGGTGTTTGGGAATACGTACGAGTCAATGTTCATGAACTCAGCGTGGAGCAACTGGATGTGTCCGAATATCTTCGCTTCAAAGAAGCACTTGCAGATGTGGGGGAGGACAACCATTTTGTGCTTGAGCTTGATTTTGAGCCATTCAATGCATCCTTTCCTCGGCCCAACCGCTCTTCATCCATTGGCAATGGCGTCCAATTCCTCAACCGTCACCTTTCTTCAAACATGTTCCGTAACAAAGATTCTTTGGAGCCTTTACTTAATTTCCTGAGAGCCCACAAATATAAAGGGCATGCATTGATGTTGAATGATCGGATACAAAGTATACCCCGACTTCAAGCTGCTTTGGCTAAGGCAGAAGATCATCTTGCTAAGCTTTCACCTGATGCACCATATTCTGAGTTTGAATACGAATTACAAGGAATGGGTTTTGAGAGAGGATGGGGAGATACTGCAGCTCATGTTCTGGAGACGATGCATCTTCTCTTGGACATCCTTCAGGCACCTGATCCCTCTATATTAGAGACATTCCTTGGGAGAGTGCCTATGGTGTTTAATGTTGTCATTCTGTCTCCACATGGATACTTCGGGCAAGCAAATGTATTAGGTTTGCCTGACACTGGTGGTCAGGTTGTTTATATACTGGACCAAGTGCGAGCCTTAGAGAATGAAATGCTTCTAAGGATAAAGAGGCAAGGACTTGATATTACTCCCAGAATTCTTATTGTGACCAGGTTAATACCTGATGCGAAGGGAACTAGTTGCAATCAGCGGCTGGAAAGAGTCAGTGGGACAGAGCATACTCATATTCTGCGAGTTCCTTTTAGGTCAGAACATGGAGTTCTTCGTAAATGGATATCAAGGTTTGATGTATGGCCTTATCTGGAGACTTATGCGGAGGACGTCGCAAGTGAAATTGCAGCAGAGTTACAGGGTATTCCAGATTTTATTATAGGAAACTACAGTGACGGAAACCTGGTTGCATCTTTGTTGGCTTACAAAATGGGCGTCACACAGTGTACCATTGCCCATGCTTTGGAGAAAACAAAATATCCAGATTCAGATATATATTGGAAAAAGTTTGACGAGAAATATCACTTCTCTTGTCAGTTTACTGCCGACTTAATAGCCATGAATAATGCTGATTTTATTATCACCAGCACATACCAAGAGATTGCAGGAACGAAGAATACTGTTGGTCAGTATGAGAGCCATACTGCTTTTACTCTTCCAGGGCTGTATCGAGTGGTTCATGGCATTGATGTTTTTGATCCGAAGTTCAATATTGTATCTCCCGGGGCAGATATGTGCATTTATTTTCCATACTCTGAAAAGGAAAAGAGACTTACGGCACTGCATGGCTCAATAGAAGAATTGTTGTTTGATCCTAAGCAGAATGATGAACACATTGGTACTTTGAGTGATCGGTCAAAGCCCTTAATCTTTTCCATGGCAAGGCTGGATCGAGTTAAAAACATGACTGGATTGGTAGAGTTGTATGCTAAGAATAACAAGCTGAGGGAATTAGCAAACCTCGTTGTTGTTGCTGGTTACATTGATGTAAAGAAGTCCAAAGACAGAGAAGAGATAGCAGAAATTGAAAAGATGCATGACCTTATGAAAGAGTATAAATTAGATGGTCAATTTCGTTGGATAGCAGCCCAAACAAATCGAGCACGCAATGGTGAGCTCTATCGCTACATAGCTGACTCAAAGGGTATATTTGTTCAGCCTGCATTCTACGAAGCCTTTGGACTTACAGTGGTGGAAGCCATGACATGTGGCCTTCCAACGTTTGCCACATTGCATGGTGGTCCTGCAGAGATTATTGAACATGGTATATCAGGGTTCCATATTGATCCATACCACCCTGACCAGACCGCTGAACTCCTGGCAACTTTCTTTGAACGTTGCAAGGAGGATCCAAGCCACTGGACTAAAATATCTGATGGAGGGCTTAAGAGGATTTATGAAAGGTATACGTGGAAAATTTATTCTGAAAGGCTGATGACATTGGCTGGAGTATATGGTTTCTGGAAGTACGTCTCAAAACTCGAGAGGCGTGAGACCCGAAGATATCTTGAAATGTTCTACATCCTCAAGTTCCGTGAATTGGTAAAATCTGTTCCCTTGGCCAGTGATGATTAA ATGGCGAATCCTAACCTCGGTCGAAGTCCTAGCATGAGGGACCGCGTGGAGGACACGCTTTCTGCTCATCGTAACGAGCTCGTTGCTCTTCTCTCCAGGTGCGCTCTTCCGCGATTTTACAGTTCCTATCACCTTGTTCTCTTTTTCTTTTCCGGATTTTAAATTTTGCAGTCTCTATTTGGCTTATGGGAAAACGATGAAAAGTAAAAGGAAAGGAAAGGAAGATATGTTACTGAAGTTTAGGGTTGTGGAATTTTGAAGGTACGTGGCGCAGGGAAAAGGAATACTGCAACCGCATACTTTGATCGATGAACTGGAAAATGTTGTAGGCGACGACAAAGCTAGAGAGAAGCTAAGTGATGGTCCCTTTAGCGAAGTCCTTAAATCTGCACAGGTCATTTCCCCCCCTTAATTATCGTATTAATTTTTAAGATATTGTTAGCTTTTATATATTAAAAAGGATAAACGACTTTTTTAGATATTTATTTAATTTTCGTCCGTGATTCATGATGCAGTAAATAAAGTATCTAATACTTTTTAAATGCTTAATTGTTTTTTCAGCTAAATTATTTTTGTACTATTTAGTTTCAGTTGAACTTTTAGGTTTTTTTTTTTTTTTAAGATTCTGTTTTCCTAAATTTACTCTAATTCAATTTACAAATGAATTTCTGGATGTGTTTTATGTTCTTTCCTTACCTCGATGCTGCGTCTTTAAAGGAGTGTATGTAAACTGAAGTTTTAGTAGTAATGCTATTTAATTTTCACGCCTAACTTTCAGCCTTTGTACTAGGTAAGCTTTTAACGTCTGTCAAATGGAATATGTACGGTCTCGATTATGTTATTGTATTTAATATACTAAATTGTTGGGGTTTTTTTTAATAACTACAGGAGGCCATAATTCTGCCTCCATACGTGGCTATAGCAGTTCGCCCAAGACCTGGTGTTTGGGAATACGTACGAGTCAATGTTCATGAACTCAGCGTGGAGCAACTGGATGTGTCCGAATATCTTCGCTTCAAAGAAGCACTTGCAGATGTGGGGTAAGCTTATTTTCTGTTCTTTTTAAGTTGCGGTTATTGTTTCTTTGAAGTCAATAGATTCATAGTACTTGATTATGAATGTTCTACCTCATAACTCATTGTGATTTTTCTTTTTACACATTGCGTAGGGAGGACAACCATTTTGTGCTTGAGCTTGATTTTGAGCCATTCAATGCATCCTTTCCTCGGCCCAACCGCTCTTCATCCATTGGCAATGGCGTCCAATTCCTCAACCGTCACCTTTCTTCAAACATGTTCCGTAACAAAGATTCTTTGGAGCCTTTACTTAATTTCCTGAGAGCCCACAAATATAAAGGGCATGTAAGTATTCATCAGAGGGGTTTCCTTTAAACCCCTTACCATAGTTGCAACTTCACTAGCTATGTGTTATGTTCCTTTAGATTTTAGGAAAAGAAAATTTTGCCTTGTTTTATGTGTGGTGACGTATTAACAGCTTTGGAAAGGAATATGTCGGAGTATCTATTTTGTGATGAAATTCTCATAACAAGATAAACTAATGAAGGTTTTTCTGTAGGCATTGATGTTGAATGATCGGATACAAAGTATACCCCGACTTCAAGCTGCTTTGGCTAAGGCAGAAGATCATCTTGCTAAGCTTTCACCTGATGCACCATATTCTGAGTTTGAATACGAGTGAGTTTCTATTTGTGTTCTATTTGTATATACATTTAATACATACATATAGATTTGTTTCAAGGAGTCTATCAGCATATTGGTAGTGGCAGAGCAATAAAGTTGTTGACATCTCTGTCTGATTTTTCCTTGTTTTATGAATGCAGATTACAAGGAATGGGTTTTGAGAGAGGATGGGGAGATACTGCAGCTCATGTTCTGGAGACGATGCATCTTCTCTTGGACATCCTTCAGGCACCTGATCCCTCTATATTAGAGACATTCCTTGGGAGAGTGCCTATGGTGTTTAATGTTGTCATTCTGTCTCCACATGGATACTTCGGGCAAGCAAATGTATTAGGTTTGCCTGACACTGGTGGTCAGGTTTGTAGTGAATCTGATGTTTAACGTGTAGGATAATTTATATGTAAATATCTTCATTTAAGATCTCCATTTTTCTTATTTAATGTGTAGGTTGTTTATATACTGGACCAAGTGCGAGCCTTAGAGAATGAAATGCTTCTAAGGATAAAGAGGCAAGGACTTGATATTACTCCCAGAATTCTTATTGTAAGTATACTAACTAGATGCCATATTGCTTTATTTTGTTTTCAACACTTATATTTTGCCTATGTAAATTTGACACATTTTTTCTTCTTGTTTAAGGTGACCAGGTTAATACCTGATGCGAAGGGAACTAGTTGCAATCAGCGGCTGGAAAGAGTCAGTGGGACAGAGCATACTCATATTCTGCGAGTTCCTTTTAGGTCAGAACATGGAGTTCTTCGTAAATGGATATCAAGGTTTGATGTATGGCCTTATCTGGAGACTTATGCGGAGGTAAATCATTTTCCCTTTCAAAGTTTCAAAGGCTATTGATTTTCCTTTTTTTTTTTGAAGAAAAAAACATTAGATTTTTCAACAATCACAACAAATTTAGGAGGACGTTTTCTATGTACTTCTCTATAGGGTCTATCTTTCTTTCTTTCTTAATCTTCTTTCATAACTTGCTTTCCTATTTGTATAACTAATCAGGACGTCGCAAGTGAAATTGCAGCAGAGTTACAGGGTATTCCAGATTTTATTATAGGAAACTACAGTGACGGAAACCTGGTTGCATCTTTGTTGGCTTACAAAATGGGCGTCACACAGGTTTGTCCCTCCACTGTCCCAATTCCCTTTTCCCTCCACTCTTTGCCTTTTACTTTTGCTGAAAGCTTTGTTACTAAATCTTCAATGTGTAAATTACACTTTCTGCAGTGTACCATTGCCCATGCTTTGGAGAAAACAAAATATCCAGATTCAGATATATATTGGAAAAAGTTTGACGAGAAATATCACTTCTCTTGTCAGTTTACTGCCGACTTAATAGCCATGAATAATGCTGATTTTATTATCACCAGCACATACCAAGAGATTGCAGGAACGTAAGTTCTCTCCTTTTACTTTAGAATATACTATTTATTTACTGGACTGGTATTATTACCGTTCATTTTGGTATGGATATGCCATTATCATTATTTTATCTTAATATGTTTTAAGAACATTGTTTATCTTAATAAACTTCAAGTCATCTGCTGTCCTATAGTAAGGAGATGGATCCCTTGGGTAAAATATGAGCGAAGTGTTGGTTTTCAATTTATATCAATTGCTTATCACACTTTTATTTCTTTTTTGGGCTTCAGGAAGAATACTGTTGGTCAGTATGAGAGCCATACTGCTTTTACTCTTCCAGGGCTGTATCGAGTGGTTCATGGCATTGATGTTTTTGATCCGAAGTTCAATATTGTATCTCCCGGGGCAGATATGTGCATTTATTTTCCATACTCTGAAAAGGAAAAGAGACTTACGGCACTGCATGGCTCAATAGAAGAATTGTTGTTTGATCCTAAGCAGAATGATGAACACATGTGAGTTTCTTCATTCATTGTTTGTTGTACCTTATACATTAATGTCTAGAAGAGCATAATCTTTGAATTAATAACATTTTGATCTGCTTGTTATACCATAATGTTTTATTTGCTGAAGCACTTATAAATATTCTTTTGTCCTTTTTTTTTTCCTTTCTCTTTTTGCTATTTATTAAATATAATCAAGAATGTTGGTTGCCAAGAAAAGGAAGTTACATTTTGATATCTACTTTCAAGAACCTTAGTAGTTGACATCAAATTGAAACTAGACATTTTGCCTTGTTGCAAAATTTTGCAGCACAAGTCTATGATGAAATTTTCTTGTTTTGCCAAGTTTGTGATGCAAATTTCATCTTCAATGTGCAGTGGTACTTTGAGTGATCGGTCAAAGCCCTTAATCTTTTCCATGGCAAGGCTGGATCGAGTTAAAAACATGACTGGATTGGTAGAGTTGTATGCTAAGAATAACAAGCTGAGGGAATTAGCAAACCTCGTTGTTGTTGCTGGTTACATTGATGTAAAGAAGTCCAAAGACAGAGAAGAGATAGCAGAAATTGAAAAGATGCATGACCTTATGAAAGAGTATAAATTAGATGGTCAATTTCGTTGGATAGCAGCCCAAACAAATCGAGCACGCAATGGTGAGCTCTATCGCTACATAGCTGACTCAAAGGGTATATTTGTTCAGGTATGTGCTCCGTAGCAATATGATTCTATTGTGTTACAACCTCCTGAGATTTCTAATAAATTAACCCCTTCTCGTTTTTCAGCCTGCATTCTACGAAGCCTTTGGACTTACAGTGGTGGAAGCCATGACATGTGGCCTTCCAACGTTTGCCACATTGCATGGTGGTCCTGCAGAGATTATTGAACATGGTATATCAGGGTTCCATATTGATCCATACCACCCTGACCAGACCGCTGAACTCCTGGCAACTTTCTTTGAACGTTGCAAGGAGGATCCAAGCCACTGGACTAAAATATCTGATGGAGGGCTTAAGAGGATTTATGAAAGGTGCGTGTTTCTTCTAATTTTTACATTTAGAGTTTAGACTTCCATTCTCTCTACCATCGAAGCAGAGCTTAAGCAAAGGTTTTTGTACTTCAGGTATACGTGGAAAATTTATTCTGAAAGGCTGATGACATTGGCTGGAGTATATGGTTTCTGGAAGTACGTCTCAAAACTCGAGAGGCGTGAGACCCGAAGATATCTTGAAATGTTCTACATCCTCAAGTTCCGTGAATTGGTAAGTGGACTTTGTTCCCTCTTAATGGCCTGATGTTGGCAGCTCTACCTCTGAATCTCGTTGCGGATTATAATAGAACTTAAGCATGCTTGTGTTCTTTTTTCAGGTAAAATCTGTTCCCTTGGCCAGTGATGATTAA null

***GhSus6Dt* D08** ATGGCGAATCCTAAGCTCGGTCGAAGTCCTAGCATGAGGGACCGCGTGGAGGACACGCTTTCTGCTCATCGTAACGAGCTCGTTGCTCTTCTCTCCAGGTACGTGGCGCAGGGAAAAGGAATACTGCAACCGCATACTTTGATCGATGAACTGGAAAATGTTGTAGGCGACGACAAAGCTAGAGAGAAGCTAAGTGATGGTCCCTTTAGCGAAGTCCTTAAATCTGCACAGGAGGCCATAATTCTGCCTCCATACGTGGCTATAGCAATTCGCCCAAGACCTGGTGTTTGGGAATACGTACGAGTCAATGTTCACGAACTCAGCGTGGAGCAACTGGATGTGTCCGAATATCTTCGCTTCAAAGAAGCACTTGCAGATGTGGGGGAGGACAACCATTTTGTGCTTGAGCTTGATTTTGAGCCATTCAATGCATCCTTTCCTCGGCCCAACCGCTCTTCATCCATTGGCAATGGCGTTCAATTCCTCAACCGTCACCTGTCTTCAAACATGTTCCGTAACAAAGATTCTTTGGAGCCTTTACTTAATTTCCTGAGAGCCCACAAATATAAAGGGCATGCATTGATGTTGAATGATCGGATACAGAGTATACCCCGACTTCAAGCTGCTCTGGCTAAGGCAGAAGATCATCTTGCTAAGCTTTCATCTGATGCACCATATTCTGAGTTTGAATACGAATTACAAGGAATGGGTTTTGAGAGAGGATGGGGAGATACTGCAGCTCATGTTCTGGAGACGATGCATCTTCTCTTGGACATCCTTCAGGCTCCTGATCCCTCTATATTAGAGACATTCCTTGGGAGAGTGCCTATGGTGTTTAATGTTGTCATTCTGTCTCCACATGGATACTTTGGGCAAGCAAATGTATTAGGTTTGCCCGACACTGGTGGTCAGGTTGTTTATATACTGGACCAAGTGCGAGCCTTAGAGAATGAAATGCTTCTAAGGATAAAGAGGCAAGGACTTGATATTACTCCCAGAATTCTTATTGTGACCAGGTTAATACCTGATGCGAAGGGAACTAATTGCAATCAGCGGCTGGAAAGAGTCAGTGGGACAGAGCATACTCATATTCTGCGAGTTCCTTTTAGGTCAGAACATGGAGTTCTTCGTAAATGGATATCAAGGTTTGATGTATGGCCTTTTCTGGAGACTTATGCGGAGGACGTAGCAAGTGAAATTGCAGCAGAGTTGCAGGGTATTCCAGATTTTATTATAGGAAACTACAGTGACGGAAACCTGGTTGCATCTTTGTTGGCTTACAAAATGGGTGTCACACAGTGTACCATTGCCCATGCTTTGGAGAAAACAAAATATCCAGATTCAGATATATATTGGAAAAAGTTTGACGAGAAATATCACTTCTCTTGTCAGTTCACTGCTGACTTAATAGCCATGAATAATGCTGATTTTATTATCACCAGCACATACCAAGAGATTGCAGGAACGAAGAATACTGTTGGTCAGTATGAGAGCCATACTGCTTTTACTCTTCCAGGGCTGTATCGAGTGGTTCATGGCATTGATGTTTTTGATCCGAAGTTCAATATTGTATCTCCCGGGGCAGATATGTGCATTTATTTTCCATACTCTGAAAAGGAAAAGAGACTGACGGCACTGCATGGCTCAATAGAAGAATTGTTGTTTGATCCTAAGCAGAATGATGAACACATTGGTACTTTGAGTGATCGGTCAAAGCCCTTAATCTTTTCCATGGCAAGGCTGGATCGAGTTAAAAACATGACTGGATTGGTAGAGTTGTATGCTAAGAATAACAAGCTGAGGGAATTAGCAAACCTCGTTGTTGTTGCTGGTTACATTGATGTAAAGAAGTCCAAAGACAGAGAAGAGATAGCAGAAATTGAAAAGATGCATGACCTTATGAAAGAGTATAAATTAGATGGTCAATTTCGTTGGATAGCAGCCCAAACAAATCGAGCACGCAATGGTGAGCTCTATCGCTATATAGCTGACTCAAAGGGTATATTTGTTCAGCCTGCATTCTACGAAGCCTTTGGACTTACGGTGGTGGAAGCCATGACATGTGGCCTTCCTACGTTTGCCACATTGCATGGTGGTCCTGCAGAGATTATCGAACATGGTATATCAGGGTTCCATATTGATCCATACCACCCTGACCAGACCGCTGAACTCCTGGCAACTTTCTTTGAACGTTGCAAGGAGGACCCAAGCCACTGGACTAAAATATCTGATGGAGGGCTTAAGAGGATTTATGAAAGGTATACATGGAAAATTTATTCTGAAAGGCTGATGACATTGGCTGGAGTATATGGTTTCTGGAAGTACGTTTCAAAACTCGAGAGACGTGAGACCCGAAGATATCTTGAAATGTTCTACATCCTCAAGTTCCGTGAATTGGTAAAATCTGTTCCCTTGGCCAGTGATGATTAA ATGGCGAATCCTAAGCTCGGTCGAAGTCCTAGCATGAGGGACCGCGTGGAGGACACGCTTTCTGCTCATCGTAACGAGCTCGTTGCTCTTCTCTCCAGGTGCGCTCTTCCGCGATTTTACAGTTCCTATCACCTTGTTCTATTTTCTTTTCCGAATTTTAAATTTTGTAGTCTCTATTTGGCTTATGGGAAAACGATGAAAAGTAAAAGGAAAGGAAAGGAAGATATGTTACTGAAGTTTAGGGTTGTGGAATTTTGAAGGTACGTGGCGCAGGGAAAAGGAATACTGCAACCGCATACTTTGATCGATGAACTGGAAAATGTTGTAGGCGACGACAAAGCTAGAGAGAAGCTAAGTGATGGTCCCTTTAGCGAAGTCCTTAAATCTGCACAGGTCATTTCCCCCCCTTAATTATCGTATTAATTTTTAAGATATTGTTAGCTTTTATATATTAAAAAGGATAAACGACTTTTTTAGATGTTTATTTAATTTTCGTCCGTGATTCATGAGGCAGTAAATAAAGTATCTAATACTTTTTAAATGCTTAATTGTTTTTTCAGCTAAAATATTTTTGTACTATTTAGTTTCAGTTGAACTTTTAGGTTTTTTTTTTAATTTTTTTATTTTTAAGATTCTGTTTTCCTGAATTTACTCTAATTCAATTTACAAATGAATTTCTGGATGTGTTTTATGTTCTTTCCTTACCTCGATGCTGCGTCTTTAAAGGAGTGTACGTAAACTGAAGTTTAGTAGTAATGCTATTTAATTTTCACGCCTAACTTTCAGCCTTTGTACTAGGTAAGCTTTTAACGTCTGCCAAATGGAATATGTACGGTCTCGATTATGTTATTGTATTTAATATACTAAATTGTTGGGTTTTTCTTTTTTCTTTTTTAATAACTACAGGAGGCCATAATTCTGCCTCCATACGTGGCTATAGCAATTCGCCCAAGACCTGGTGTTTGGGAATACGTACGAGTCAATGTTCACGAACTCAGCGTGGAGCAACTGGATGTGTCCGAATATCTTCGCTTCAAAGAAGCACTTGCAGATGTGGGGTAAGCTTATTTTCTGTTCTTTGTAAGTTGCGTTTATTGTTTCTTTGAAGTCAATAGATTCAAAGTACTTGGTTATGTATGTCCTACCTCATAACTCATTGAGATTTTTCTTTTTACACATTGCGTAGGGAGGACAACCATTTTGTGCTTGAGCTTGATTTTGAGCCATTCAATGCATCCTTTCCTCGGCCCAACCGCTCTTCATCCATTGGCAATGGCGTTCAATTCCTCAACCGTCACCTGTCTTCAAACATGTTCCGTAACAAAGATTCTTTGGAGCCTTTACTTAATTTCCTGAGAGCCCACAAATATAAAGGGCATGTAAGTAATCATCAGAGGGGTTTCCTTTAAACCCCTTACCATAGTTGCAACTTCACTAGTTATGTGTTATGTTCCTTTAGATTTTAGGAAAAGAAAATTTTGCCTTGTTTTATGTGTGGTGACGCATTTACAGCTTTGGAAAGGCATATGTGGGAGTATCTATTTTGTGATGAAATTCTCATAACAAGATAAACTAATGAAGCTTTCTCTGTAGGCATTGATGTTGAATGATCGGATACAGAGTATACCCCGACTTCAAGCTGCTCTGGCTAAGGCAGAAGATCATCTTGCTAAGCTTTCATCTGATGCACCATATTCTGAGTTTGAATACGAGTGAGTTTCTATTTCTGTTCTATTTGTATATAGATTAAATACATACATATAGATTTGTTTCAAGGAGTCTATCAGCATATTGGTAGTGGCAGAGCAATAAAGTTGTTGACATCTCTGTCTGTTTTTCCTTGTTTTATGAATGCAGATTACAAGGAATGGGTTTTGAGAGAGGATGGGGAGATACTGCAGCTCATGTTCTGGAGACGATGCATCTTCTCTTGGACATCCTTCAGGCTCCTGATCCCTCTATATTAGAGACATTCCTTGGGAGAGTGCCTATGGTGTTTAATGTTGTCATTCTGTCTCCACATGGATACTTTGGGCAAGCAAATGTATTAGGTTTGCCCGACACTGGTGGTCAGGTTTGTAGTGAATCTGATATTTAACGTGTAGGATAATGTATATGTAACTATCTTCATTTAAGATCTCCATTTTTCTTATTTAATGTGTAGGTTGTTTATATACTGGACCAAGTGCGAGCCTTAGAGAATGAAATGCTTCTAAGGATAAAGAGGCAAGGACTTGATATTACTCCCAGAATTCTTATTGTAAGTATACTGACTAGATGCCGGATTGCTTTATTTTGTTTTCAACACTTATATTTTGCCTATGTAAATTTGACACATTTTTCCTTGTTGTTCAAGGTGACCAGGTTAATACCTGATGCGAAGGGAACTAATTGCAATCAGCGGCTGGAAAGAGTCAGTGGGACAGAGCATACTCATATTCTGCGAGTTCCTTTTAGGTCAGAACATGGAGTTCTTCGTAAATGGATATCAAGGTTTGATGTATGGCCTTTTCTGGAGACTTATGCGGAGGTAAATCATTTTCCCTTTCAAAGTTTCAAAGGCTATTGATTTTTTTTTTTTGAAGAGAAAATTTTCAACAATCACAACAAATTTAGGAGGACGTTTTCTATGTACTTCTCTATAGGTTCTATCTTTCTTTCTTTCTTAATCTTCTGTCATAACTTGCTTTCCTATTTGTATATCTAATCAGGACGTAGCAAGTGAAATTGCAGCAGAGTTGCAGGGTATTCCAGATTTTATTATAGGAAACTACAGTGACGGAAACCTGGTTGCATCTTTGTTGGCTTACAAAATGGGTGTCACACAGGTTTGTCCCTCCCCTGTCCCAAGTCCCTTTCCCCTCCACTCTTTGCCTTTCACTTTTGCTGAAAGCTTTGTTACTAAATCTTCAATGTGTAAATTACACTTTTTGCAGTGTACCATTGCCCATGCTTTGGAGAAAACAAAATATCCAGATTCAGATATATATTGGAAAAAGTTTGACGAGAAATATCACTTCTCTTGTCAGTTCACTGCTGACTTAATAGCCATGAATAATGCTGATTTTATTATCACCAGCACATACCAAGAGATTGCAGGAACGTAAGTTCTCTCCTTTTACTGTAGAATATACTATTTATTTACTGGACTAGTATTATTACCGTTCATTTTGGTATGGACATGCCATTATCATTATTTTATCTTAATATGTTTTAAGAACATTGTTTATCTTAATATACTTCAAGTCAGCTGCTTTCCTATAGTAGGAGATGGATCCCTTGGGTAAAATATGAGCGAAGTGTTGTTTCACTTTATATCAATTGCTTGTCACACTTTTCTTTCTTTTTTGGCTTCAGGAAGAATACTGTTGGTCAGTATGAGAGCCATACTGCTTTTACTCTTCCAGGGCTGTATCGAGTGGTTCATGGCATTGATGTTTTTGATCCGAAGTTCAATATTGTATCTCCCGGGGCAGATATGTGCATTTATTTTCCATACTCTGAAAAGGAAAAGAGACTGACGGCACTGCATGGCTCAATAGAAGAATTGTTGTTTGATCCTAAGCAGAATGATGAACACATGTGAGTTTCTTCATTCATTATTTGTTGTACCTTATACATTAATGTCTAGAAGAGCATAATCTTTGAATTAGTAACAGTTTGATCTGCTTGTTCTACCATAATGTTTTATTTGCTGAAGCACTTATAAATATTCTTTTTCCTTTTTTTTTTCTTTCTCTTTTTTCTATTTATTAAATATAATCAAGAATGTTGGTTGCCAAGAGAAGGAAGTTACATTTTGATATCTACTTTCAAGAACCTTAGTAGTTGACATCAAATTGAAACTAGACATTTTGCCTTGTTACAAAATTTTGCAGCACAAGTCTATGATGAAAGTTTCTTGTTTTGCCAAGTTTGTGATGCAAATTTCATCTTCAATGTGCAGTGGTACTTTGAGTGATCGGTCAAAGCCCTTAATCTTTTCCATGGCAAGGCTGGATCGAGTTAAAAACATGACTGGATTGGTAGAGTTGTATGCTAAGAATAACAAGCTGAGGGAATTAGCAAACCTCGTTGTTGTTGCTGGTTACATTGATGTAAAGAAGTCCAAAGACAGAGAAGAGATAGCAGAAATTGAAAAGATGCATGACCTTATGAAAGAGTATAAATTAGATGGTCAATTTCGTTGGATAGCAGCCCAAACAAATCGAGCACGCAATGGTGAGCTCTATCGCTATATAGCTGACTCAAAGGGTATATTTGTTCAGGTATGTGCTCCATAGCAATACGATTCTATCGTGTTACAACCTCCTGAGATTTCTAATAAATTAACCCCTTCTCGTTTTTCAGCCTGCATTCTACGAAGCCTTTGGACTTACGGTGGTGGAAGCCATGACATGTGGCCTTCCTACGTTTGCCACATTGCATGGTGGTCCTGCAGAGATTATCGAACATGGTATATCAGGGTTCCATATTGATCCATACCACCCTGACCAGACCGCTGAACTCCTGGCAACTTTCTTTGAACGTTGCAAGGAGGACCCAAGCCACTGGACTAAAATATCTGATGGAGGGCTTAAGAGGATTTATGAAAGGTGCGTGTTTCTTCTAATTTACATTTAGAGTTTGGACTTCCATTCTCTCTACCATCGATGCGGAGCTTAAGCAAAGGTTTTTGTACTTCAGGTATACATGGAAAATTTATTCTGAAAGGCTGATGACATTGGCTGGAGTATATGGTTTCTGGAAGTACGTTTCAAAACTCGAGAGACGTGAGACCCGAAGATATCTTGAAATGTTCTACATCCTCAAGTTCCGTGAATTGGTAAGTGGACTTTGTTCCCTCTTTTAGAGTTAATGGCCTGATGTTGGCAACTCTACTTCTGAATCTCGTTGCGGATTATAATAAAACTTAAGCATGCTTGTGTTCTTTTTCAGGTAAAATCTGTTCCCTTGGCCAGTGATGATTAA null

***GhSus7At* A07** ATGACGTCTACATCGACCGGGAAGCTTAGTGACTCCATAGCTTACAACATACGCAATGCCTTGAAGCAGAGCCAGTCTTACATGAAACGTTGCTTTTCTAAGTACATGGAGAAAGGAAAAAGGATTTTGAAAGCCCATGAATTGAGGGATGAATTTGAAAAAGTAATGGATGATAAAAATGAGACCTTGGGCACCATGTTTTCTTCAGCTCAGGTGTTTTATAATTTGCAGGAAGCGGTTGTTACTCCACCTTATGTTACCTTTGCCGTAAGACCGACTCCGGGATGTTGGGAGTTTGTTAAGGTGAACTCCGTTGATCTCTCCGACGTCAAACAAATATCCTCCGCCGAGTACTTGAAACTCAAAGAGACGACCGCCGATGAGAATTGGTCGAAAGATGAAAATGCATTAGAGGTGGATTTTGAAGCATTTGATTTCTCGATGCCAAAATTAACATTGGCTTCTTCTATTGGAAAAGGACTTAATTTTGTGTCAAAGTACATTACTTCTAAACTAAGTGGATCTGTGGATAATGCCCAGCCCCTTGTAGATTACTTACTCTCACTCGAATATCAAGGAGAGAAACTTATGATAAACGAGATACTTAACACAGCAGCAAAGCTTCAATTGGCTCTTATAGTAGCTGAAGTTTCCCTCTCAGATCTTCCTCGGGATACCCCATACCAGAGTATTGAGCTAAGGTTCAAGGAGTGGGGATTCGAGAGAGGGTGGGGTGACACGGTTGAAAGAGTGCATGAAACGATACGGTCACTCTCGGAAGTGTTGCAAGCACCTGATCCACAGAATTTGGAGAAGCTTTTTAGCAAACTTCCCACCATATTCAAGGTTGTAATCTTCTCTCCTCATGGATATTTTGGACAATCAGATGTGCTTGGTTTGCCAGACACTGGTGGACAGGTTGTTTATATTTTGGATCAAGTGAGGGCCATGGAGGAAGAATTGGTTCTCAAAATCAAATCCCAAGGCCTCAATATTAAGCCTCAAATCTTAGTGGTCACAAGACTCATACCTGATGCCCGAGGAACTAAGTGCAACCAAGAGTGGGAGCCCGTCATCGGCACCAAATACAGTCAGATCCTCCGAGTGCCTTTCAAGACTGAAACTGGTATCCTACGCCGATGGGTTTCGCGTTTCGACATTTATCCTTATCTTGAGACGTTTGCTCAGGATGTTACATCCAAGATCTTGGATGCAATGGAGGGTAAACCAGACCTTATTATTGGAAACTACACTGATGGGAACTTAGTATCATCTCTAGTAGCTAGCAAACTCGGGATAACACAGGCTACGATTGCGCATGCTTTAGAGAAGACGAAATACGAGGATTCAGACATCAAGTGGAAGGAACTTGACCCAAAGTATCATTTTTCATGTCAATTCATTGCTGATACAATTGCAATGAATGCTGCAGATTTCATTATAGCAAGCACATACCAGGAGATTGCAGGGAGCAAAGAGAGACCTGGACAATATGAGAGCCATGCTGCATTTACACTCCCAGGGCTCTGTAGAGTTGTTTCAGGCATCAATGTTTATGACCCTAAGTTCAACATCGCTGCTCCCGGTGCCGATCAATCTGTGTATTTCCCGTATACGGAGACCGGAAAACGATTCACATCATTTCATCCTGCAATTGAAGAACTTCTGTACAGTAAAGTGGATAATGATGAACACATTGGTTATCTAGCAGACAGGAAGAAACCTATAATTTTCTCAATGGCAAGACTGGATACAGTGAAGAACTTGACTGGATTAACTGAATGGTACGGTAAAAACAAAAGGCTAAGAAGCTTGGTCAACCTTGTAATAGTAGGAGCCTTCTTTAATCCCTCTAAATCAAAAGATAGAGAAGAAGTGGCTGAAATAAAAAAGATGCACGCACTCATAGAGAAATACCAACTCAAGGGCCAAATCCGATGGATAGCCGCGCAGACAGACCGTAACCGAAACGGCGAACTCTACCGTTGCATTGCCGATACAAAGGGTGCATTTGTTCAGCCAGCTTTATATGAAGCATTCGGTCTTACAGTGATCGAAGCAATGAACTGCGGTTTGCCGACCTTCGCAACCAATCAAGGAGGCCCTGCCGAGATCATCGTGGACGGAGTTTCCGGTTTCCATATTAATCCCACAAATGGAGATGAATCAAGCAACAAAATTGCTGATTTCTTTGAGAAATGCAAAACCAATCCTGCATATTGGAATCAGTTCTCAGCCGATGGATTGAAACGCATAAATGAATGCTATACCTGGAAAATATATGCAAACAAGGTATTGAATATGGGATGCATGTATAGGTTTTGGAAACAATTGAACAAAGATCAGAAACAAGCTAAACAGAGATACATTCAAGCATTTTATAATCTAATGTTCAGGAATCTGGTAAAAAATGTTCCTTTAGCAAGTGATGAAACTCAGCAACCAGACTCAAAGCCAGCAGCCAAACCACAACCTACACCGAGGCATGTCTAA ATGACGTCTACATCGACCGGGAAGCTTAGTGACTCCATAGCTTACAACATACGCAATGCCTTGAAGCAGAGCCAGTCTTACATGAAACGTTGCTTTTCTAAGTACATGGAGAAAGGAAAAAGGATTTTGAAAGCCCATGAATTGAGGGATGAATTTGAAAAAGTAATGGATGATAAAAATGAGACCTTGGGCACCATGTTTTCTTCAGCTCAGGTAAGTTTATCACATGCAAAAAAAGAAATGGAAAATGACGTTTTTTGAAAGATTATGAGGTGTTTTATAATTTGCAGGAAGCGGTTGTTACTCCACCTTATGTTACCTTTGCCGTAAGACCGACTCCGGGATGTTGGGAGTTTGTTAAGGTGAACTCCGTTGATCTCTCCGACGTCAAACAAATATCCTCCGCCGAGTACTTGAAACTCAAAGAGACGACCGCCGATGAGAATTGGTATGTCAATTTATGCTATGGTATTGTATCTTCAAAAGGTGTATGTGTTTATGTCCGTGTGTGTGTATTTAAGTGTTTAATGAATACTACAGGTCGAAAGATGAAAATGCATTAGAGGTGGATTTTGAAGCATTTGATTTCTCGATGCCAAAATTAACATTGGCTTCTTCTATTGGAAAAGGACTTAATTTTGTGTCAAAGTACATTACTTCTAAACTAAGTGGATCTGTGGATAATGCCCAGCCCCTTGTAGATTACTTACTCTCACTCGAATATCAAGGAGAGGTATATCTATATATATAAATATATGCATATGTACATATATGTACGATAAAATGCAAATTAATGGTTGTTTTCTCAACCTGCAGAAACTTATGATAAACGAGATACTTAACACAGCAGCAAAGCTTCAATTGGCTCTTATAGTAGCTGAAGTTTCCCTCTCAGATCTTCCTCGGGATACCCCATACCAGAGTATTGAGCTAAGGTAAAAACAGAATAGAAAAAGATATGGTTCTATCATGCTTGATGATTGCTTAGTATGTGTTTAAATTTTGTTGCAGGTTCAAGGAGTGGGGATTCGAGAGAGGGTGGGGTGACACGGTTGAAAGAGTGCATGAAACGATACGGTCACTCTCGGAAGTGTTGCAAGCACCTGATCCACAGAATTTGGAGAAGCTTTTTAGCAAACTTCCCACCATATTCAAGGTTGTAATCTTCTCTCCTCATGGATATTTTGGACAATCAGATGTGCTTGGTTTGCCAGACACTGGTGGACAGGTTGTTTATATTTTGGATCAAGTGAGGGCCATGGAGGAAGAATTGGTTCTCAAAATCAAATCCCAAGGCCTCAATATTAAGCCTCAAATCTTAGTGGTGAATACAAATTTCTCCCCAATAGTAGCAATTTACAGTTAGTTTCTAGCCTGAATGGTGCTTGATGAATGGTAATATTTAAGCATAATAGGTCACAAGACTCATACCTGATGCCCGAGGAACTAAGTGCAACCAAGAGTGGGAGCCCGTCATCGGCACCAAATACAGTCAGATCCTCCGAGTGCCTTTCAAGACTGAAACTGGTATCCTACGCCGATGGGTTTCGCGTTTCGACATTTATCCTTATCTTGAGACGTTTGCTCAGGCATGTGTAGATCTCTCTACTCATCCATCTGCTTCCATTTTGGTCTTACTAACTACCCACTTTTATTTGGATATCATTAGGATGTTACATCCAAGATCTTGGATGCAATGGAGGGTAAACCAGACCTTATTATTGGAAACTACACTGATGGGAACTTAGTATCATCTCTAGTAGCTAGCAAACTCGGGATAACACAGGTGTCTGTTGAAATTACACACACACACACACACACACACATGCTAATTTCTAACAAGTATTTCTATGTAGGCTACGATTGCGCATGCTTTAGAGAAGACGAAATACGAGGATTCAGACATCAAGTGGAAGGAACTTGACCCAAAGTATCATTTTTCATGTCAATTCATTGCTGATACAATTGCAATGAATGCTGCAGATTTCATTATAGCAAGCACATACCAGGAGATTGCAGGGAGGTTAGAGAATTAGCCATATCGAACTCCAACTGTTCATTTTTCTTGAAGTACCAGATCCAATATTGCTCACTTGTAACTTATTTTACCATGATTTGTGACATTGAATTCAATCATGTTGTTTGCAGCAAAGAGAGACCTGGACAATATGAGAGCCATGCTGCATTTACACTCCCAGGGCTCTGTAGAGTTGTTTCAGGCATCAATGTTTATGACCCTAAGTTCAACATCGCTGCTCCCGGTGCCGATCAATCTGTGTATTTCCCGTATACGGAGACCGGAAAACGATTCACATCATTTCATCCTGCAATTGAAGAACTTCTGTACAGTAAAGTGGATAATGATGAACACATGTAAGTGAAGGTCTATTGAATTATATTTTGCTTCAAATCACTTGGGATTATTCAACATCTTACTGAATTATTTTGCTTCAAAACAGTGGTTATCTAGCAGACAGGAAGAAACCTATAATTTTCTCAATGGCAAGACTGGATACAGTGAAGAACTTGACTGGATTAACTGAATGGTACGGTAAAAACAAAAGGCTAAGAAGCTTGGTCAACCTTGTAATAGTAGGAGCCTTCTTTAATCCCTCTAAATCAAAAGATAGAGAAGAAGTGGCTGAAATAAAAAAGATGCACGCACTCATAGAGAAATACCAACTCAAGGGCCAAATCCGATGGATAGCCGCGCAGACAGACCGTAACCGAAACGGCGAACTCTACCGTTGCATTGCCGATACAAAGGGTGCATTTGTTCAGCCAGCTTTATATGAAGCATTCGGTCTTACAGTGATCGAAGCAATGAACTGCGGTTTGCCGACCTTCGCAACCAATCAAGGAGGCCCTGCCGAGATCATCGTGGACGGAGTTTCCGGTTTCCATATTAATCCCACAAATGGAGATGAATCAAGCAACAAAATTGCTGATTTCTTTGAGAAATGCAAAACCAATCCTGCATATTGGAATCAGTTCTCAGCCGATGGATTGAAACGCATAAATGAATGGTAATAAAACAAGTTACAACCATGGCTGCTCATTTCTTTTGCAATATTAAAGCGCTGACTTGGTTTTTTTTTTTGGCTTGTAGCTATACCTGGAAAATATATGCAAACAAGGTATTGAATATGGGATGCATGTATAGGTTTTGGAAACAATTGAACAAAGATCAGAAACAAGCTAAACAGAGATACATTCAAGCATTTTATAATCTAATGTTCAGGAATCTGGTATATATATATATCTTTCTCTATTACTGGTTCACCTTTCATTGCTTTGATTTTATATCTGTTTATTAATATTAAAAAAACATTTTACAGGTAAAAAATGTTCCTTTAGCAAGTGATGAAACTCAGCAACCAGACTCAAAGCCAGCAGCCAAACCACAACCTACACCGAGGCATGTCTAA null

***GhSus7Dt* D07** ATGACGTCTACATCGACCGGGAAGCTTAGTGACTCCATAGCTGACAACATACGCAATGCCTTGAAGCAGAGCCAGTCTTACATGAAACGTTGCTTTTCTAAGTACATGGAGAAAGGAAAAAGGGTTTTGAAAGCCCATGAATTGAGGGATGAATTTGAAAAAGTAATGGATGATAAAAATGAGACCTTGGGTACCATGTTTTCTTCAGCTCAGGAAGCGGTTGTTACTCCACCTTATGTCACCTTTGCCGTAAGACCGACTCCGGGATGTTGGGAGTTTGTTAAGGTGAACTCCGTCGATCTCTCCGACGTCAAACAAATATCCTCCGCTGAGTACCTGAAACTCAAAGAGACGATCGCCGATGAGAATTGGTCGAAAGATGAAAATGCATTAGAGGTGGATTTTGAAGCATTTGATTTCTCGATGCCAAAATTAACATTGGCTTCTTCTATTGGAAAAGGACTTAATTTTGTGTCAAAGTACATCACTTCTAAGCTAAGTGGATCTGTGGATAATGCCCAGCCCCTTGTAGATTACTTACTCTCACTCGAATATCAAGGAGAGAAACTTATGATAAACGAGACACTTAACACAGCAGCAAAGCTTCAATTGGCTCTTATAGTAGCTGAAGTTTCCCTCTCAGATCTTCCTAGGGATACCCCATACCAGAGTATTGAGCTAAGGTTCAAGGAGTGGGGATTCGAGAGAGGGTGGGGTGATACGGTTGAAAGAGTGCATGAAACGATACGGTCACTCTCGGAAGTGTTGCAAGCACCTGATCCACAGAATTTGGAAAAGCTTTTTAGCAAACTTCCCACCATATTCAAGGTTGTAATCTTCTCTCCTCATGGATATTTTGGACAATCAGATGTGCTTGGTTTGCCAGACACTGGTGGACAGGTTGTTTATATTTTGGATCAAGTGAGGGCCATGGAGGAAGAATTGGTTCTCAAAATCAAATCCCAAGGCCTCAATATTAAGCCTCAAATCTTAGTGGTCACAAGACTCATACCTGATGCCCGAGGAACTAAGTGCAACCAAGAGAGGGAGTCCATCATCGGCACCAAATACAGTCAGATCCTCCGAGTGCCTTTCAGGACTGAAACCGGTATCCTACGCCGATGGGTTTCGCGTTTCGACATTTATCCTTATCTCGAGACGTTTGCTCAGGATGTTACATCCAAGATCTTGGATGCAATGGAGGGTAAACCAGACCTTATTATTGGAAACTACACTGATGGGAACTTAGTATCATCTCTAGTAGCTAGCAAACTTGGGATAACACAGGCTACGATTGCGCATGCTTTAGAGAAGACGAAATACGAGGATTCAGACATCAAGTGGAAGGAACTTGACCCAAAGTATCATTTTTCATGTCAATTCATTGCTGATACAATTGCAATGAATGCTGCAGATTTCATTATAGCAAGCACATACCAGGAGATTGCAGGGAGCAAAGAGAGACCTGGACAATATGAGAGCCATGCTGCATTTACACTCCCAGGGCTCTGTAGAGTTGTTTCAGGCATCAATGTTTATGACCCTAAGTTCAACATCGCTGCTCCCGGTGCCGATCAATCTGTGTATTTCCCGTATACGGAGACCGGAAAACGATTCACATCATTTCATCCTGCAATTGAAGAACTTCTGTACAGTAAAGTGGATAATGATGAACACATTGGTTATCTAGCAGACAGGAAGAAACCTATAATTTTCTCAATGGCAAGACTTGATACAGTGAAGAACTTGACTGGATTAACTGAATGGTACGGTAAAAACAAAAGGCTAAGAAGCTTGGTCAACCTTGTAATAGTAGGAGCCTTCTTTAATCCCTCTAAATCAAAAGATAGAGAAGAAATGGCTGAAATAAAAAAGATGCACGCACTCATAGAGAAATACCAACTCAAGGGCCAAATCCGATGGATAGCCGCGCAGACAGACCGTAACCGAAACGGCGAACTCTACCGTTGCATTGCCGATACAAAGGGTGCATTTGTTCAGCCAGCTTTATATGAAGCATTCGGTCTTACAGTGATCGAAGCAATGAACTGCGGTTTGCCGACCTTCGCAACCAATCAAGGAGGCCCTGCTGAGATCATTGTGGACGGAGTTTCCGGTTTCCATATTAATCCCACAAATGGAGATGAATCAAGCAACAAAATTGCTGATTTCTTTGAGAAATGCAAAACCAATCCTGCATATTGGAATCAGTTCTCAGCTGATGGATTGAAACGCATAAATGAATGCTATACCTGGAAAATATATGCAAACAAGGTATTAAATATGGGATGCATGTATGGGTTTTGGAAACAATTGAACAAAGATCAGAAACAAGCTAAACAGAGATACATTCAAGCATTTTATAATCTAATGTTCAGGAATCTGGTAAAAAATGTTCCTTTAGCAAGTGATGAAACTCAGCAACCAGACTCAAAGCCAGCAGGCAAACCACAGCCTACACCGAGGCATGTCTAA ATGACGTCTACATCGACCGGGAAGCTTAGTGACTCCATAGCTGACAACATACGCAATGCCTTGAAGCAGAGCCAGTCTTACATGAAACGTTGCTTTTCTAAGTACATGGAGAAAGGAAAAAGGGTTTTGAAAGCCCATGAATTGAGGGATGAATTTGAAAAAGTAATGGATGATAAAAATGAGACCTTGGGTACCATGTTTTCTTCAGCTCAGGTAAGTTTATTACATGCAAAAAAAGAAATGGAAAATGACATTTTTTGAAAGATTATGAGGTGTTTTATAATTTTCAGGAAGCGGTTGTTACTCCACCTTATGTCACCTTTGCCGTAAGACCGACTCCGGGATGTTGGGAGTTTGTTAAGGTGAACTCCGTCGATCTCTCCGACGTCAAACAAATATCCTCCGCTGAGTACCTGAAACTCAAAGAGACGATCGCCGATGAGAATTGGTATGTTAATTTATGCTATGGCATTGTATCTTCAAAAGGCGTATGTGTTTACGTCTGTGTGTGTATTTAAGTGTTTAATGTTTACTACAGGTCGAAAGATGAAAATGCATTAGAGGTGGATTTTGAAGCATTTGATTTCTCGATGCCAAAATTAACATTGGCTTCTTCTATTGGAAAAGGACTTAATTTTGTGTCAAAGTACATCACTTCTAAGCTAAGTGGATCTGTGGATAATGCCCAGCCCCTTGTAGATTACTTACTCTCACTCGAATATCAAGGAGAGGTATATCTATATATATGCATATGTACATATATGTACGATAAAATGCAAATTAATGGTTGTTTTCTCAACCTGCAGAAACTTATGATAAACGAGACACTTAACACAGCAGCAAAGCTTCAATTGGCTCTTATAGTAGCTGAAGTTTCCCTCTCAGATCTTCCTAGGGATACCCCATACCAGAGTATTGAGCTAAGGTAAAAACAGAATAGAAAAAGATATGGTTCCATCATGCTTGATGATTGCTTAGTATATGTGTATAAAATTTTGTTGCAGGTTCAAGGAGTGGGGATTCGAGAGAGGGTGGGGTGATACGGTTGAAAGAGTGCATGAAACGATACGGTCACTCTCGGAAGTGTTGCAAGCACCTGATCCACAGAATTTGGAAAAGCTTTTTAGCAAACTTCCCACCATATTCAAGGTTGTAATCTTCTCTCCTCATGGATATTTTGGACAATCAGATGTGCTTGGTTTGCCAGACACTGGTGGACAGGTTGTTTATATTTTGGATCAAGTGAGGGCCATGGAGGAAGAATTGGTTCTCAAAATCAAATCCCAAGGCCTCAATATTAAGCCTCAAATCTTAGTGGTGAATACAAATTTCTCCCCTATAGTAGCAATTTACAGTTAGTTTCTAGCCTGAATGGTGCTTGATGAATGGTAATATTTGAGCATAATAGGTCACAAGACTCATACCTGATGCCCGAGGAACTAAGTGCAACCAAGAGAGGGAGTCCATCATCGGCACCAAATACAGTCAGATCCTCCGAGTGCCTTTCAGGACTGAAACCGGTATCCTACGCCGATGGGTTTCGCGTTTCGACATTTATCCTTATCTCGAGACGTTTGCTCAGGCATGTGTAGATCTCTCTACTCATCCATCTGCTTCCATTTTGGTCTTACTAACTGCCCACTTTTATTTGGATATCATTAGGATGTTACATCCAAGATCTTGGATGCAATGGAGGGTAAACCAGACCTTATTATTGGAAACTACACTGATGGGAACTTAGTATCATCTCTAGTAGCTAGCAAACTTGGGATAACACAGGTGTCTGTTTAACTTACACACACACACACACACACATACATATATGCTAATTTCTAACAAGTATTTCTATGTAGGCTACGATTGCGCATGCTTTAGAGAAGACGAAATACGAGGATTCAGACATCAAGTGGAAGGAACTTGACCCAAAGTATCATTTTTCATGTCAATTCATTGCTGATACAATTGCAATGAATGCTGCAGATTTCATTATAGCAAGCACATACCAGGAGATTGCAGGGAGGTTAGAGAATTAGCCATATCGAACTCTGACTGTTCATTTTTCTTGAAGTACCAGATCCAATATTGCTCACTTGTAACTTATTTTACCATGATTTGTGACATAGAATTCAATCATGTTGTTTGCAGCAAAGAGAGACCTGGACAATATGAGAGCCATGCTGCATTTACACTCCCAGGGCTCTGTAGAGTTGTTTCAGGCATCAATGTTTATGACCCTAAGTTCAACATCGCTGCTCCCGGTGCCGATCAATCTGTGTATTTCCCGTATACGGAGACCGGAAAACGATTCACATCATTTCATCCTGCAATTGAAGAACTTCTGTACAGTAAAGTGGATAATGATGAACACATGTAAGTGAAGGTCTATTGAATTATATTTTGCTTCAAATCACTTGGGATTATTCAACATCTTATTGAATTATTTTGCTTCAAAACAGTGGTTATCTAGCAGACAGGAAGAAACCTATAATTTTCTCAATGGCAAGACTTGATACAGTGAAGAACTTGACTGGATTAACTGAATGGTACGGTAAAAACAAAAGGCTAAGAAGCTTGGTCAACCTTGTAATAGTAGGAGCCTTCTTTAATCCCTCTAAATCAAAAGATAGAGAAGAAATGGCTGAAATAAAAAAGATGCACGCACTCATAGAGAAATACCAACTCAAGGGCCAAATCCGATGGATAGCCGCGCAGACAGACCGTAACCGAAACGGCGAACTCTACCGTTGCATTGCCGATACAAAGGGTGCATTTGTTCAGCCAGCTTTATATGAAGCATTCGGTCTTACAGTGATCGAAGCAATGAACTGCGGTTTGCCGACCTTCGCAACCAATCAAGGAGGCCCTGCTGAGATCATTGTGGACGGAGTTTCCGGTTTCCATATTAATCCCACAAATGGAGATGAATCAAGCAACAAAATTGCTGATTTCTTTGAGAAATGCAAAACCAATCCTGCATATTGGAATCAGTTCTCAGCTGATGGATTGAAACGCATAAATGAATGGTAATAAAACAAGTTACAACCATGGCTGCTCATTTCTTTTGCAATATTAAAGCGCTGACTTGGGTATTTTTTTGGGGGGCTTGTAGCTATACCTGGAAAATATATGCAAACAAGGTATTAAATATGGGATGCATGTATGGGTTTTGGAAACAATTGAACAAAGATCAGAAACAAGCTAAACAGAGATACATTCAAGCATTTTATAATCTAATGTTCAGGAATCTGGTATATATATATATCTTTCTCTATTACTGGTTCACCTTTCATTGCTTTGATTTTATATCTGTTTATTAATATTAAAAAAACATTTTACAGGTAAAAAATGTTCCTTTAGCAAGTGATGAAACTCAGCAACCAGACTCAAAGCCAGCAGGCAAACCACAGCCTACACCGAGGCATGTCTAA null

***GhSus8At* A11** ATGGCTTCCAGACAATCACTCAAACGATCGGATACAATAGCTGAAAGCATGCCTGATGCTTTAAGGCAGAGCACCTCCCATATGAAGATATGTTTCAGCAGGTTGGTCGAATCGGGAAAACGGCTACTGAAACGTCAGCAGTTGATGGACGAAGTTGAGAATTCGATAGAAGACAAGGCAGAGAGAAGCAAGATCCTGGAGGGACTAATTGGTTTTATTCTTGTATCCACGCAGGAGGCTGCTGTCATTCCGCCGTACGTTGCTTTTGCTGTAAGACCGAACCCGGGATTCTGGGAGTTTGTCAAGGTGAACTCGGAAAACCTCCTTGTTGATGAAATTAACGCTTCAGAATACTTGAAGTGCAAGGAAATGGTGTTCGACAAAAATTGGGCAAAAGATGAGAATGCTTTGGAAATTGATTTTGCATCCATCAACCACAGTACCCCTCATTTAACCCTTCCTTCTAGCATTGGCAATGGAGCCAGCTACATCTCAAAGTTCATGTTCTCACAGTTATACGAGAGCTGCGACGGCGAGAAGCAGGTGTTGGACTACTTATTGTCCCTTAATCACCAAGGGGAGGATCTCCTGATAAACGGAAGTCTAAATACGGTTGACAAGCTTAAAACAGCTTTGAAGGCAGCTATCAGTATTATTTCGGAATTGCCCAAAACAACACCATATGAGAATTTTGAGCCAAGGTTGAAAGAGTTGGGTTTTGAGAAAGGATGGGGCGATAACGCAGAAAGAGCTAAAGAGAGTATGATGACACTTTACGAGGTGCTACAAATGCCAGAACCGGCAAATTTCGAGTCACTTTTTAGCTGGCTTCCGGCTGTGTTAAGGATCGTAATCCTGTCACCTCATGGTTACTTTGGGCAGTCGGATGTCCTCGGCTTGCCGGATACTGGAGGCCAGGTGGTTTACATTCTTGATCAAGTAAGAGCTCTTGAGGAAGTCTTGCTACGTAGAATAAAATCACGAGGCATGACCGTAAAGCCTCAGATTGTTGTGGTAACTCGCCTTATACCAGATGCTCGAGGAACCAAATGCAATCAAGAGATCGAACCGATCATCAATACCAAGCATTCCCACATTCTCAGAGTCCCATTCAGGACTGATAAAGGGGTGCTTCAGCAATGGGTCTCACGGTTCGACATCTATCCATATCTAGAGACATTTGCACAGGATGCTCAAGCAAAAGTCCTTCAACATATGGGATGTAAACCGGACCTCATAATCGGAAACTACAGCGACGGAAACTTGGTGGCATCTCTAATGGCTTCCAAACTTGGAATCACTCAGGGAACAATAGCTCATGCTTTAGAGAAAACCAAGTACGAAGATTCAGACGTGAAATGGAAAGAGGTTGATGCTAAGTATCACTTTTCATGTCAATATACAGCTGACATACTAGCAATGAATGCAGCTGATTTTATCATAACCAGCACCTATCAAGAAATCGCCGGAAGCACTGAAAAGCCTGGACAATATGAAAGTCATACAGCCTTTACCATGCCTGGACTATGCCGGGTTGTCTCTGGCATCAATATATTTGACCCCAAGTTCAACATATTACTCTACAGTCAGGACGATAACAATGAACACATAGGATATCTGGCGGACAGGAGGAAGCCGATTATCTTCTCTATGGCAAGACTTGATACTGTGAAGAATATTACAGGTCTAACTGAGTGGTATGGAAAGAATAAAAGGCTTAGAGATTTAGTAAATCTGGTTGTGGTTGCTGGTTTCTTTGATCCATCCAAGTCCAATGACAGAGAAGAACAAGCAGAAATTAAAAAGATGCATAGTCTGATGGAGCAATATCAACTTAGGGGTCAATTCAGATGGATTGCAGCCCAAACTGATAGACATCGCAACGGGGAGCTGTATCGATGCATTGCTGACACGAAAGGAGCTTTTGTGCAACCTGCCTTGTATGAGGCCTTTGGTCTAACTGTTATTGAAGCAATGAACTGTGGATTACCCACCTTTGCAACAAACAAAGGAGGTCCAGCAGAGATTATTGTGGATGGGGTCTCGGGTTTCCATATCGATCCGAACAATGGAGACCAATCCAGCAACACCATAGCTGATTTCTTTGAGAAGTGCAAGATGGATGCTCAACATTGGAACAGGGTGTCAACTCAAGGACTTCATCGCATACATGAATGCTACACATGGGAGATCTATGCAAACAAACTGTTGAACGTGGGATCCATGTATGGATTTTGGAGGCAGTTGAACAAAGAACAGAATCTAGCTAAACAAAGATACATTCAACTGCTCTTCAATCTGCAATTCAGGAAGTTGGCAAAGACTGTGCCTGTTCCAAAATAA ATGGCTTCCAGACAATCACTCAAACGATCGGATACAATAGCTGAAAGCATGCCTGATGCTTTAAGGCAGAGCACCTCCCATATGAAGATATGTTTCAGCAGGTACATACATTACATACATACATTCACACATTTATATATAGTTTTATATAATAACATGCGAAATCGAAAAAAAAGGTTGGTCGAATCGGGAAAACGGCTACTGAAACGTCAGCAGTTGATGGACGAAGTTGAGAATTCGATAGAAGACAAGGCAGAGAGAAGCAAGATCCTGGAGGGACTAATTGGTTTTATTCTTGTATCCACGCAGGTCTTTAAGTTTTAATTAATGCTATCATTTTTGGGGGGTATAGCTGGTATGCATGCATGCATTTTAGCTGTAAATATATATTTTTTGCAGGAGGCTGCTGTCATTCCGCCGTACGTTGCTTTTGCTGTAAGACCGAACCCGGGATTCTGGGAGTTTGTCAAGGTGAACTCGGAAAACCTCCTTGTTGATGAAATTAACGCTTCAGAATACTTGAAGTGCAAGGAAATGGTGTTCGACAAAAATTGGTACATAATTTATGTTACTTATCTCTGCAGTTTAAATTGGATATCCCTAGAACGTTCTTTAAGTATCATATTATTATGCTATGGTGCAGGGCAAAAGATGAGAATGCTTTGGAAATTGATTTTGCATCCATCAACCACAGTACCCCTCATTTAACCCTTCCTTCTAGCATTGGCAATGGAGCCAGCTACATCTCAAAGTTCATGTTCTCACAGTTATACGAGAGCTGCGACGGCGAGAAGCAGGTGTTGGACTACTTATTGTCCCTTAATCACCAAGGGGAGGTAAATACACATGCGCACATGCACATACACATACACACACATATGTTTGTATTGCAAACAACATTGAGAATCATACATGAATGTGCTTGGATTTTCAGGATCTCCTGATAAACGGAAGTCTAAATACGGTTGACAAGCTTAAAACAGCTTTGAAGGCAGCTATCAGTATTATTTCGGAATTGCCCAAAACAACACCATATGAGAATTTTGAGCCAAGGTTATATATATTTAGATTGCAACTACTACTTCATGCATAATTTACTTAGTTTCTGAATGGTGGCAATGAATATCAGGTTGAAAGAGTTGGGTTTTGAGAAAGGATGGGGCGATAACGCAGAAAGAGCTAAAGAGAGTATGATGACACTTTACGAGGTGCTACAAATGCCAGAACCGGCAAATTTCGAGTCACTTTTTAGCTGGCTTCCGGCTGTGTTAAGGATCGTAATCCTGTCACCTCATGGTTACTTTGGGCAGTCGGATGTCCTCGGCTTGCCGGATACTGGAGGCCAGGTACGACATAATTAAAACATAAAGATGTGGTTCAACTGAGGTAATTAAAGTACTTACAAAGAATTGTTTTTGCAAATGTAATAGGTGGTTTACATTCTTGATCAAGTAAGAGCTCTTGAGGAAGTCTTGCTACGTAGAATAAAATCACGAGGCATGACCGTAAAGCCTCAGATTGTTGTGGTAACTCGCCTTATACCAGATGCTCGAGGAACCAAATGCAATCAAGAGATCGAACCGATCATCAATACCAAGCATTCCCACATTCTCAGAGTCCCATTCAGGACTGATAAAGGGGTGCTTCAGCAATGGGTCTCACGGTTCGACATCTATCCATATCTAGAGACATTTGCACAGGTATATACTGTCTTGCCTCTGTTATGTTCTAATTCCATGGACTTAAAAACTTACTTGTTATCAATGTGTCTCTACGAGTAGGATGCTCAAGCAAAAGTCCTTCAACATATGGGATGTAAACCGGACCTCATAATCGGAAACTACAGCGACGGAAACTTGGTGGCATCTCTAATGGCTTCCAAACTTGGAATCACTCAGGGAACAATAGCTCATGCTTTAGAGAAAACCAAGTACGAAGATTCAGACGTGAAATGGAAAGAGGTTGATGCTAAGTATCACTTTTCATGTCAATATACAGCTGACATACTAGCAATGAATGCAGCTGATTTTATCATAACCAGCACCTATCAAGAAATCGCCGGAAGGTTATTTAGCTTTTATATGTTTATCATGTTATACAACATTAGACTGATTTTATATTAACTAAAAAGTCGTGAATTTCCTATTTGAAACAGCACTGAAAAGCCTGGACAATATGAAAGTCATACAGCCTTTACCATGCCTGGACTATGCCGGGTTGTCTCTGGCATCAATATATTTGACCCCAAGTTCAACATAGTTGCCCCAGGGGCTGACCAGTCGGTCTACTTTCCCTACACAGAGAAAAACAGGCGTTTATCCTCTTTCCATCCAGCCATTGAAGAGTTACTCTACAGTCAGGACGATAACAATGAACACATGTAAGTACGGAGCGATTAGTATCTGCATTGTATGTTCGTTGTTAGTTGGAATTAAAGGCGATTTTAATCATGTTATGCTTGCAACAGAGGATATCTGGCGGACAGGAGGAAGCCGATTATCTTCTCTATGGCAAGACTTGATACTGTGAAGAATATTACAGGTCTAACTGAGTGGTATGGAAAGAATAAAAGGCTTAGAGATTTAGTAAATCTGGTTGTGGTTGCTGGTTTCTTTGATCCATCCAAGTCCAATGACAGAGAAGAACAAGCAGAAATTAAAAAGATGCATAGTCTGATGGAGCAATATCAACTTAGGGGTCAATTCAGATGGATTGCAGCCCAAACTGATAGACATCGCAACGGGGAGCTGTATCGATGCATTGCTGACACGAAAGGAGCTTTTGTGCAACCTGCCTTGTATGAGGCCTTTGGTCTAACTGTTATTGAAGCAATGAACTGTGGATTACCCACCTTTGCAACAAACAAAGGAGGTCCAGCAGAGATTATTGTGGATGGGGTCTCGGGTTTCCATATCGATCCGAACAATGGAGACCAATCCAGCAACACCATAGCTGATTTCTTTGAGAAGTGCAAGATGGATGCTCAACATTGGAACAGGGTGTCAACTCAAGGACTTCATCGCATACATGAATGGTACCAAAACCATACACTAAAAGAGCACCAATGAACAATTTTCATTGAAGGAATAGCTTCAATTGATGTCTGTTTTTCTATTTATTTGAAAACCGCAGCTACACATGGGAGATCTATGCAAACAAACTGTTGAACGTGGGATCCATGTATGGATTTTGGAGGCAGTTGAACAAAGAACAGAATCTAGCTAAACAAAGATACATTCAACTGCTCTTCAATCTGCAATTCAGGAAGTTGGTGAGTGAACTTGGGACATTGTTATTTATAAATGGCAAGCGAATATAATTTTCTCACGCAAGTTAATGTAACAGGCAAAGACTGTGCCTGTTCCAAGTGAACAAGCCCTAGTATCTGTTCCAGTGCCACCTGAAACTCCTAAACCAGATACAGTTCCCGCTCCAGCACCTGATGCAGGACAACCCAAATCACAACCAGCTGTGCCTGGACCAAAAAGGCATGCCCTCTAACTTTTGGCTTCTACTCAAGCTTATATAATCTATAAGTACACTTAACCAAAAATATTTTGTCACAGGTCTCGAAGTTTGCGGAACATGGATGGCTCTCTTATGGAATTGTTCGTCATAGTTGGTTTCTTTTATCTCCTTTATTATTTCATAAAAAAGTTGTTTTACGGGCTTATGTGGTAAATGGTGATTGTATAAGTTGATGACAGACCAGAGCATTCTTTCAATTTGTATGCGTTTGTTAATGAAGAATAAGATACTTTCATATAATACATGATCAAAAAGTTCTTGCAGAAATGTACTGACTCCCTAGTTAGAATATTTTTATCCTTTTTACTTTTCATTGCTTTTTACCATTAAAATATGCTTCTAATAGTACGTTTTTATTCAAATGTTTGTCAAAAGCTGAATCTCTTGGCATTTGCTAAGATCTAAATTCTTCAAATGTTTTTATCGAATTAACTAGATTGTTAAATTTTTTATTTTTAAAAGTCCGATTAGTGGATAAATGCCAATCACGTAAAAAAACATACCTTAAATGAAAAATTTTAAATAATTAATGACCATTTTATAATTTTTTAAAATTAAGTGACCAAATAGTTTAATTACCTTTGATATAATTTACCCTTAATAAAATAAATAGAAAACGATTGGTACATCTTTGCAAATTCAAATTTCTATTCTGAAATTGTATAATGATTTTGTTAAACAGACTGACAGACTGCACAAACCCAGTGAGGAAGCAATCTATCTACAATTAGTTTAACTGCCAAACTAACATCTTCTATCTGTTTATTTGTAAAGCTTAACAACATAATATAATAAACTGAATTGAAATCCAATAATAA null

***GhSus8Dt* D11** ATGGCTTCCAGACAATCACTCAAACGATCGGATACAATAGCTGAAAGCATGCCTGATGCTTTAAGGCAGAGCACCTCCCATATGAAGATATGTTTCAGCAGGTTGGTCGAATCGGGAAAACGGCTACTGAAACGTCAGCAGTTGATGGACGAAGTTGAGAATTCGATAGAAGACAAGGCAGAGAGAAGCAAGATCCTGGAGGGACTAATTGGTTTTATTCTTGTATCCACGCAGGAGGCTGCTGTCATTCCGCCGTACGTTGCTTTTGCCGTAAGACCGAACCCGGGATTCTGGGAGTTCGTCAAGGTGAACTCGGAAAACCTCCTTGTTGATGAAATTAAAGTTTCAGAATACTTGAAGTGCAAGGAAATGGTTTTCGACCAAAATTGGGCAAAAGATGAGAATGCTTTGGAAATTGATTTTGCATCCATCAACCACAGTACCCCTCATTTAACCCTTCCTTCTAGCATTGGCAATGGAGCCAGCTACATCTCAAAGTTCATGTTCTCAAAGTTATACGAGAGCTGCGACGGTGAGAAGCAGGTGTTGGACTACTTATTGTCCCTTAATCACCAAGGGGAGGATCTCCTGATAAACGGAAATCTAAATACGGTTGACAAGCTTAAAACAGCTTTGAGGGCAGCTATCAGTATCATTTCGGAATTGCCCAAAACAACACCATATGAGAATTTTGAGCCAAGGTTGAAAGAGTTGGATTTTGAGAAAGGATGGGGCGATAACGCAGAAAGAGCTAAAGAGAGTATGATGACACTTTACGAGGTGCTACAAATGCCGGAACCGGCAAATTTTGAGTCACTCTTTAGCTGGCTCCCGGCTGTGTTAAGGATCGTAATCCTGTCACCTCATGGTTACTTTGGGCAGTCGGATGTCCTTGGCTTGCCGGATACTGGAGGCCAGGTGGTTTACATTCTTGATCAAGTAAGAGCTCTTGAGGAAGCCTTGCTACGTAGAATAAAATCACGAGGCATGACCGTAAAGCCTCAGATTGTTGTGGTAACTCGCCTTATACCAGATGCTCGAGGAACCAAATGCAATCAAGAGATCGAGCCGATCATCAATACCAAGCATTCCCACATTCTCAGAGTCCCGTTCAGGACTGATAAAGGGGTGCTTCAGCAATGGGTCTCACGGTTCGACATCTATCCATATCTAGAGACATTTGCACAGGATGCTCAAGCAAAAGTCCTTCAACATATGGGATGTAAACCGGACCTCATAATCGGAAACTACAGCGACGGAAATTTGGTGGCATCTCTAATGGCTTCCAATCTTGGAATCACTCAGGGAACAATAGCTCATGCTTTAGAGAAAACCAAGTATGAAGATTCAGATGTGAAATGGAAAGAGGTTGATGCTAAGTATCACTTTTCATGTCAATATACAGCTGACATACTAGCAATGAATGCAGCTGATTTTATCATAACAAGCACCTATCAAGAAATTGCCGGAAGCACTGAAAAGCCTGGACAATATGAAAGTCATACAGCCTTTACCATGCCTGGACTATGCCGGGTTGTCTCTGGCATCAATATATTTGACCCCAAGTTCAACATAGTTGCCCCAGGGGCTGACCAGTCGTTACTCTACAGTCAGGACGATAACAATGAGCACATAGGATATCTGGCGGACAGGAGGAAGCCGATTATCTTCTCTATGGCAAGACTTGATACTGTGAAGAATATTACAGGTCTAACTGAGTGGTATGGAAAGAATAAAAGGCTTAGAGATTTAGTAAATCTGGTTGTGGTTGCTGGTTTCTTTGATCCATCCAAGTCCAATGACAGAGAAGAACAAGCAGAAATTAAAAAGATGCATAGTCTGATGAAGCAATATCAACTTAGGGGTCAATTCAGATGGATTGCAGCCCAAACTGATAGACATCGCAACGGGGAGCTGTATCGATGCATTGCTGACACGAAAGGAGCTTTTGTGCAACCTGCCTTGTATGAGGCCTTTGGTCTAACTGTTATTGAAGCAATGAACTGTGGATTACCCACCTTTGCAACTAACAAAGGAGGTCCAGCAGAGATTATTGTGGATGGGGTCTCGGGTTTCCATATCGATCCGAACAATGGAGACCAATCCAGCAACACCATAGCTGATTTCTTTGAGAAGTGCAAGATGGATGCTCAACATTGGAACAGGGTGTCAACTCAAGGACTTCATCGCATACATGAATGCTACACATGGGAGATCTATGCAAACAAACTGTTGAACATGGGATCCATGTATGGATTTTGGAGGCAGTTGAACAAAGAACAGAATCTAGCTAAACAAAGATACATTCAACTGCTCTTCAATCTGCAATTCAGGAAGTTGGCAAAGACTGTGCCTGTTCCAATTAATGAAGAATAA ATGGCTTCCAGACAATCACTCAAACGATCGGATACAATAGCTGAAAGCATGCCTGATGCTTTAAGGCAGAGCACCTCCCATATGAAGATATGTTTCAGCAGGTACATACATAACATACATACATTCACACATTTATATATAGTTTGATCCTCTAACTAATAACATGCGAAATCGAAAAAAAAGGTTGGTCGAATCGGGAAAACGGCTACTGAAACGTCAGCAGTTGATGGACGAAGTTGAGAATTCGATAGAAGACAAGGCAGAGAGAAGCAAGATCCTGGAGGGACTAATTGGTTTTATTCTTGTATCCACGCAGGTCTTTAAGTTTTAATTAATGCTATCATTTTTGGGGGGTATAGCTGGTATGCATGCATGCATTTTAGCTGTAAATTTATATATATATTTTTTGCAGGAGGCTGCTGTCATTCCGCCGTACGTTGCTTTTGCCGTAAGACCGAACCCGGGATTCTGGGAGTTCGTCAAGGTGAACTCGGAAAACCTCCTTGTTGATGAAATTAAAGTTTCAGAATACTTGAAGTGCAAGGAAATGGTTTTCGACCAAAATTGGTACATAATTCATGTTACTTATCTCTGCAGTTTAAATTGGATATCCCTAGAAAGTTCTTTAAGTATCATATTATTATGCTATGGTGCAGGGCAAAAGATGAGAATGCTTTGGAAATTGATTTTGCATCCATCAACCACAGTACCCCTCATTTAACCCTTCCTTCTAGCATTGGCAATGGAGCCAGCTACATCTCAAAGTTCATGTTCTCAAAGTTATACGAGAGCTGCGACGGTGAGAAGCAGGTGTTGGACTACTTATTGTCCCTTAATCACCAAGGGGAGGTAAATACACATGTGCACATACACATACACATACACACACATATGTTTGTATTGCAAACAACATTGAGAATCATACATGAATGTGCTTGGAGTTTCAGGATCTCCTGATAAACGGAAATCTAAATACGGTTGACAAGCTTAAAACAGCTTTGAGGGCAGCTATCAGTATCATTTCGGAATTGCCCAAAACAACACCATATGAGAATTTTGAGCCAAGGTTATATATATTTAGATTGCAACTACTACTTCATGCATAATTTACTTATTTTCTGAATGGTGGCAATGAATATCAGGTTGAAAGAGTTGGATTTTGAGAAAGGATGGGGCGATAACGCAGAAAGAGCTAAAGAGAGTATGATGACACTTTACGAGGTGCTACAAATGCCGGAACCGGCAAATTTTGAGTCACTCTTTAGCTGGCTCCCGGCTGTGTTAAGGATCGTAATCCTGTCACCTCATGGTTACTTTGGGCAGTCGGATGTCCTTGGCTTGCCGGATACTGGAGGCCAGGTACAACATAATTAACATAAAGATGTGGTTCAACTGAGGTAATTAAAGTACTTACACAGAATTTTTTTTGCAAATGTAATAGGTGGTTTACATTCTTGATCAAGTAAGAGCTCTTGAGGAAGCCTTGCTACGTAGAATAAAATCACGAGGCATGACCGTAAAGCCTCAGATTGTTGTGGTAACTCGCCTTATACCAGATGCTCGAGGAACCAAATGCAATCAAGAGATCGAGCCGATCATCAATACCAAGCATTCCCACATTCTCAGAGTCCCGTTCAGGACTGATAAAGGGGTGCTTCAGCAATGGGTCTCACGGTTCGACATCTATCCATATCTAGAGACATTTGCACAGGTATATACTGTCTTGCCTCTGTTATGTTCTAATTCCATGGACTTAAAACTTACTTGTTATCAATGTGTCTCTACGACTAGGATGCTCAAGCAAAAGTCCTTCAACATATGGGATGTAAACCGGACCTCATAATCGGAAACTACAGCGACGGAAATTTGGTGGCATCTCTAATGGCTTCCAATCTTGGAATCACTCAGGGAACAATAGCTCATGCTTTAGAGAAAACCAAGTATGAAGATTCAGATGTGAAATGGAAAGAGGTTGATGCTAAGTATCACTTTTCATGTCAATATACAGCTGACATACTAGCAATGAATGCAGCTGATTTTATCATAACAAGCACCTATCAAGAAATTGCCGGAAGGTTATTTAGCTTTTTATATGTTTATCATGTTATACAACATTAGACTGATTTTATATTAACTAAAAAGTGGTGAATTTCCTATTTGAAACAGCACTGAAAAGCCTGGACAATATGAAAGTCATACAGCCTTTACCATGCCTGGACTATGCCGGGTTGTCTCTGGCATCAATATATTTGACCCCAAGTTCAACATAGTTGCCCCAGGGGCTGACCAGTCGGTCTACTTTCCCTACACAGAGAAAAACAGGCGTTTATCCTCTTTTTATCCAGCCATTGAAGAGTTACTCTACAGTCAGGACGATAACAATGAGCACATGTAAGTACGGAGCGATTAGTATCTGCATTGTATGTTCGTTGTTAGTTGGAATTAAAGGCGATTTTAATCATGTTATGCTTGCAACAGAGGATATCTGGCGGACAGGAGGAAGCCGATTATCTTCTCTATGGCAAGACTTGATACTGTGAAGAATATTACAGGTCTAACTGAGTGGTATGGAAAGAATAAAAGGCTTAGAGATTTAGTAAATCTGGTTGTGGTTGCTGGTTTCTTTGATCCATCCAAGTCCAATGACAGAGAAGAACAAGCAGAAATTAAAAAGATGCATAGTCTGATGAAGCAATATCAACTTAGGGGTCAATTCAGATGGATTGCAGCCCAAACTGATAGACATCGCAACGGGGAGCTGTATCGATGCATTGCTGACACGAAAGGAGCTTTTGTGCAACCTGCCTTGTATGAGGCCTTTGGTCTAACTGTTATTGAAGCAATGAACTGTGGATTACCCACCTTTGCAACTAACAAAGGAGGTCCAGCAGAGATTATTGTGGATGGGGTCTCGGGTTTCCATATCGATCCGAACAATGGAGACCAATCCAGCAACACCATAGCTGATTTCTTTGAGAAGTGCAAGATGGATGCTCAACATTGGAACAGGGTGTCAACTCAAGGACTTCATCGCATACATGAATGGTACCAAAACCATACACTAAAATAGCACCAATGAACAATTTTCATTGAAGGAATAGCTTCAATTGATGTCTGTTTTTTTAATTATTTGAAAACCGCAGCTACACATGGGAGATCTATGCAAACAAACTGTTGAACATGGGATCCATGTATGGATTTTGGAGGCAGTTGAACAAAGAACAGAATCTAGCTAAACAAAGATACATTCAACTGCTCTTCAATCTGCAATTCAGGAAGTTGGTGAGTGAACTTAGGCCATTATTATTTATAAATGGCAAGCGAATACAATTTTCTCACACAAGTTAATGTAACAGGCAAAGACTGTGCCTGTTCCAAGTGAGCAAGCCCTAGTATCTGTTCCAGTGCCACCTGAAACTCCTAAACCAGATACAGTTCCCGCTCCAGCACCTGATGCAGGACAACCCAAATCACAACCAGCTGTGCCTAGACCAAAAAGGCATGCCCTAACTTTTGGCTTCTACTCAAGCTTATATAATCTATAAGTACACTTAACCAAAAATATTTTGTCACAGGTCTCCAAGTTTGCGGAACATGGATGGCTCTCTTATGGAATTGTGCGTCATAGTTGGTTTCTTTTATCTTGTTTATTATTTCATAAAAAAGTTGTTTTACGGGCTTATGTGGTAAATGGTGATTGTATAAGTTGATGACAGACCAGAGCATTCTTTCAATTTGTATGCGTTAGTTAATGAAGAATAAGATACTTTCAAAGCTTTAATTAATACGTGATCAAAAAGTTCTTGCAGAAATGTACTGACTCCCTAGTTAGAATATTTTCATCATTTTTACTTTTCATAGCTTTTTACCATTAAATATGCTTCTAATAGTACGTCTTTATCCAAATGCTTGTCAAAAGCTGAATCTCTTGGCATTTGCTAAGATCTAAATTCTTCAAATGTTTTTATTAAATTGACTAGATTGAAAATTTTTTTATTTTTAAAAGTCCGATTAATGGATCAATACCAATCAAGTAAAAGAACATTCCTTAAATGAAAATTTTTAAATAATTAATGATTATTTTGCAATTTTTTAAAGTTGAGTGACCAAATAGTTTAATTACCTTTGATGTAAGCTACCCTTAATAAAATAAATAGAAAACGACGGGTACATCTTTGCAATTTCAAATTTCTATTCTGAAATTGTATAATGATTTTGTTAAACAGACTGACAGACTGCACAAACCTAGTGAGGAAGCAATCTATCTACAATTAGTTTAACTGCCAAACTAACATCTTCTATCTGTTTATTTGTAAAGCTTAACAACATAATATAATAAACTGAATTGAAATCCAATAATAATCTGTGAAACCTAAACATCCTACACCACAACACTAGGATAATTGCCTTTAACAAACCCTCAAATGGTCTTCTTGGGGGGGGGCACATAGCACACCACCAACTCTAGGCTGAAACTACCAGGGTCTCTTCTTTGCACCGAGAGATACAGCATTTGATCCTGATATCATAGCCCGGTTACCGTAGCTGCCACGTCCTCGGGCACGAAAGTTTCCTCCAGAACCTAAAACCAAAACAAGTACCATTGATCATCTAGAACCAAGAATATACCCAGGAAGTCTTGTTCACACGTTTGTTTTGCCAATAAGCATTCAAACAAAGTATTACCTCCAAACTTCGAACCTGCAGAACGAGCCAATGTAGACAATGCGGGGGAGACAACCTGCCCAGCATCTTGCAGGAGC null

Note： * means these gene sequence can be accessed from NCBI GenBank. §means only cDNA sequence can be accessed from Zou et al (2013) but no genomic DNA sequences can be accessed.

**Table S5 Primers were used in EcoTILLING analysis**

| Gene | Primer No. | Forward | Reverse | TM | Length(bp) |
| --- | --- | --- | --- | --- | --- |
| *GhSus1At* | a1^*^ | GTTAAGTTCAAAGAAATGGCTGGTCG | TTGCAATAAAAGCAAATGAGGCATACCTTG | 67 | 890 |
|  | a2^**^ | GTTAAGTTCAAAGAAATGGCTGGTCG | GCTCTTCCTTGAAGTGGAGATACTCAGCAAC | 67 | 569 |
|  | b1 | GGGATTACAAGGTGTTGATTTTAGGGTCATTTTTAT | GGAGTTGGATCATCTCGAGCACGC | 64 | 830 |
|  | b2 | GGGATTACAAGGTGTTGATTTTAGGGTCATTTTTAT | TGAAGAGCATTCAAGTTCTGAATTCTGTCATTC | 64 | 680 |
|  | c1 | GAAAGAGGTTGGGGTGACACCGAAG | TGTAAGGTAATCCGACCCGACCGAA | 67 | 816 |
|  | c2 | GAAAGAGGTTGGGGTGACACCGAAG | GCAACTCTTTGGAGATTTCATGAGCAACA | 67 | 657 |
|  | d1 | CCATACTTGGAAACCTACACAGAGGTGAAACAC | TTCTCAACTTTGCTGTAAAGAAGGTCTTCGATC | 65 | 804 |
|  | d2 | CCATACTTGGAAACCTACACAGAGGTGAAACAC | TACAACACGGTAGAGACCAGGAAGAGTGAAAG | 65 | 657 |
|  | e1 | AGACGGGATATTTTCCCACAATCGGATAC | TGTTGCTCATGAAATCTCCAAAGAGTTGC | 65 | 766 |
|  | e2 | AGACGGGATATTTTCCCACAATCGGATAC | TTCGGTCGGGTCGGATTACCTTACA | 65 | 605 |
|  | f1 | GGATCTTTCTTACACTTTTCAAAGAAATCGTCG | TGAGATATTTTATTAATGCTCGGCGGATAATGA | 65 | 895 |
|  | f2 | GGATCTTTCTTACACTTTTCAAAGAAATCGTCG | CTTTCACTCTTCCTGGTCTCTACCGTGTTGTA | 65 | 795 |
|  | g1 | TTTGCCAACATTCGCAACCCGT | GCACAACCCCATGTTCAAACAATGC | 67 | 689 |
|  | g2 | TTTGCCAACATTCGCAACCCGT | AAGACAAGATGAAATACAAAGGAGCTTCTCCAATT | 67 | 586 |
| *GhSus1Dt* | a1 | CGTCCACAGTCTCCGTGAGCGTTAA | ACCTTGCCCTTGTGGCAATGGAC | 64 | 800 |
|  | a2 | CGTCCACAGTCTCCGTGAGCGTTAA | GCTCTTCCTTGAAGTGGAGATACTCAGCAAC | 64 | 529 |
|  | b1 | TTCACGCCCTTGTTGTTGAGGAACAT | AGCATCTCATTCTCCAAAGCTCGGACTT | 64 | 1000 |
|  | b2 | TTCACGCCCTTGTTGTTGAGGAACAT | TGAAGAGCATTCAAGTTCTGAATTCTGTCATTC | 64 | 556 |
|  | c1 | AAAGAGGTTGGGGTGACACCGCTC | TTGTACTGTAATCCGACCCGACCGAA | 66 | 817 |
|  | c2 | AAAGAGGTTGGGGTGACACCGCTC | GCAACTCTTTGGAGATTTCATGAGCAACA | 66 | 657 |
|  | d1 | CCATACTTGGAAACCTACACAGAGGTGAAACAT | TTCTCAACTTTGCTGTAAAGAAGGTCTTCGATC | 66 | 804 |
|  | d2 | CCATACTTGGAAACCTACACAGAGGTGAAACAT | TACAACACGGTAGAGACCAGGAAGAGTGAAAG | 66 | 657 |
|  | e1 | GCCCATGCTTTGGAGAAGACAAAATATCTT | TTCATTTCGGCCTTCTCTTCCAAATCTTTA | 66 | 769 |
|  | e2 | GCCCATGCTTTGGAGAAGACAAAATATCTT | GACGAGTCCGGTTAAGTTCTTGACACGAT | 66 | 660 |
|  | f1 | ATGGATCTTTCTTACACTTTTCAAAGAAATCGTCC | CTTTCACTCTTCCTGGTCTCTACCGTGTTGTA | 66 | 797 |
|  | f2 | ATGGATCTTTCTTACACTTTTCAAAGAAATCGTCC | GATCGAAGACCTTCTTTACAGCAAAGTTGAGAA | 66 | 649 |
|  | g1 | TGCCAACATTCGCAACCGGC | GGCGCACAACCCCATGTTCAA | 67 | 699 |
|  | g2 | TGCCAACATTCGCAACCGGC | GCACAACCCCATGTTCAAACAATGC | 67 | 690 |
| *GhSus2At* | a1 | GAATTCTCCCGAGGAACTTCTCAAGGCTA | TGCATGCAAGTTTTGAAATTGTTGGCT | 67 | 1058 |
|  | a2 | GAATTCTCCCGAGGAACTTCTCAAGGCTA | CTGAAAGCCAGTCAGGTTTAAACAAAACCC | 67 | 901 |
|  | b1 | CTCGAGGCACCCGATCCTGGT | AAAAATATCAAATCCGAACATCTACCTTCCAGC | 67 | 1065 |
|  | b2 | CTCGAGGCACCCGATCCTGGT | CCTCGAGCTTCTTCCAGTAGATGTCGG | 67 | 945 |
|  | c1 | AATTCCGTGAACAACACGGTAGAGAACG | ACACCGGTGGCCAGGTTAGCAATTA | 67 | 945 |
|  | c2 | AATTCCGTGAACAACACGGTAGAGAACG | ATTCGGTTTTATTTGATTTCGGCTGTTATTCAT | 67 | 857 |
|  | d1 | CAATACGAGAGTCACACTGCTTTCACTCTTCAC | CTCAAGGTAACGACGGCTCTCGAGG | 67 | 966 |
|  | d2 | CAATACGAGAGTCACACTGCTTTCACTCTTCAC | TTGGGCAAAAAGAATCGCGGAAAT | 67 | 816 |
| *GhSus3At* | a1 | GTGATCACCCGAGTACACAGCCTCCTA | GTTCTGCAAAAACACCAACTAAGACATTCAGG | 67 | 938 |
|  | a2 | GTGATCACCCGAGTACACAGCCTCCTA | TTAGCTGAAAGGTGGCGATTGAGGAAC | 67 | 733 |
|  | b1 | TTTCAGCTAAATTGTTCCATGACAAGGAGTGT | CGTGAGCAACATCCTAAAGCCAAAGAGAT | 65 | 1080 |
|  | b2 | TTTCAGCTAAATTGTTCCATGACAAGGAGTGT | GCCAGACTTCGAATCTCGAGATCCATC | 65 | 970 |
|  | c1 | CGACTACGAGGTTAACCAACTCGCCC | AAGTCTGGCCCTACTTGGAAACTTACACTGAG | 67 | 1004 |
|  | c2 | CGACTACGAGGTTAACCAACTCGCCC | ATTTCCAAAGAGTTGCAAGGCAAGCC | 67 | 877 |
|  | d1 | TAACCGGACTCGTCGAGTGGTACTGC | TGTCCGGCCATAAATATGAAAACAACAAAA | 66 | 992 |
|  | d2 | TAACCGGACTCGTCGAGTGGTACTGC | TGCGGTACTTAAGAGCGTAAAACATCTCCA | 66 | 730 |
| *GhSus3Dt* | a1 | GATCACCCGAGTACACAGCCTCTGG | CCGATTTCCTGGAACTTGTGTTCGAGT | 67 | 1053 |
|  | a2 | GATCACCCGAGTACACAGCCTCTGG | TTAGCCGAAAGGTGGCGGTTGAG | 67 | 724 |
|  | b1 | AGTTCCTCAACCGCCACCTCTCG | GCCAGACTTCGAATCTCGAGATCCATC | 67 | 989 |
|  | b2 | AGTTCCTCAACCGCCACCTCTCG | GTAATCTGATCAGCAACAACAACCAAAATCAGATA | 67 | 842 |
|  | c1 | CACCTACGACTACAAGGTTAACCAACTCTCGA | AAGTCTGGCCCTACTTGGAAACTTACACTGAG | 67 | 1004 |
|  | c2 | CACCTACGACTACAAGGTTAACCAACTCTCGA | ATTTCCAAAGAGTTGCAAGGCAAGCC | 67 | 877 |
|  | d1 | CTTAACCGGACTCGTCGAGTGGTACAGA | AAAACCCGCCTCTTCCTTTGTTTTACATCT | 67 | 891 |
|  | d2 | CTTAACCGGACTCGTCGAGTGGTACAGA | ACCAGCTTGCGGTACTTAAGAGCATAAAACA | 67 | 734 |
| *GhSus4At* | a1 | GTTGATTTTGGTCATATTTGTGTTATAGGAAGGAAC | CTTGTTGTTTGATACGGTGGATCATCTCG | 67 | 865 |
|  | a2 | GTTGATTTTGGTCATATTTGTGTTATAGGAAGGAAC | TGTGGGGAAAGTATGACAACATTGAATACCATA | 67 | 746 |
|  | b1 | CGGACCTTCTCGAGGCACCTATCC | TATACCTTCCTGCAATTTCTTGGAAAGTACTTGTG | 67 | 929 |
|  | b2 | CGGACCTTCTCGAGGCACCTATCC | TCCAAAGCGTGGGCGATCGTAC | 67 | 780 |
|  | c1 | TCGGGTTCTTCCCATACCACTCAATG | ACAGAAAACGGAATCGTACGTCAATGGA | 67 | 1044 |
|  | c2 | TCGGGTTCTTCCCATACCACTCAATG | GGTGAACCATATACATACCGTTGATTTCACTTTG | 67 | 973 |
|  | d1 | GCTAGACCGTGTCAAGAACTTAACTGGCATC | GAAAAAGCCACATCAGCTTACTAACAGAATCTTTTC | 67 | 943 |
|  | d2 | GCTAGACCGTGTCAAGAACTTAACTGGCATC | GGACGTGAGAAATACCAGCTTCCGGT | 67 | 771 |
| *GhSus4Dt* | a1 | TGATTTTGGTCATATTTGTGTTGTAGGAAGCACT | GCTGGATCATCTCGTTCTCCAAGGC | 67 | 849 |
|  | a2 | TGATTTTGGTCATATTTGTGTTGTAGGAAGCACT | TGTGGGGAAAGTATGACAACATTGAATACCATA | 67 | 745 |
|  | b1 | GGACCTTCTCGAGGCACCTCACG | TATACCTTCCTGCAATTTCTTGGAAAGTACTTGTG | 67 | 928 |
|  | b2 | GGACCTTCTCGAGGCACCTCACG | TCCAAAGCGTGGGCGATCGTAC | 67 | 780 |
|  | c1 | TTCGGGTTCTTCCCATACCACTCAAGA | ACAGAAAACGGAATCGTACGTCAATGGA | 67 | 1073 |
|  | c2 | TTCGGGTTCTTCCCATACCACTCAAGA | GTACGTCAATGGATCTCTAGATTCGAAGTTTGG | 67 | 1058 |
|  | d1 | GGACCGTGTCAAGAACTTAACTGGCCAT | AAAAGGAAATGGACGTGAGAAATACCAGCTT | 67 | 780 |
|  | d2 | GGACCGTGTCAAGAACTTAACTGGCCAT | GACGGCTCTTGTGGCGGTCAAG | 67 | 710 |
| *GhSus5Dt* | a1 | GGAACCTAAAAAACAATGGCTTCAATCAGTGAT | CCTTCTGGCAGCGAATTTGGAGAAAT | 67 | 795 |
|  | a2 | GGAACCTAAAAAACAATGGCTTCAATCAGTGAT | AATGGACTTTGAAAGAGATGGACGAGGAAC | 67 | 690 |
|  | b1 | AGGTAGGTAGTTTTCTCTTACAGCTCCTGGTTTAAA | TCCTTAGTGTAAGTCTCCAGGTAAGGCCAGAC | 67 | 1031 |
|  | b2 | AGGTAGGTAGTTTTCTCTTACAGCTCCTGGTTTAAA | GGTAATGTCGAGTCCTTGCAGCTTGAAA | 67 | 770 |
|  | c1 | GTTGCCACCTATTTGAGTCCCTAACAATGTATT | ACTTCTCCTGTATGCGTTTCAACCCTCC | 67 | 922 |
|  | c2 | GTTGCCACCTATTTGAGTCCCTAACAATGTATT | GTGTCGCAAATATAACGATAGAGTTCACCATTTCT | 67 | 658 |
|  | d1 | GAACTTATCGAAAAATACAAGTTGAATGGACAATCC | ATGGGACCAAACCCAGAGTTCAAAACAT | 67 | 797 |
|  | d2 | GAACTTATCGAAAAATACAAGTTGAATGGACAATCC | AACATTCCAAGGGGAAACCCCAAAGT | 67 | 774 |
| *GhSus6At* | a1 | ACTTCAACAGCAGTAACAGAGTAGATACGAAGGC | TCTGCAAGTGCTTCTTTGAAGCGAAGAT | 67 | 1082 |
|  | a2 | ACTTCAACAGCAGTAACAGAGTAGATACGAAGGC | CGCAGCATCGAGGTAAGGAAAGAACAT | 67 | 753 |
|  | b1 | GTATCCGATCATTCAACATCAATGCCTACAGTA | ATGTTCTTTCCTTACCTCGATGCTGCG | 67 | 920 |
|  | b2 | GTATCCGATCATTCAACATCAATGCCTACAGTA | GCTATTTAATTTTCACGCCTAACTTTCAGCCTTT | 67 | 850 |
|  | c1 | AATTCTTCTATTGAGCCATGCAGTGCCATA | TGGCCTTATCTGGAGACTTATGCGGAG | 67 | 1065 |
|  | c2 | AATTCTTCTATTGAGCCATGCAGTGCCATA | CTACAGTGACGGAAACCTGGTTGCATCT | 67 | 783 |
|  | d1 | CACATGTCATGGCTTCCACCGCT | AGACTTACGGCACTGCATGGCTCAATAG | 67 | 892 |
|  | d2 | CACATGTCATGGCTTCCACCGCT | TTGTTGTTTGATCCTAAGCAGAATGATGAACA | 67 | 857 |
|  | e1 | GCCTGCATTCTACGAAGCCTTTGGACTTATA | CCGAATTTACGATTGCAATTCTTACCACCT | 67 | 907 |
|  | e2 | GCCTGCATTCTACGAAGCCTTTGGACTTATA | GGCAGTCCAAAGCCAGAACACTGC | 67 | 823 |
| *GhSus6Dt* | a1 | ACTTCAACAGCAGTAACAGAGTAGATACGAAGGC | GCAGACGTTAAAAGCTTACCTAGTACAAAGGCTG | 66 | 857 |
|  | a2 | ACTTCAACAGCAGTAACAGAGTAGATACGAAGGC | GCAGCATCGAGGTAAGGAAAGAACACAA | 66 | 756 |
|  | b1 | CCGATCATTCAACATCAATGCCTACAGTG | TTGTGTTCTTTCCTTACCTCGATGCTGC | 67 | 924 |
|  | b2 | CCGATCATTCAACATCAATGCCTACAGTG | CAGCCTTTGTACTAGGTAAGCTTTTAACGTCTGC | 67 | 829 |
|  | c1 | CTTCTATTGAGCCATGCAGTGCCCTC | GTGAAATTGCAGCAGAGTTGCAGGGTA | 67 | 824 |
|  | c2 | CTTCTATTGAGCCATGCAGTGCCCTC | ATAGGAAACTACAGTGACGGAAACCTAGTTGCA | 67 | 780 |
|  | d1 | CCACATGTCATGGCTTCCACCAAC | TCAAGTCAGCTGCTTTCCTATAGTAGGAGATGG | 67 | 1161 |
|  | d2 | CCACATGTCATGGCTTCCACCAAC | GCTCAATAGAAGAATTGTTGTTTGATCCTAAGCAG | 67 | 868 |
|  | e1 | CCTGCATTCTACGAAGCCTTTGGACTTATG | AGAACACTGCATTTATTTACAACACCCATCACATAT | 66 | 813 |
|  | e2 | CCTGCATTCTACGAAGCCTTTGGACTTATG | AATGCTCCATAGCAAGGAGACCTAATCTGC | 66 | 740 |
| *GhSus7At* | a1 | CTGGGCATTATCCACAGATCCACTTTGT | TGTGTTGTTAGACTGACATCCACGAAAAGAAA | 67 | 980 |
|  | a2 | CTGGGCATTATCCACAGATCCACTTTGT | TCATGCATTTGCATATATTTCATTATTCCTATCCC | 67 | 827 |
|  | b1 | CCGAGTACTTGAAACTCAAAGAGACGCC | GCGAAACCCATCGGCGTAGGA | 67 | 1148 |
|  | b2 | CCGAGTACTTGAAACTCAAAGAGACGCC | TTTGCTAAAAAGCTTCTCCAAATTCTGTGGAT | 67 | 735 |
|  | c1 | GAAACCCATCGGCGTAGGATACGA | CTTCAAAAGGTGTATGTGTTTATGTCCGTGTGT | 67 | 1073 |
|  | c2 | GAAACCCATCGGCGTAGGATACGA | GGATAATGCCCAGCCCCTTGTAGATTACTTA | 67 | 872 |
|  | d1 | AAATACAGTCAGATCCTCCGAGTGCCTTTCTA | GACCTTCACTTACATGTGTTCATCATTATCCACTTT | 67 | 896 |
|  | d2 | AAATACAGTCAGATCCTCCGAGTGCCTTTCTA | TGAAATGATGTGAATCGTTTTCCGGTCT | 67 | 855 |
|  | e1 | GGAAACCGGAAACTCCGTCCAGG | ATACCAGGAGATTGCAGGGAGGTTAGAGAATTA | 67 | 900 |
|  | e2 | GGAAACCGGAAACTCCGTCCAGG | ATATCGAACTCCGACTGTTCATTTTTCTTGAAGT | 67 | 864 |
|  | f1 | GAAGTTAGACATGCCTCGGTGTGGGT | ACTCATAGAGAAATACCAACTCAAGGGCCAAA | 67 | 777 |
|  | f2 | GAAGTTAGACATGCCTCGGTGTGGGT | AATACCAACTCAAGGGCCAAATCCGA | 67 | 763 |
| *GhSus7Dt* | a1 | GCTGGGCATTATCCACAGATCCACTTATC | CGGGTGGCCTAAGTTTCTTCTTACGTACAC | 67 | 909 |
|  | a2 | GCTGGGCATTATCCACAGATCCACTTATC | CTGCACTTTATCATATACCATAACCCCAACCC | 67 | 780 |
|  | b1 | GCCGAGTACCTGAAACTCAAAGAGACGTT | GCGAAACCCATCGGCGTAGGA | 67 | 1148 |
|  | b2 | GCCGAGTACCTGAAACTCAAAGAGACGTT | TCCTCCATGGCCCTCATTTGGTC | 67 | 863 |
|  | c1 | CGAAACCCATCGGCGTAGGATACTG | TCCGTCGATCTCTCCGACGTCAA | 67 | 1183 |
|  | c2 | CGAAACCCATCGGCGTAGGATACTG | TATTGGAAAAGGACTTAATTTTGCGTCAAAGTACAT | 67 | 934 |
|  | d1 | CAGATCCTCCGAGTGCCTTTCCG | GACCTTCACTTACATGTGTTCATCATTATCCACTTT | 67 | 896 |
|  | d2 | CAGATCCTCCGAGTGCCTTTCCG | TGAAATGATGTGAATCGTTTTCCGGTCT | 67 | 855 |
|  | e1 | ATATGGAAACCGGAAACTCCGTCCAAA | GGATTCAGACATCAAGTGGAAGGAACTTGA | 67 | 1008 |
|  | e2 | ATATGGAAACCGGAAACTCCGTCCAAA | ACTCTGACTGTTCGTTTTTCTTGAAGTACCAGATC | 67 | 865 |
|  | f1 | TTTAATGGAAGTTAGACATGCCTCGGTGTAGTC | ACTGAATGGTACGGTAAAAACAAAAGGCTAAGAAG | 66 | 899 |
|  | f2 | TTTAATGGAAGTTAGACATGCCTCGGTGTAGTC | AATACCAACTCAAGGGCCAAATCCGA | 66 | 763 |
| *GhSus8At* | a1 | TTAAGGGACAATAAGTAGTCCAACACCTGCTTCTAG | TATGATATCTTTGGGAAAAGGCAATCCCATTA | 67 | 1090 |
|  | a2 | TTAAGGGACAATAAGTAGTCCAACACCTGCTTCTAG | TTAAACTAAAATGGCTTCCAGACAATCACTCAAAC | 67 | 833 |
|  | b1 | AGCCAGCTACATCTCAAAGTTCATGTTCTCTC | TTACATCCCATATGTTGAAGGACTTTTGCTTG | 67 | 1086 |
|  | b2 | AGCCAGCTACATCTCAAAGTTCATGTTCTCTC | TCTGAGAATGTGGGAATGCTTGGTATTGAT | 67 | 883 |
|  | c1 | AGATACTAATCGCGCCGTACTTACATGGGT | CAGTCGGATGTCCTCGGCTTGC | 67 | 1099 |
|  | c2 | AGATACTAATCGCGCCGTACTTACATGGGT | ATCAATACCAAGCATTCCCACATTCTCAGA | 67 | 820 |
|  | d1 | CCTCCAAAATCCATACATGGATCCCTC | CAATATGAAAGTCATACAGCCTTTACCATGCCT | 67 | 1025 |
|  | d2 | CCTCCAAAATCCATACATGGATCCCTC | AAGTACGGCGCGATTAGTATCTGCATTGT | 67 | 815 |
| *GhSus8Dt* | a1 | ACAATAAGTAGTCCAACACCTGCTTCGCA | ATAATGTTCCTTTTCCTCTTCCTTTTCCTACTCTCA | 67 | 994 |
|  | a2 | ACAATAAGTAGTCCAACACCTGCTTCGCA | AATGCAGAGCACCTCCCATATGAAGATATGT | 67 | 786 |
|  | b1 | ATGTTCTCAAAGTTATACGAGAGCTGCGACTGT | AGCACCCCTTTATCAGTCCTGAACGG | 67 | 902 |
|  | b2 | ATGTTCTCAAAGTTATACGAGAGCTGCGACTGT | TCTGAGAATGTGGGAATGCTTGGTATTGAT | 67 | 873 |
|  | c1 | CATTCTTGATCAAGTAAGAGCTCTTGAGGATGC | TATCAAGTCTTGCCATAGAGAAGATAATCGGCTT | 67 | 1083 |
|  | c2 | CATTCTTGATCAAGTAAGAGCTCTTGAGGATGC | TTTCTCTGTGTAGGGAAAGTAGACCGACTGG | 67 | 870 |
|  | d1 | CCTCCAAAATCCATACATGGATCCCCT | CAATATGAAAGTCATACAGCCTTTACCATGCCT | 67 | 1025 |
|  | d2 | CCTCCAAAATCCATACATGGATCCCCT | TTGTATGTTCGTTGTTAGTTGGAATTAAAGGCAA | 67 | 789 |

Note: * and ** means that primers ending with 1 are the external ones and those ending with 2 are the internal ones.

**Table S6 Means, standard deviations and range for ten agronomic traits in 277 upland cotton accessions in 9 diverse environments**

| Trait | Environment | Mean | Min | Max | Std Dev | CV(%) |
| --- | --- | --- | --- | --- | --- | --- |
| FL | E1 | 28.77 | 20.51 | 35.28 | 1.63 | 5.67 |
|  | E2 | 29.72 | 20.77 | 36.45 | 1.81 | 6.08 |
|  | E3 | 27.88 | 18.50 | 31.99 | 1.31 | 4.71 |
|  | E4 | 29.09 | 20.33 | 33.46 | 1.56 | 5.35 |
|  | E5 | 29.74 | 21.29 | 34.70 | 1.58 | 5.33 |
|  | E6 | 28.71 | 22.12 | 33.37 | 1.24 | 4.32 |
|  | E7 | 29.19 | 20.99 | 33.67 | 1.53 | 5.25 |
|  | E8 | 28.95 | 22.38 | 34.14 | 1.41 | 4.87 |
|  | E9 | 28.38 | 18.18 | 34.68 | 1.69 | 5.95 |
| FS | E1 | 28.46 | 23.30 | 37.23 | 2.17 | 7.64 |
|  | E2 | 28.34 | 23.73 | 37.77 | 2.34 | 8.24 |
|  | E3 | 28.07 | 23.50 | 38.30 | 2.19 | 7.80 |
|  | E4 | 28.29 | 22.57 | 35.33 | 1.88 | 6.65 |
|  | E5 | 29.25 | 24.57 | 36.00 | 1.93 | 6.60 |
|  | E6 | 28.79 | 24.10 | 37.70 | 2.05 | 7.12 |
|  | E7 | 29.67 | 24.25 | 40.57 | 2.04 | 6.88 |
|  | E8 | 28.70 | 23.83 | 39.93 | 1.92 | 6.68 |
|  | E9 | 28.25 | 20.19 | 41.43 | 2.39 | 8.46 |
| FM | E1 | 4.66 | 3.47 | 5.89 | 0.39 | 8.27 |
|  | E2 | 5.02 | 2.45 | 6.80 | 0.56 | 11.21 |
|  | E3 | 4.40 | 3.05 | 5.78 | 0.44 | 9.93 |
|  | E4 | 4.10 | 3.11 | 5.28 | 0.34 | 8.28 |
|  | E5 | 4.34 | 3.31 | 5.26 | 0.33 | 7.68 |
|  | E6 | 4.42 | 3.47 | 5.30 | 0.29 | 6.62 |
|  | E7 | 4.47 | 3.09 | 6.04 | 0.46 | 10.37 |
|  | E8 | 4.12 | 2.70 | 5.48 | 0.61 | 14.73 |
|  | E9 | 4.82 | 3.30 | 5.89 | 0.45 | 9.40 |
| FU | E1 | 84.09 | 78.55 | 86.00 | 1.01 | 1.20 |
|  | E2 | 84.52 | 79.03 | 87.00 | 1.03 | 1.22 |
|  | E3 | 83.61 | 80.63 | 85.63 | 0.88 | 1.05 |
|  | E4 | 83.55 | 78.80 | 86.57 | 1.00 | 1.20 |
|  | E5 | 84.48 | 79.07 | 86.67 | 0.90 | 1.06 |
|  | E6 | 85.04 | 79.77 | 87.47 | 0.91 | 1.07 |
|  | E7 | 83.62 | 75.85 | 86.20 | 1.17 | 1.40 |
|  | E8 | 83.00 | 79.77 | 86.20 | 1.07 | 1.29 |
|  | E9 | 83.99 | 79.60 | 86.83 | 1.09 | 1.30 |
| LP | E1 | 38.10 | 24.05 | 48.64 | 3.68 | 9.67 |
|  | E2 | 37.52 | 22.21 | 48.32 | 3.64 | 9.69 |
|  | E3 | 35.48 | 24.59 | 43.75 | 3.18 | 8.95 |
|  | E4 | 38.57 | 22.13 | 47.43 | 3.57 | 9.26 |
|  | E5 | 39.24 | 19.96 | 47.27 | 3.45 | 8.80 |
|  | E6 | 39.02 | 22.34 | 45.99 | 2.81 | 7.20 |
|  | E7 | 36.40 | 20.64 | 46.46 | 3.94 | 10.82 |
|  | E8 | 36.47 | 21.52 | 49.67 | 3.97 | 10.89 |
|  | E9 | 36.42 | 21.05 | 45.03 | 3.71 | 10.19 |
| SI | E1 | 11.56 | 4.55 | 17.86 | 1.45 | 12.56 |
|  | E2 | 11.96 | 7.43 | 19.34 | 1.46 | 12.22 |
|  | E3 | 11.04 | 8.22 | 18.70 | 1.33 | 12.06 |
|  | E4 | 12.80 | 9.35 | 19.82 | 1.56 | 12.21 |
|  | E5 | 11.68 | 9.07 | 17.43 | 1.24 | 10.64 |
|  | E6 | 10.61 | 8.27 | 13.82 | 0.97 | 9.12 |
|  | E7 | 11.43 | 8.20 | 20.30 | 1.69 | 14.77 |
|  | E8 | 11.65 | 7.40 | 19.87 | 1.76 | 15.11 |
|  | E9 | 12.18 | 8.67 | 21.20 | 1.71 | 14.03 |
| BW | E1 | 5.68 | 3.20 | 7.65 | 0.70 | 12.28 |
|  | E2 | 6.02 | 3.32 | 8.60 | 0.70 | 11.61 |
|  | E3 | 5.40 | 3.24 | 7.38 | 0.62 | 11.43 |
|  | E4 | 5.97 | 3.43 | 8.23 | 0.73 | 12.24 |
|  | E5 | 5.97 | 3.62 | 8.72 | 0.64 | 10.77 |
|  | E6 | 5.46 | 2.86 | 7.22 | 0.56 | 10.19 |
|  | E7 | 5.31 | 2.42 | 7.51 | 0.72 | 13.55 |
|  | E8 | 5.54 | 3.26 | 7.62 | 0.72 | 13.00 |
|  | E9 | 5.45 | 2.81 | 7.26 | 0.64 | 11.66 |
| BN | E1 | 14.07 | 4.02 | 23.47 | 3.46 | 24.58 |
|  | E2 | 11.21 | 3.91 | 18.33 | 2.68 | 23.91 |
|  | E3 | 10.53 | 4.63 | 16.20 | 2.13 | 20.19 |
|  | E4 | 8.04 | 4.93 | 12.67 | 1.38 | 17.14 |
|  | E5 | 6.87 | 4.20 | 10.83 | 1.07 | 15.52 |
|  | E6 | 6.68 | 4.37 | 9.37 | 0.84 | 12.54 |
|  | E7 | 12.15 | 1.90 | 25.17 | 3.95 | 32.52 |
|  | E8 | 13.33 | 2.53 | 27.36 | 3.55 | 26.61 |
|  | E9 | 10.37 | 1.07 | 24.33 | 4.69 | 45.20 |
| PC | E3 | 39.44 | 32.85 | 45.93 | 2.91 | 7.37 |
|  | E6 | 41.48 | 34.54 | 48.50 | 2.97 | 7.16 |
|  | E9 | 39.99 | 34.27 | 46.83 | 2.85 | 7.13 |
| OC | E3 | 32.10 | 24.40 | 39.52 | 3.36 | 10.47 |
|  | E6 | 34.04 | 26.97 | 40.78 | 3.39 | 9.96 |
|  | E9 | 35.22 | 27.85 | 41.52 | 3.28 | 9.32 |

Note: E1~E3 means 2007~2009 in Anyang of Henan province, E4~E6 means 2007~2009 in Kuche of Xinjiang province,

E7~E9 means 2007~2009 in Nanjing of Jiangsu province.
